# Selection of a core collection of *Prunus sibirica* L. germplasm by a stepwise clustering method using simple sequence repeat markers

**DOI:** 10.1371/journal.pone.0260097

**Published:** 2021-11-19

**Authors:** Yongqiang Sun, Shengjun Dong, Quangang Liu, Jianhua Chen, Jingjing Pan, Jian Zhang

**Affiliations:** School of Forestry, Shenyang Agricultural University, Shenyang, China; National Cheng Kung University, TAIWAN

## Abstract

*Prunus sibirica* is an economically important tree species that occurs in arid and semi-arid regions of northern China. For this species, creation of a core collection is critical for future ecological and evolutionary studies, efficient economic utilization, and development and management of the broader collection of its germplasm resources. In this study, we sampled 158 accessions of *P*. *sibirica* from Russia and China using 30 pair of simple sequence repeat molecular markers and 30 different schemes to identify candidate core collections. The 30 schemes were based on combinations of two different sampling strategies, three genetic distances, and five different sample sizes of the complete germplasm resource. We determined the optimal core collection from among the 30 results based on maximization of genetic diversity among groups according to Number of observed alleles (N_a_), Number of effective alleles (N_e_), Shannon’s information index (I), Polymorphic information content (PIC), Nei gene diversity (H) and compared to the initial collection of 158 accessions. We found that the optimal core collection resulted from preferred sampling at 25% with Nei & Li genetic distance these ratios of N_a_, N_e_, I, PIC and H to the complete 158 germplasm resources were 73.0%, 113%, 102%, 100% and 103%, respectively, indicating that the core collection comprised a robust representation of genetic diversity in *P*. *sibirica*. The proposed core collection will be valuable for future molecular breeding of this species and management of its germplasm resources.

## Introduction

Siberian apricot (*Prunus sibirica*) is a shrub or tree species of the genus Rosaceae family. *P*. *sibirica* is widely distributed across a wide range of geographical areas in the northern regions of China and Russia [1]. Duo to its strong ecological adaptability, *P*. *sibirica* can be used as a pioneer tree species for improving the environment in semi-arid and arid areas [2]. Its kernels are of great economic value and have become an important industry for the people in the producing areas to get rid of poverty and become rich [3]. However, the cultivation of *P*. *sibirica* and its promotion within the horticultural and silvicultural industries still face challenges. In particular, the cultivar that high-quality and high-yield remain scarce, but this can be overcome by plant breeding research, especially molecular breeding approaches, to expand the scale of cultivation of *P*. *sibirica*.

Plant breeding process needs to rely on large-scale germplasm resources. Thus, the collection, evaluation, management and utilization of germplasm resources are supported by many governments or organizations worldwide [4–7]. As of 2016, there are more than 1300 germplasm resources preservation banks around the world, and approximately 6.1 million germplasm resources (including duplicates) have been preserved [8], but the quantity of these resources led to difficulties in management, housing, and efficient studies [4]. In response to this problem, the concept of the core collection was proposed by Frankel [9], and then gradually supplemented and perfected from three aspects, including basic characteristics, construction principles and methods [10–12]. Ideally, the core collection should represent the maximum level of genetic diversity of the initial collection with the smallest number of samples. The small but highly representative sample comprising the core can facilitate in-depth genomic investigation for molecular breeding as well as other applications [13].

Since the concept of the core collection was put forward, multiple types of data have been used to construct core collection, such as phenotypic traits, DNA molecular markers. Phenotypic traits result from underlying genotypes but are affected by compound interactions between the genotype and environment. Moreover, for trees, years of continuous investigation are often needed to obtain reliable phenotypic data [14]. DNA molecular markers, which are developed based on DNA polymorphisms, represent a direct method of measuring genetic diversity and are rarely affected by environmental interactions. Thus, they are more suitable for constructing a core collection and evaluating genetic diversity than phenotypic traits [15]. For the reason that molecular technology becomes more convenient and economical, DNA molecular markers have been an advantageous and ideal tool for the establishment of core collections [16,17].

According to the definition of core collection that the least amount of germplasm represents the genetic diversity of the initial collection to the maximum extent, so the selection of core collection should avoid the germplasm with close genetic relationship. In order to do that, an appropriate and efficient sampling strategy is critical during the establishment of core collection [18]. At the same time, for the sake of verifying the representativeness of core collection, appropriate parameters are demanded to ensure genetic diversity [19]. Some sampling strategies and evaluation parameters have now been introduced to develop core collections; however, the optimal sampling strategy and evaluation parameters for each species may be different [20]. Therefore, choosing the optimal sampling strategy and a series of appropriate evaluation parameters are an important aspect of the core collection construction research.

Based on DNA molecular marker data, combining with different sampling strategies, and applying multiple evaluation parameters, core collections have been constructed for many crops, such as for *Phaseolus lunatus* [21], *Oryza sativa* [22], *Zea mays* [23,24], *Glycine max* [25], *Medicago truncatula* [26] and *Cajanus cajan* [27]. Also applied to a lot trees, such as *Cryptomeria japonica* [28], *Dalbergia Odorifera* [29], *Prunus armeniaca* [30], *Malus sieversi* [31], and *Eucalyptus cloeziana* [32]. Nevertheless, development of core collections for trees, especially those kept in the germplasm resource bank, is extremely essential. Because the preservation of this type of tree are usually maintained as living collections in the field, and this has extremely high management costs.

*P*. *sibirica* exhibits high levels of natural variation and variation introduced through selective breeding. Natural variation in *P*. *sibirica* results in part from its self-incompatibility, as well as human-mediated introductions into new regions followed subsequently by local adaptations. Due to the abundant variation and presumed underlying genetic diversity in *P*. *sibirica*, a robust germplasm resource for the species is necessarily very large. Thus, a core collection can aid in reducing the complexity of such a large collection to the benefit of molecular breeding activities and other research. During the past 20 years, the National Forest Germplasm Resources Preservation Repository for *P*. *sibirica* has been operated by the Shenyang Agricultural University in Kazuo County of Liaoning Province, China. In order to better utilize this collection, accelerate the breeding process and add to its scientific and economic value, we sought in this study to establish a core collection of *P*. *sibirica* based on simple sequence repeat (SSR) molecular markers.

## Materials and methods

### Plant materials

From April 2014 to September 2019, 158 accessions of *P*. *sibirica* were collected from China and Russia (total 13 provenances) and were used as the initial collection from which to establish a core collection, including the characteristics of high yield, late-flowering, late-maturing, sweet flesh, double kernels, sweet almond, frost resistance, drought resistance, extreme drought resistance, pink flower, fold flower, pink anther etc. (S1 Table). The 158 accessions were preserved at the National Forest Germplasm Resources Preservation Repository for *P*. *sibirica* by grafting clones (Kazuo County of Liaoning Province, China; Geographical position, 119° 24′54〞E ~ 120° 23′24〞E, 40° 47′12〞N ~ 41° 33′53〞N).

### Experimental site situation

The region of Kazuo country belongs to the low hilly region, the elevation of 300~400 m, the climate of which is continental monsoon. The annual average temperature is 8.7°C, and the annual average precipitation is 491.5 mm. The average sunshine duration is 2807.8 h, and the average frost-free period is 144 d. The soil type of the experimental site is dominated by brown soil. The main woody plant resources include *Prunus sibirica*, *Prunus vulgaris*, *Prunus mandshurica*. The surrounding woody plant resources are mainly *Pinus tabuliformis*, *Robinia pseudoacacia*, *Juglans regia*, *Crataegus pinnatifida*, *Amygdalus Persica*, *Vitis vinifera*, *Salix suchowensis*, etc.

### DNA extraction and primer screening

Fresh young leaves were collected from 1-year-old shoots on sun-facing sides of the 158 individual trees in mid-June and stored these in liquid nitrogen in the field and in a -80°C freezer in the lab prior to DNA extraction. DNA was extracted with DNAsesure Plant Kit (TIANGEN).

Based on the results of Reduced-Representation Genome Sequencing (RRGS) of *P*. *sibirica* in 2014, 600 SSR primers were designed [33] (Beijing SBS Genetech Co., Ltd.). The primers were initially screened using three representative samples of *P*. *sibirica* (#354, #366, #511, respectively). Based on these preliminary screenings [34,35], 30 primer pairs (S2 Table) were selected for downstream analyses based on their polymorphism information content (*PIC*) >0.5.

### PCR amplification and electrophoresis detection

The PCR amplification for SSR analysis were performed in a 20 μL PCR reaction mixture, containing 20 ng of DNA template, 0.15 μg/L of primer concentration, 2.0 mmol/L of Mg^2+^, 1.0 U of Taq polymerase, and 0.25 mmol/L of dNTPs [33]. PCR thermocycling was performed as follows under a hot lid at 105°C: 1) enzyme activation at 94°C for 5 min, 2) 34 cycles of denaturation at 94°C for 30 s, annealing at 55°C for 30 s, and extension at 72°C for 30 s, 3) final extension at 72°C for 5 min, and 4) hold at 4°C until removed and processed [33]. The resulting PCR products were detected using non-denaturing polyacrylamide gel electrophoresis on a 12% gel. and performed electrophoresis for 90 minutes at 220 V. After rinsing, silver staining, and development, the gel was imaged in a gel imaging system (BIO-RAD, USA) (S1 Fig).

### Statistical analysis of SSR data

The sizes of the amplified fragments for each locus were calculated by first comparing to a 100 bp DNA ladder (TIANGEN) in Imagelab 4.0 software and then with manual corrections based on the known size of the SSR repeat unit. Amplified fragments that differed by more than one repeat unit were identified as representing different alleles [36]. SSR dataset was converted different format in DATAtrans 2.0 for use in following analysis software. The observed alleles (N_a_), the number of effective alleles (N_e_), the Shannon’s information index (I), and Nei’s gene diversity (H) were calculated using POPGENE 32 software. Cervus 3.0 was applied to determine polymorphism information content (PIC). Cluster analysis was performed using an Un-weighted Pair Group Method of Arithmetic Average (UPGMA) by NTsys V2.10e software, based on three kinds of genetic distances evaluated by using the Simple Matching genetic similarity coefficient (SM), the Jaccard genetic similarity coefficient (JD) and the Nei & Li genetic similarity coefficient (ND) [37].

### Construction of the core subsets

Based on the cluster analysis, two sampling strategies (Allele preferred strategy, PS; Random strategy, RS) and three genetic distances (SM, JD, and ND) combined with stepwise clustering were used for construction of the core subsets. The (PS) strategy [31] meant that for each pair of accessions clustered in the dendrogram, the individual with more alleles was chosen for the next round of clustering. If the number of alleles was equal, the individual with more rare alleles (allele frequency < 5%) was preferred to choose. If still equal, an individual was randomly chosen. The RS strategy [31] meant that the accession was randomly chosen from each pair of accessions clustered in the dendrogram to enter into the next round of clustering. When the cluster consisted of a single accession, that was the one chosen. The stepwise cluster analyses were repeated in the same way until chosen lineages were reduced to 30%, 25%, 20%, 15% and 10% of the initial collection, at which time the construction of the core subsets was complete.

### Representative evaluation of the core collection

By comparing 4 genetic parameters(N_e_, I, PIC and H), the most optimal ones of core subsets were chosen. A t-test for means was performed to determine if there was a difference in the three parameters between the core collection and the initial collection, and reserved collection (the other accessions except for accessions of core collection). The representativeness of the core collection was also validated by principal coordinates analysis (PCOA) using NTSYS V2.10e, with the distribution of the initial collection and the core collection being plotted by the first principal component score and the second principal component score.

## Results

### Genetic diversity of *P*. *sibirica*

The genetic diversity of the initial collection was analyzed based on 30 pairs of SSR primers (S3 Table). The results of partial primer polyacrylamide were shown in S3 Table. The average of 20 alleles for each pair of primers was detected in each accession, and the range was 9 to 36. The analysis of genetic diversity of the 158 accessions indicated that the average of the observed number of alleles (N_a_), the effective number of alleles (N_e_), Shannon’s information index (I), polymorphic information content (PIC), and Nei’s gene diversity (H) were 22, 8.194, 2.328, 0.847, 0.854, respectively. These parameters clearly indicated that the 158 *P*. *sibirica* germplasms had a high genetic diversity at the molecular level.

### Selection of core subsets

Based on SSR data and UPGMA clustering results, 30 core subsets were constructed using stepwise clustering with two sampling strategies, three genetic distances and five sample sizes (S4 Table). First, the values of N_e_, I, PIC and H of two sampling strategies were compared, to determine which sampling strategy could be used to construct the core collection (S4 Table). When using SM genetic distance to cluster, the indices of N_e_, I, PIC and H of PS strategy were almost larger than the RS, except the values at 30% sampling size. When using JD and ND to cluster, the values of N_e_, I, PIC and H of PS were all higher than the RS strategy. In addition, for PS strategy, the standard deviation (STDV) and coefficient of variability (CV) of N_e_ were 0.370 and 0.043, respectively, and the STDV and CV of I were 0.415 and 0.054, respectively (S4 Table). All of these indices were less than those obtained using RS strategies. Therefore, PS strategies was more appropriate than RS strategies as a sampling strategy to construct a core collection of *P*. *sibirica*.

**Fig 1:**
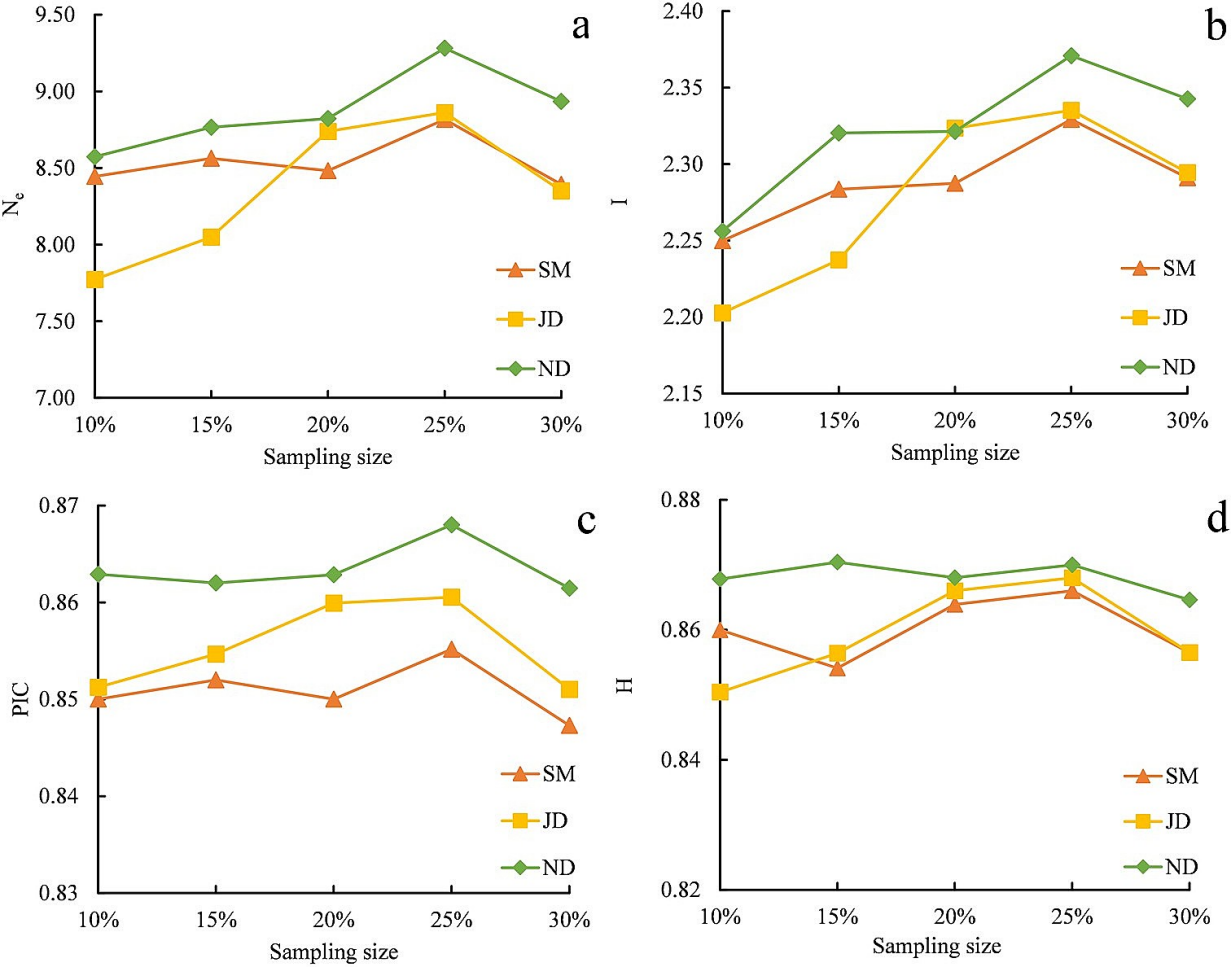
Values of N_e_, I, PIC and H of core subset based on different sample size and different genetic distance with allele preferred strategy. ND: Genetic distance using Nei & Li genetic similarity coefficient, SM: Genetic distance using simple matching coefficient, JD: Genetic distance using Jaccard genetic similarity coefficient, N_E_: Number of effective alleles, I: Shannon’s information index, PIC: Polymorphic information content, H: Nei’s gene diversity.

Further, the values of N_e_, I, PIC and H of the core subsets were also compared constructed using seven different sample sizes (10%, 15%, 20%, 25%, 30%) generated according to the PS strategy among three genetic distances (Fig 1). When using SM, JD and PS strategy, the values of N_e_, I, PIC and H gradually increased and then decreased with the increase of sampling size, reaching the peak values at 25% sampling size, respectively (Fig 1A–1D). Similarly, using ND and PS strategy, the values of N_e_, I, and PIC also reached their maximal value at the 25% sample size (Fig 1A–1C); although H did not significantly change, it also had the maximal value at the 25% sample size(Fig 1D). Thus, the 25% sample size was the most suitable for construction of the core collection using SM, JD and ND, respectively.

**Fig 2:**
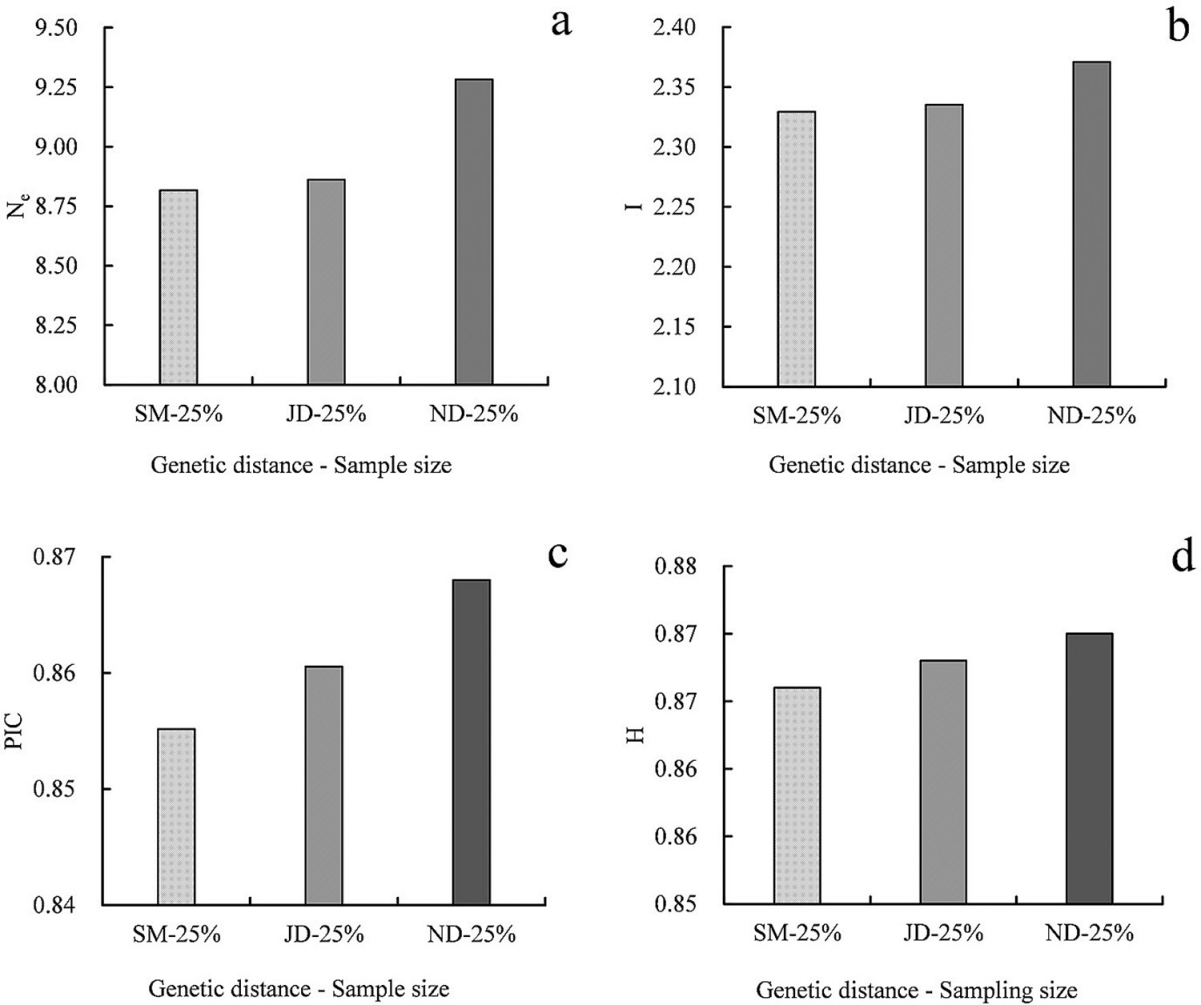
Values of Ne, I, PIC and H of core subsets based on the allele preferred strategy and different genetic distances with a given sample size. N_e_: Number of effective alleles, I: Shannon’s information index, PIC: Polymorphic information content, H: Nei’s gene diversity, ND: Genetic distance using Nei & Li genetic similarity coefficient, SM: Genetic distance using simple matching coefficient, JD: Genetic distance using Jaccard genetic similarity coefficient.

Finally, the values of N_e_, I, PIC and H of core subsets using SM with 25% sampling size, JD with sampling size and ND with 25% sampling size were compared, to determine which genetic distance was the most suitable for establish the core collection (Fig 2A–2D). Obviously, the values of Ne, I, PIC and H of ND-25% were all higher than SM-25% and JD-25%. Therefore, the core subset constructed using the PS strategy with 25% sample size and ND genetic distance had the best representativeness of the initial collection in the 30 core subsets and could be used as the core collection of *P*. *Sibirica*.

### Evaluation and confirmation of core collection

Results of T-test comparing three genetic diversity parameters between the core collection and the initial collection, indicated that the core collection was no significantly different from the initial collection (Table 1). Similarly, the indices except N_e_ of the core collection were no significantly difference with reserved collection (Table 1). The percentages of retention of N_e_, I, PIC and H of the core collection were 113%, 102%, 102% and 103%, which were higher than those of the reserved collection (91%, 96%, 98%, and 99% respectively, Table 2). These results showed that the core collection has higher polymorphism and genetic diversity than the initial collection and reserved collection.

**Table 1:** T-test results of the optimal core collection and initial collection, core collection and reserved collection.

Parameter	Germplasm	Mean	STDV	Pair mean	Pair deviation	T value	Sig.
N_e_	Initial collection	8.194	3.099	-1.088	0.895	-1.215	0.229
	Reserved collection	7.489	2.818	-1.793	0.863	-2.077	0.042*
	Core collection	9.282	3.797				
I	Initial collection	2.328	0.403	-0.043	0.104	-0.412	0.682
	Reserved collection	2.240	0.396	-0.131	0.103	-1.270	0.209
	Core collection	2.371	0.400				
PIC	Initial collection	0.847	0.086	-0.021	0.020	-1.013	0.315
	Reserved collection	0.831	0.095	-0.036	0.217	-1.674	0.099
	Core collection	0.868	0.071				
H	Initial collection	0.854	0.076	-0.016	0.018	-0.906	0.369
	Reserved collection	0.840	0.095	-0.030	0.019	-1.564	0.123
	Core collection	0.870	0.063				

N_e_: Number of effective alleles, I: Shannon’s information index, PIC: Polymorphic information content, H: Nei’s gene diversity.

**Table 2:** Contradistinction of the genetic diversity between initial collection, core collection and reserved collection.

Germplasm	Numbers of germplasm	N_a_	N_e_	I	PIC	H
Initial collection	158	22	8.194	2.328	0.847	0.854
Core collection	40	16	9.282	2.371	0.868	0.870
Percentage of retention	25.3%	73%	113%	102%	102%	103%
Reserved collection	118	18	7.489	2.240	0.831	0.840
Percentage of retention	74.7%	82%	91%	96%	98%	99%

N_e_: Number of effective alleles, I: Shannon’s information index, PIC: Polymorphic information content. H: Nei’s gene diversity.

**Fig 3:**
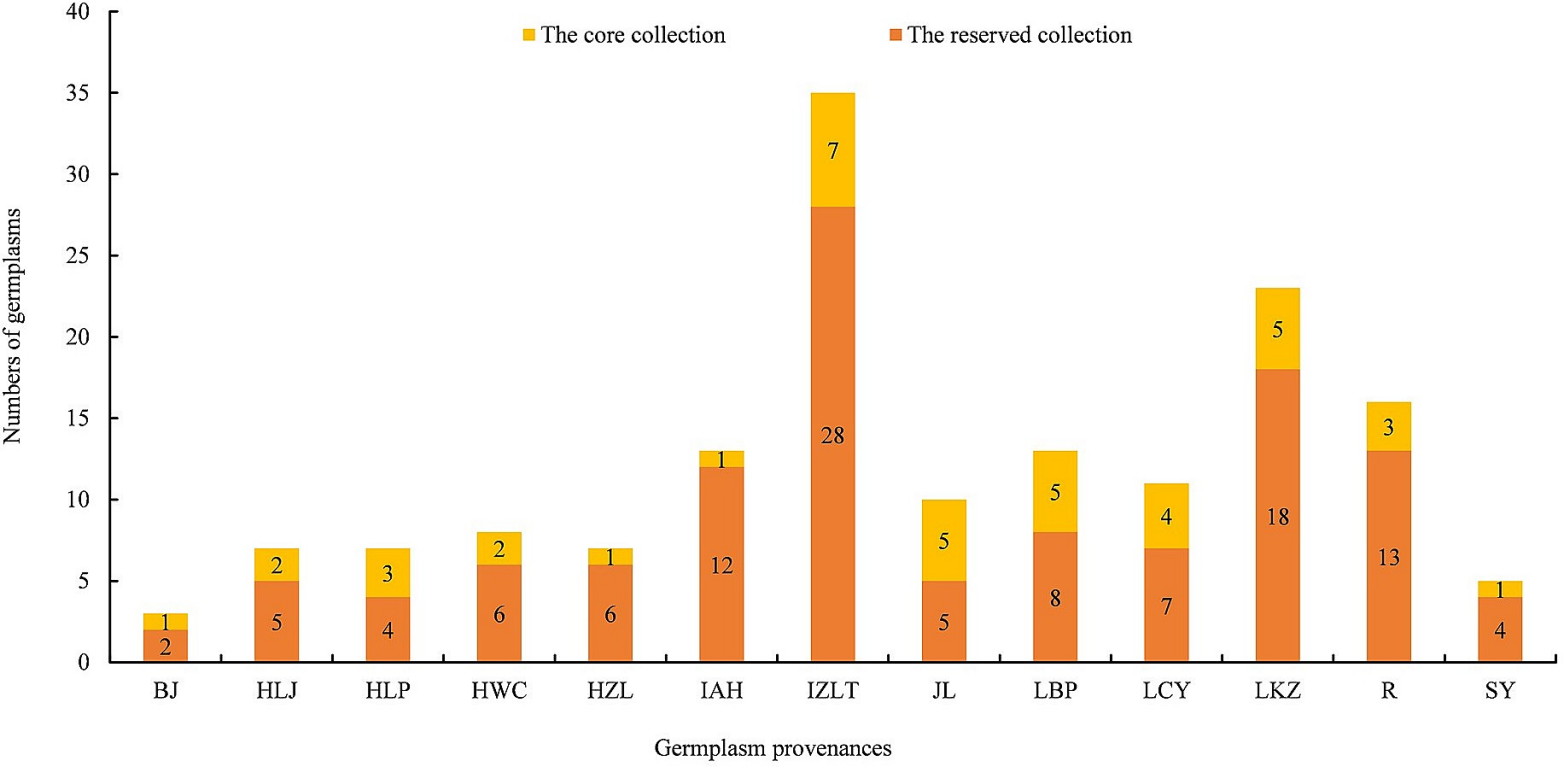
Numbers of core collection and reserved collection of each provenance. The numbers of core collection (yellow) plus the numbers of reserved collection (orange) were equal to the numbers of initial collection. BJ: Beijing; HLJ: Heilongjiang; HLP: Luanping, Hebei; HWC: Weichang, Hebei; HZL: Zhuolu, Hebei; IAH: Aohan, Inner Mongolia; IZLT: Zhalantun, Inner Mongolia; JL: Jilin; LBP: Beipiao, Liaoning; LCY: Chaoyang, Liaoning; LKZ: Kazuo, Liaoning; R: Russia; SY: Yuxian, Shanxi.

**Fig 4:**
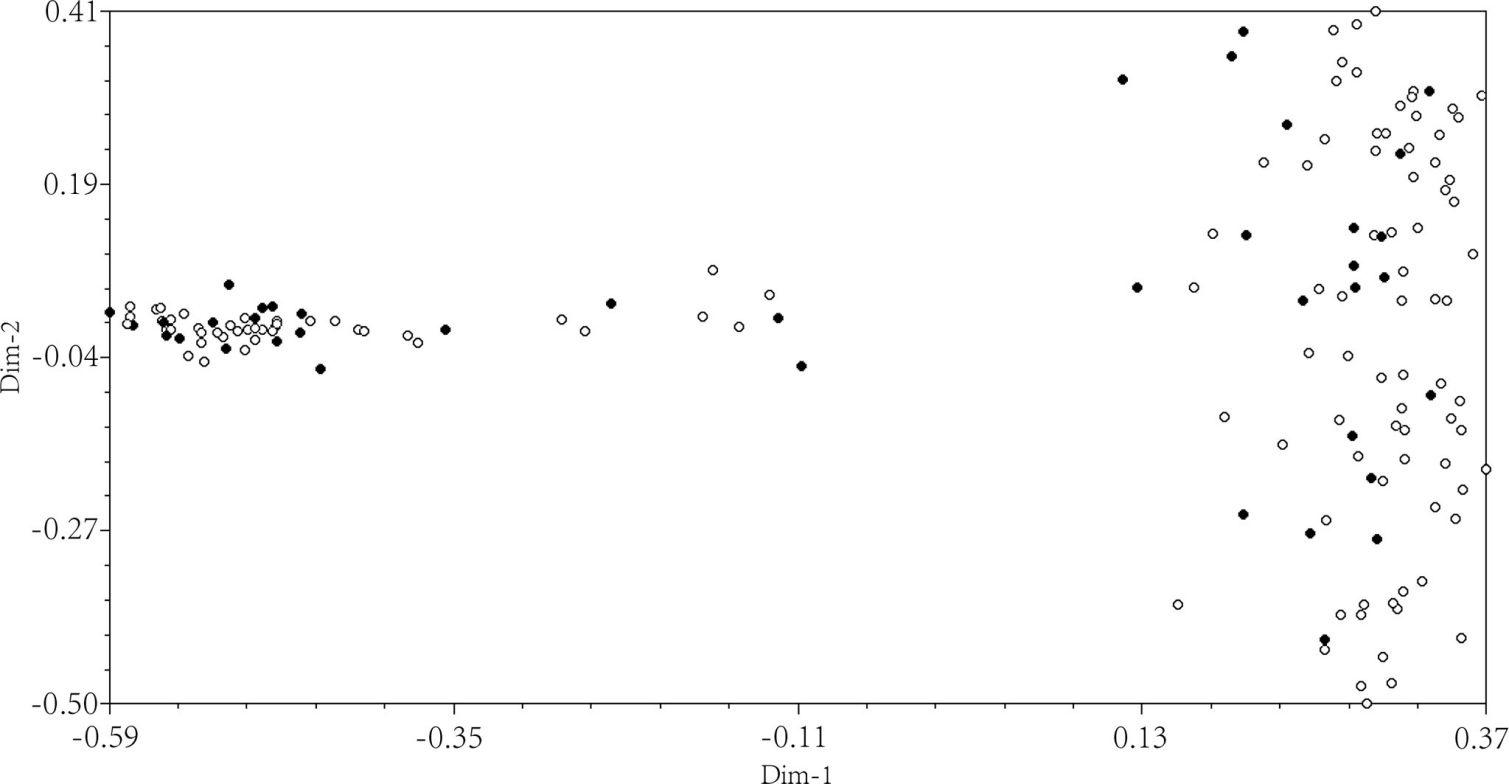
Principal coordinate plot of the core collection constructed using the preferred sampling strategy with 25% sample size and Nei & Li genetic distance and initial collection. The 118 accessions of the reserved collection were represented by open circles and the 40 accessions of the core collection were represented by black circles. All circles represented the 158 of the initial collection. Dim-1 and Dim-2 represented the first principal component score and the second principal component score of 158 accessions.

The geographical distribution of core collection was further analyzed. Each of the 13 provenances all had germplasm selected as core collection. There were 1, 2, 3, 2, 1, 1, 7, 5, 5, 4, 5, 3, 1 accessions were selected into the core collection, respectively, from BJ, HLJ, HLP, HWC, HZL, IAH, IZLT, JL, LBP, LCY, LKZ, R, SY (Fig 3). The results of PCOA showed that the selected samples within the proposed core collection were distributed in a scattered pattern among the 158 total samples (Fig 4). The Fig 4 showed that the distribution pattern of the core collection was very similar to that of the initial collection, and more peripheral individuals were selected, providing further evidence of the representativeness of the proposed core. A total of 158 accessions contained 14 superior and variant types. After selective extraction, the 40 core collections still high yield, double kernel, sweet kernel, cold resistance, late flower, sweet meat, drought tolerance, extreme drought tolerance, late maturity, late-flowering (S1 Table). These results indicated the representativeness of the core collection constructed by allele preferred strategy combined with 25% sampling size. Thus, priority should be given to research and utilization of accessions represented within this newly established core collection of *P*. *sibirica*.

## Discussion

### Molecular data of constructing a core collection

At present, phenotypic traits and molecular markers are often used to construct core collections [5]. Phenotypic traits result from underlying genotypes but are affected by compound interactions between the genotype and environment. Therefore, phenotypic traits are not always good proxies for genetic diversity and cannot fully reflect the genetic diversity of an entire germplasm collection. Moreover, for trees, years of continuous investigation are often needed to obtain reliable phenotypic data [14]. Molecular markers, which are developed based on DNA polymorphisms, represent a direct method of measuring genetic diversity and are rarely affected by environmental interactions. Therefore, they are more suitable for constructing a core collection and evaluating genetic diversity than phenotypic traits [15]. Molecular markers are divided into dominant and codominant types. SSRs are a codominant type of marker and consequently can provide richer allelic information than dominate markers [38]. Therefore, 30 SSR primers with high polymorphism were used to obtain 40 accessions of *P*. *sibirica* as a core collection to represent the initial collection. The successful establishment of core collection was of great significance to the further research and utilization of *P*. *Sibirica*. germplasm resources.

### Methods for constructing the core collection

Sampling strategy is critical to construction of a core collection. Since the concept of core collection was proposed, many scholars have given different suggestions on its construction methods, such as the method of maximizing the number of alleles [39], and the clustering method based on genetic distance [40], rare allele priority method (PS) [31], etc. So far, rare allele priority method is one of the most common methods. It has been used in some forest tree species, for instance *Ficus carica L*. [41], *Castanea mollissima* [42], *Armeniaca vulgaris* Lam [43] and so on. Ideally, a core collection should represent the genetic diversity of the entire germplasm resource as comprehensively as possible, and the genetic diversity of the entire germplasm resource depends largely on the number of alleles and allele frequencies at all loci [44]. Therefore, rare alleles (allele frequency < 5%) should preferentially be selected when selecting among germplasm to develop a core collection. As a result, it is widely acknowledged that PS strategy is likely better than a RS strategy for most species [31,43,45]. Consistent with previous results, in this study, we found that PS yielded higher values for N_e_, I, PIC and H than the RS in all candidate core collections. Therefore, PS is a better sampling strategy for constructing the core collection of *P*. *sibirica*.

It is important to confirm a reasonable sampling percentage during core collection construction. Brown proposed that an ideal core collection size should be about 10% of the total collection, which maintained over 70% of the alleles in the whole collection with 95% certainty [9]. Yoneazwa et al. suggested that the optimal proportion was 20–30% [46]. However, The sample percentage of a typical core collection is generally between 5% and 40% of the germplasm resource [4,47]. Generally, the appropriate sampling percentage should be determined according to the characteristics of different germplasm populations; the initial germplasm population is small, it is most suitable to choose a large sampling percentage, while the initial germplasm population is large, it is most suitable to choose a larger sampling percentage [29,37]. Gomes et al. analyzed the genetic diversity of 153 lima bean breeding lines and obtained a core collection of 34 lines, accounting for 22% of the initial collection [21]. Hu et al. analyzed the genetic diversity of 612 accessions of *Cucumis melo* L. and obtained a core collection of 118 accessions, accounting for 19.4% of the initial collection [48]. Mongkolporn et al. analyzed the genetic diversity of 230 *Capsicum* spp. and obtained a core collection of 28 accessions, accounting for 12% of the initial collection [49]. These results indicated that the sampling percentage should depend on the quantity and genetic diversity of the germplasm population. According to the germplasm population size in this study, five sampling percentages (10%, 15%, 20%, 25%, 30%) were set to establish the core collection. By comparing values of N_e_, I, PIC and H among the core collections constructed with the 5 sample sizes using the PS strategy and the same genetic distance, the optimal proportion was found to be 25% for SM, JD and ND, respectively.

Genetic distance is a measure of the genetic difference between species in a population. The genetic distance measures, SM, JD, and ND, are commonly used the clustering of molecular markers such as into dendrograms and are known to produce different results [31]. So, genetic distance will affect the results of clustering and the construction of a core collection [47]. In this study, the Ne, I, PIC and H values of the core collection constructed with 25% sample size with ND genetic distance were higher than those of other methods. Therefore, the core collection constructed by the PS strategy using 25% sample size and ND genetic distance was considered to be the core collection of *P*. *sibirica*.

### Evaluation of the proposed core collection

Representativeness is the most important property of core collection. Commonly used core collection genetic diversity evaluation parameters are: Number of alleles, Shannon diversity index and Nei’s gene diversity index. The number of alleles was considered the most relevant indicator [50,51]. Wang et al. considered that Shannon diversity index, Polymorphic information content and Simpson diversity index were important parameters for evaluating the representativeness of core collection when studying the evaluation parameters of rice core germplasm [47]. Some scholars have also used parameters such as the percentage of polymorphic loci, the number of observed alleles, and the number of effective alleles to evaluate the genetic diversity of core collections [47,52]. Based on previous research results, this study selected 4 parameters (N_a_, N_e_, I, PIC, H) and their retention ratios, combined with the t-test and principal coordinate analysis (PCO), to verify and confirm the optimal core collection, and the effect is better. The retention ratio was the percentage of each genetic parameter of the core collection to each genetic parameter of the initial collection [53]. In this study, the retention ratios of 4 parameters (Ne, I, PIC, H) were greater than 100%, which was mainly caused by changes in the number of samples and allele frequencies in the population. These genetic parameters were estimated by different methods based on the allele frequency, and were used to measure the genetic redundancy in the diversity of different populations (initial collection, core collection, and reserved collection). The construction process of core collection is a process of reducing the frequency of alleles and increasing the proportion of rare alleles. In the process of removing genetic redundancy, the irregular increase and decrease of the frequency of each allele can easily lead to the retention rate of the corresponding genetic diversity parameter being greater than 100%. This phenomenon was common in the core collections of *Armeniaca vulgaris* Lam. [43], and *Eucommia ulmoides* [53], exist. According to the allele retention ratio must be greater than 70%, the larger the other genetic parameters, the better the evaluation criteria [27,54]. In the case that the retention ratios of N_a_ were 73%, the core collection constructed in this study has better genetic parameters and meet the requirements of the core collection, indicating that the effect of retaining the genetic diversity of the initial collection was better.

### Characteristics of germplasms

Siberian apricot breeding process hinge on the abundant germplasm resources, and understanding the characteristics of resources is the prerequisite for effective utilization of apricot germplasm resources. In the past 20 years, 158 Siberian apricot germplasm resources collected by our research group were characterized and evaluated. The germplasm resources of Siberian apricot were rich in characteristics, including high yield, frost resistance, drought resistance, bent branch, late-maturing, late-flowering, fold flower etc. Among those characteristics, 70.25% germplasms had the potential of high yield. Approximately 8% of the germplasm has the characteristics of late flowering or barren tolerance, which makes it adapt to a more complex ecological environment. A small amount of Siberian apricot also has attractive characteristics on branches, petals, fruits, and almond and germplasm with these characteristics could be very useful in food, ornamental and other aspects. All in all, Siberian apricot variation is abundant. We used these characteristics information to evaluate the core collection based on SSR, and the results showed that the core collection contains most of the characteristic types.

## Conclusions

In this study, we tested combinations of sampling strategies, measures of genetic diversity, and sample reduction techniques to find the one most suited to this species using SSR markers data. Based on these tests, we established a core collection based on an allele preferred sampling strategy, the Nei & Li genetic distance, and 25% sampling from the complete resource. The new core collection comprises of 40 *P*. *sibirica* from 13 geographical regions and could represent the genetic diversity of the complete germplasm collection. In the next step, we will focus on the investigation of phenotypic traits of core collection to provide more valuable information for the development and utilization of germplasm resources of Siberian apricot, which will lead to better utilization of germplasm for Siberian apricot breeding programs. In addition, the core collection is further modification by continuously adding new germplasm resources, which will give interesting insights about the representation of unknown Siberian apricot diversity.

## Supporting information

S1 Fig The polyacrylamide gel electrophoresis of PCR products amplified by SSR primers. (PDF)

S1 Table The *Prunus sibirica* germplasm resources for test materials. (DOCX)

S2 Table The information of 30 SSR markers. (DOCX)

S3 Table Diversity index at 30 SSR loci in *Prunus sibirica*. (DOCX)

S4 Table The genetic diversity of the initial collection and 30 core subsets. (DOCX)
