# Supplementary material for: Selection of a core collection of Prunus sibirica L. germplasm by a stepwise clustering method using simple sequence repeat markers
Source: PLoS One. 2021 Nov 19;16(11):e0260097. doi: 10.1371/journal.pone.0260097 (PMC8604298; doi:10.1371/journal.pone.0260097)
Supplement: S1 Fig — (PDF) [file pone.0260097.s001.pdf]

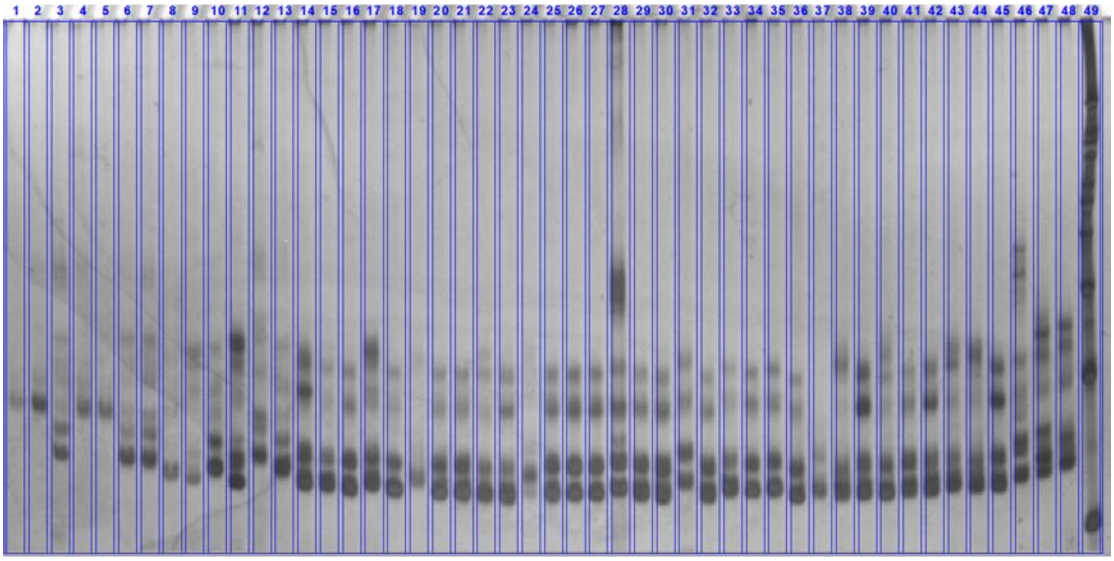
1 2 3 4 5 6 7 8 9 10 11 12 13 14 15 16 17 18 19 20 21 22 23 24 25 26 27 28 29 30 31 32 33 34 35 36 37 38 39 40 41 42 43 44 45 46 47 48 49

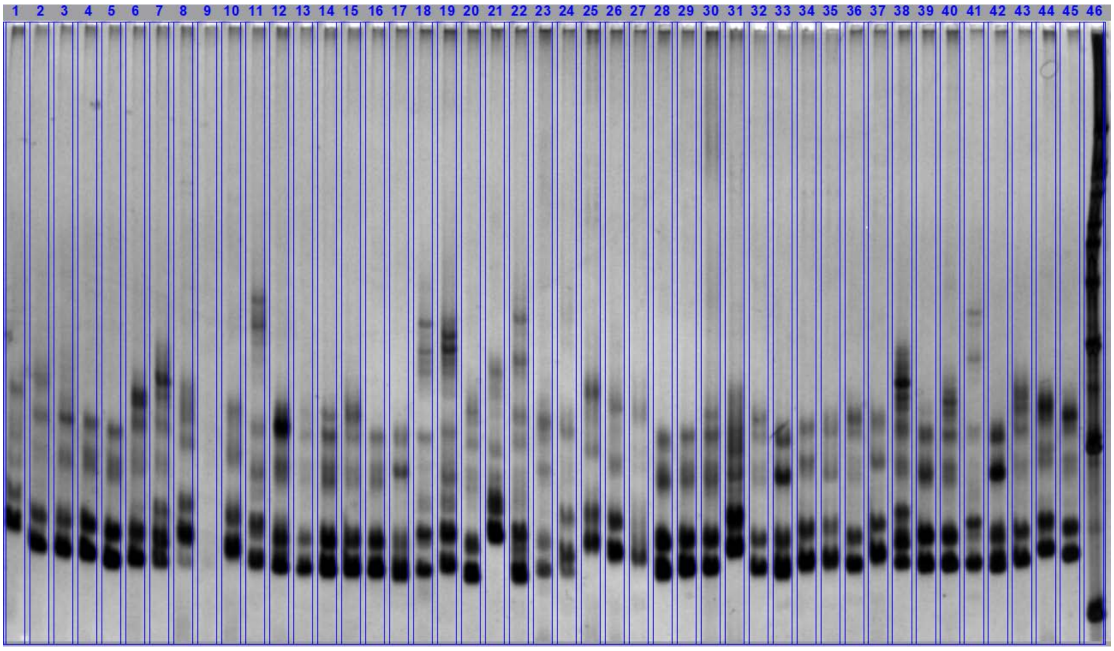
1 2 3 4 5 6 7 8 9 10 11 12 13 14 15 16 17 18 19 20 21 22 23 24 25 26 27 28 29 30 31 32 33 34 35 36 37 38 39 40 41 42 43 44 45 46

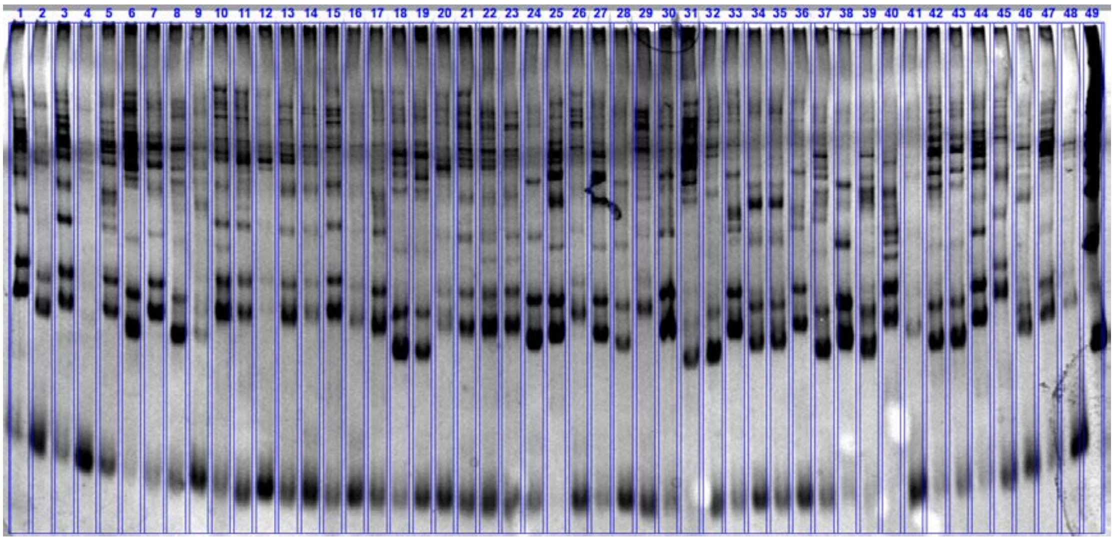
1 2 3 4 5 6 7 8 9 10 11 12 13 14 15 16 17 18 19 20 21 22 23 24 25 26 27 28 29 30 31 32 33 34 35 36 37 38 39 40 41 42 43 44 45 46 47 48 49

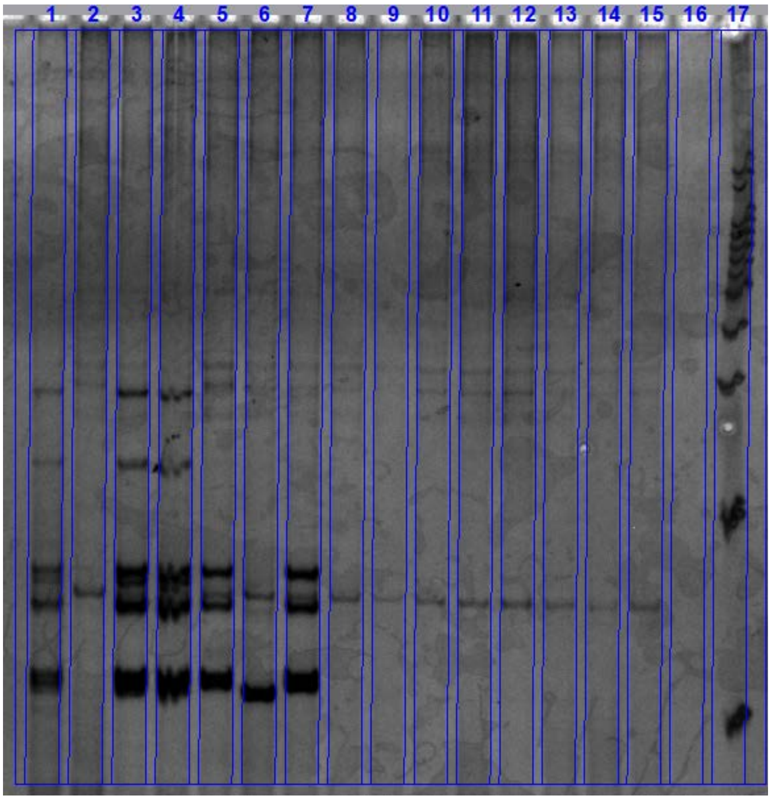

The gel image of primer L7. The accessions from left to right in each gel image were 1, 2, 3, 4, 5, 6, 7, 8, 9, 10, 11, 13, 14, 16, 18, 19, 20, 21, 22, 23, 24, 25, 26, 27, 28, 29, 30, 31, 32, 33, 34, 35, 36, 37, 38, 39, 40, 41, 42, 43, 44, 45, 46, 47, 48, 49, 53, 55, 62, 71, 72, 81, 87, 89, 94, 99, 322, 324, 328, 329, 332, 334, 339, 341, 345, 350, 354, 358, 366,367, 368, 375, 381, 401, 404, 405, 406, 407, 408, 409, 421, 442, 443, 449, 455, 457, 459, 460, 462, 463, 464, 501, 502, 503, 504, 506, 507, 508, 509, 510, 511, 513, 516, 517, 518, 544, 558, 591, 592, 593, 594, 595, 596, 621, 622, 624, 626, 628, 754, 759, 771, 1001, 1002, 1003, 1004, 1005, 1006, BF, BX, BY01, BY02, BY03, JTY, KT, HW01, HW04, HW10, HW10, HW14, HW18, HW19, HW20, HL01, HL06, HL07, HL09, HL12, HL13, HL14, HZ03, HZ07, HZ09, HZ10, HZ11, HZ13, HZ14, XB.

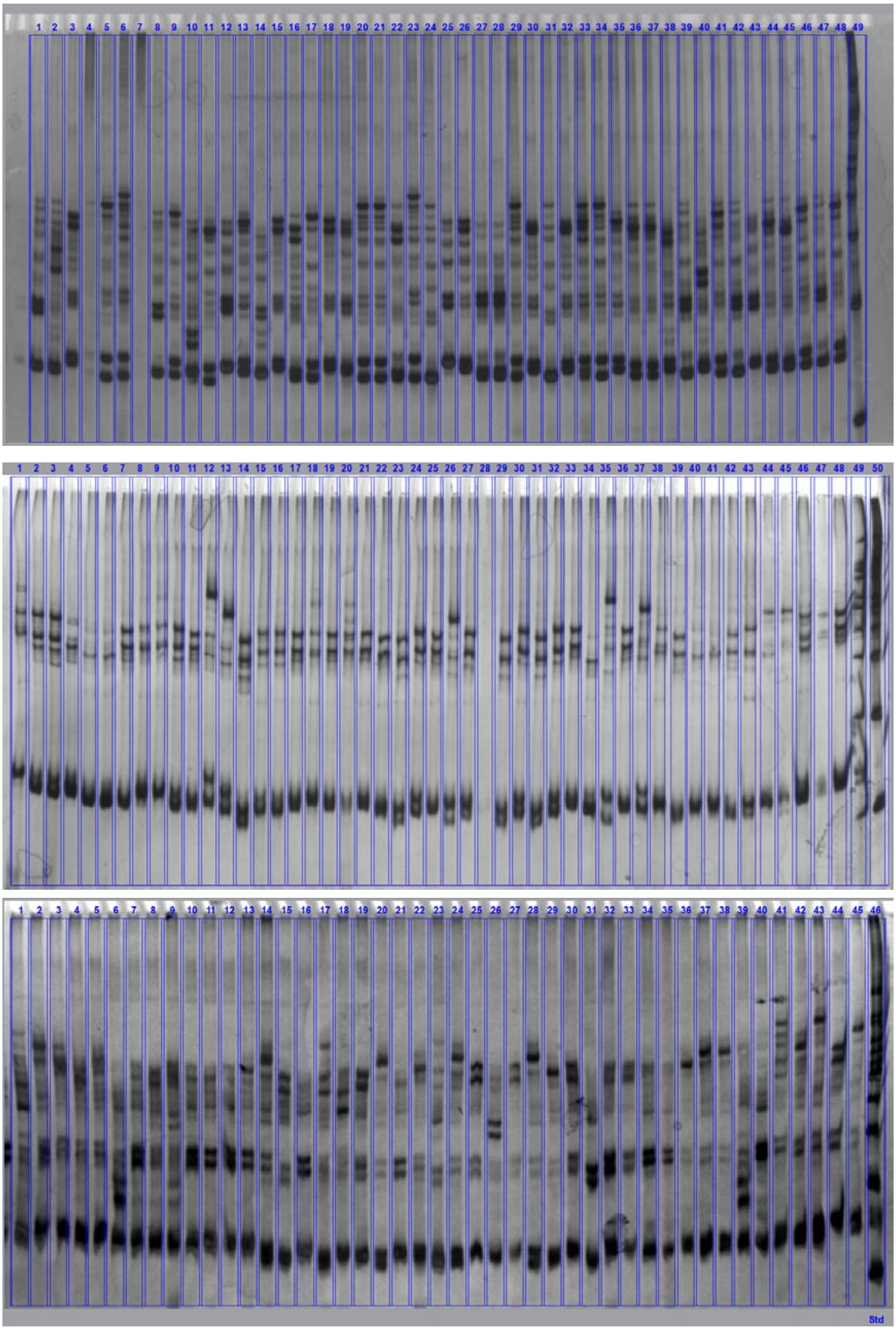
1 2 3 4 5 6 7 8 9 10 11 12 13 14 15 16 17 18 19 20 21 22 23 24 25 26 27 28 29 30 31 32 33 34 35 36 37 38 39 40 41 42 43 44 45 46 47 48 49
1 2 3 4 5 6 7 8 9 10 11 12 13 14 15 16 17 18 19 20 21 22 23 24 25 26 27 28 29 30 31 32 33 34 35 36 37 38 39 40 41 42 43 44 45 46 47 48 49 50
1 2 3 4 5 6 7 8 9 10 11 12 13 14 15 16 17 18 19 20 21 22 23 24 25 26 27 28 29 30 31 32 33 34 35 36 37 38 39 40 41 42 43 44 45 46
Std

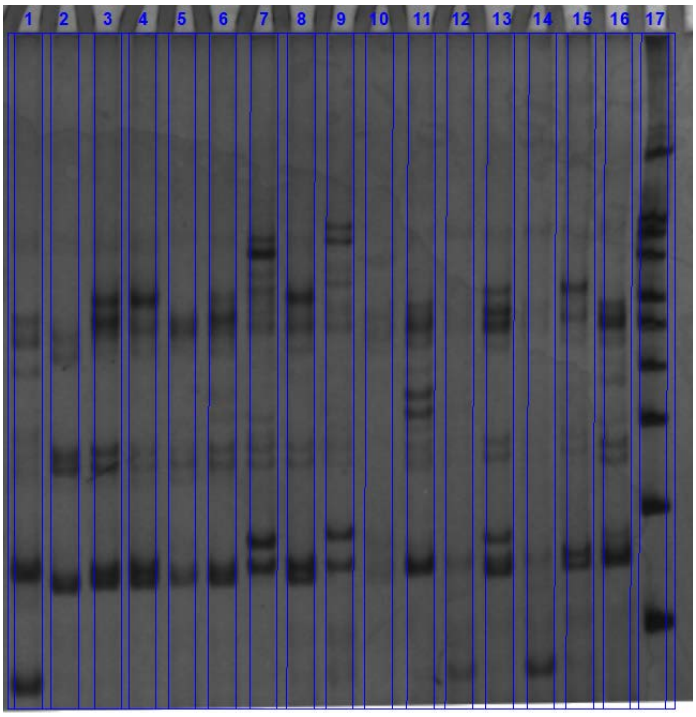

The gel images of primer L23. The accessions from left to right in each gel image were 1, 2, 3, 4, 5, 6, 7, 8, 9, 10, 11, 13, 14, 16, 18, 19, 20, 21, 22, 23, 24, 25, 26, 27, 28, 29, 30, 31, 32, 33, 34, 35, 36, 37, 38, 39, 40, 41, 42, 43, 44, 45, 46, 47, 48, 49, 53, 55, 62, 71, 72, 81, 87, 89, 94, 99, 322, 324, 328, 329, 332, 334, 339, 341, 345, 350, 354, 358, 366,367, 368, 375, 381, 401, 404, 405, 406, 407, 408, 409, 421, 442, 443, 449, 455, 457, 459, 460, 462, 463, 464, 501, 502, 503, 504, 506, 507, 508, 509, 510, 511, 513, 516, 517, 518, 544, 558, 591, 592, 593, 594, 595, 596, 621, 622, 624, 626, 628, 754, 759, 771, 1001, 1002, 1003, 1004, 1005, 1006, BF, BX, BY01, BY02, BY03, JTY, KT, HW01, HW04, HW10, HW10, HW14, HW18, HW19, HW20, HL01, HL06, HL07, HL09, HL12, HL13, HL14, HZ03, HZ07, HZ09, HZ10, HZ11, HZ13, HZ14, XB.

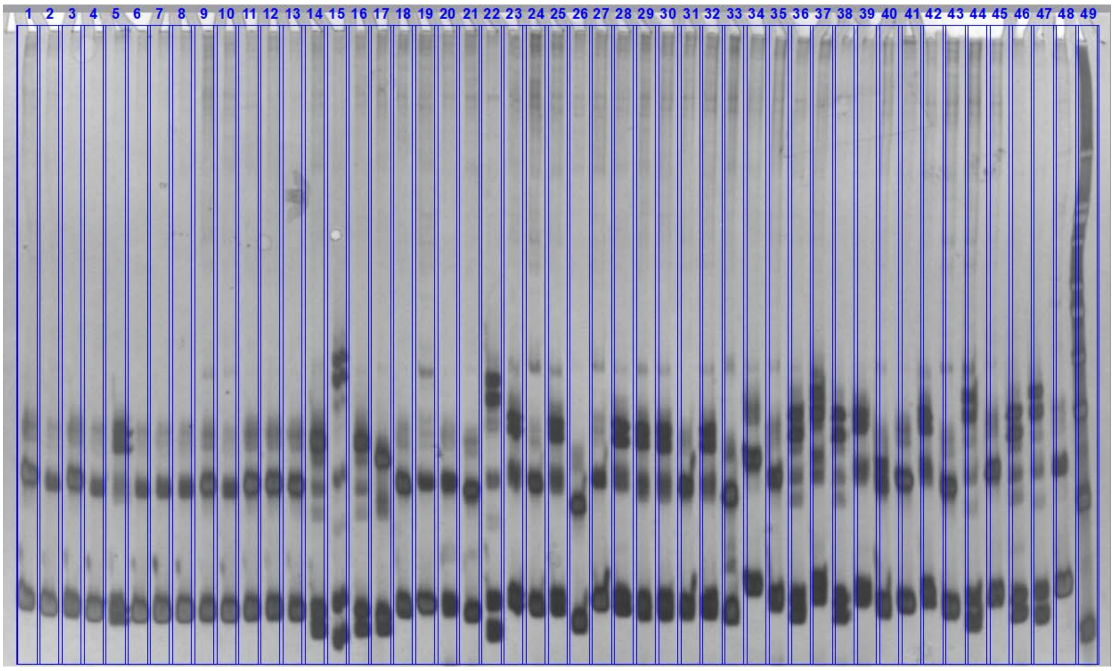
1 2 3 4 5 6 7 8 9 10 11 12 13 14 15 16 17 18 19 20 21 22 23 24 25 26 27 28 29 30 31 32 33 34 35 36 37 38 39 40 41 42 43 44 45 46 47 48 49

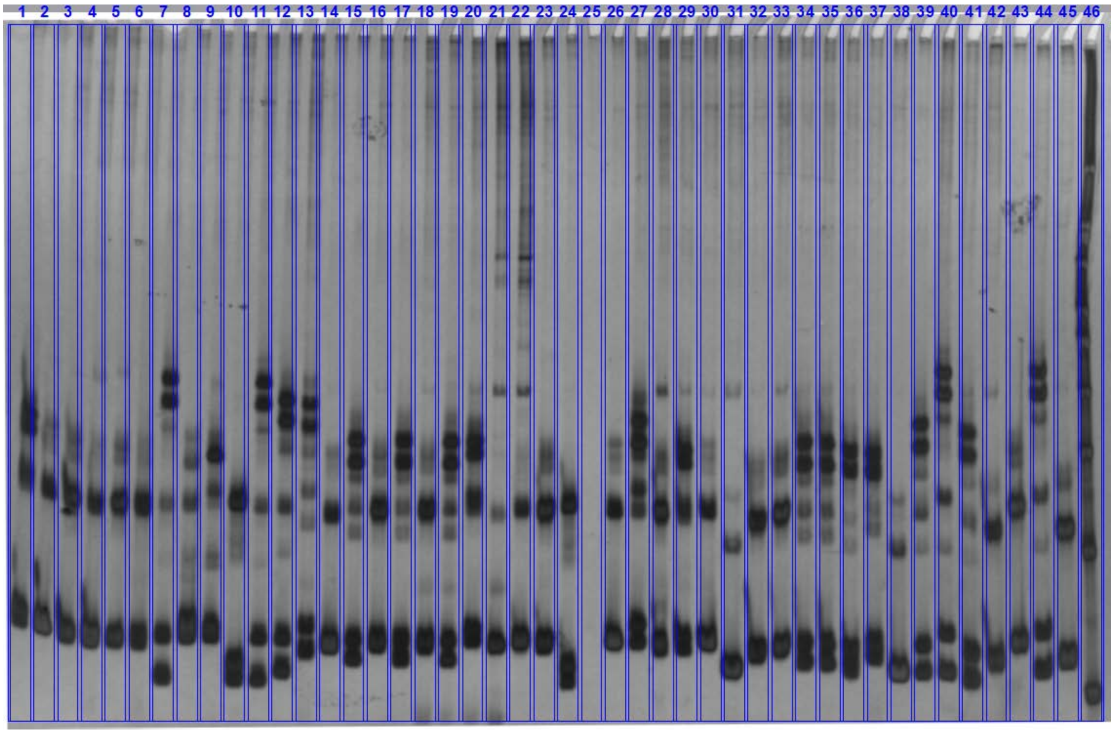
1 2 3 4 5 6 7 8 9 10 11 12 13 14 15 16 17 18 19 20 21 22 23 24 25 26 27 28 29 30 31 32 33 34 35 36 37 38 39 40 41 42 43 44 45 46

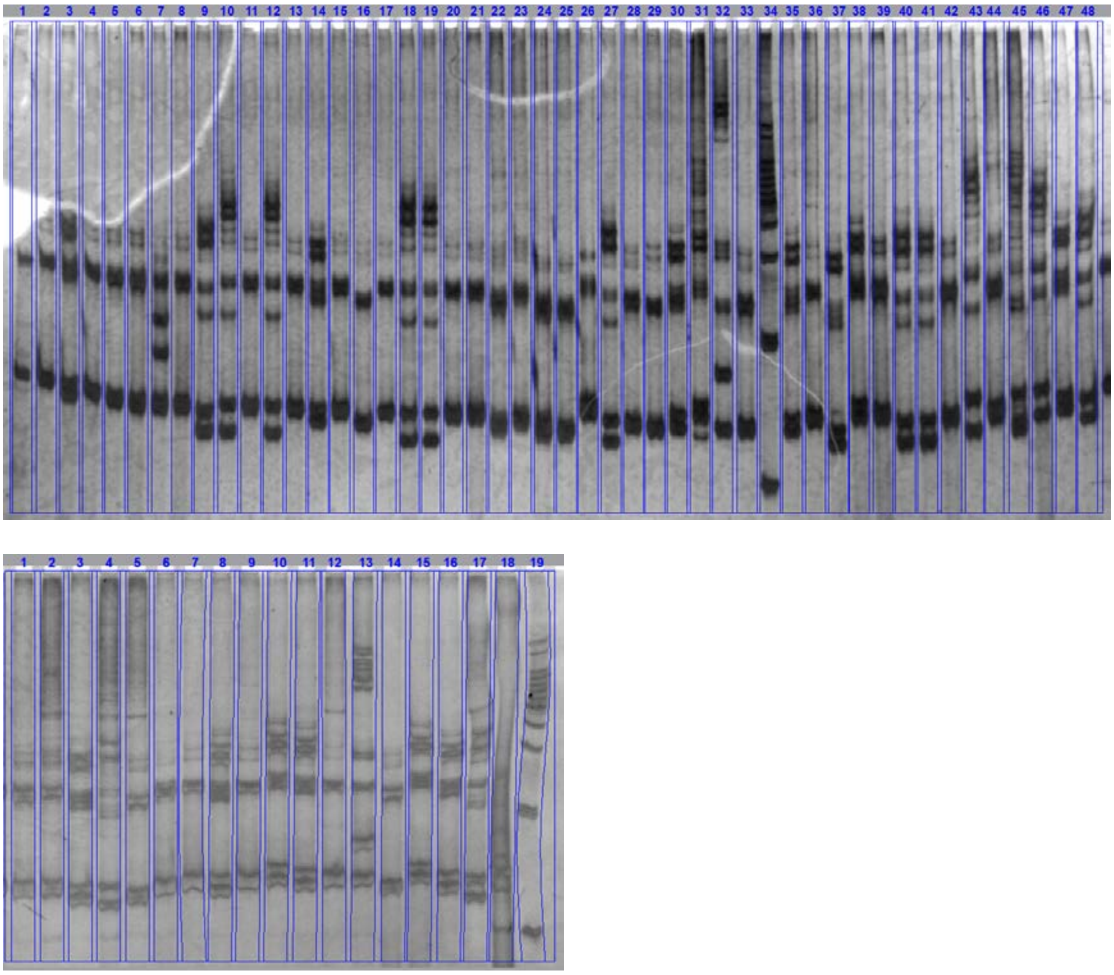

The gel images of primer L25. The accessions from left to right in each gel image were 1, 2, 3, 4, 5, 6, 7, 8, 9, 10, 11, 13, 14, 16, 18, 19, 20, 21, 22, 23, 24, 25, 26, 27, 28, 29, 30, 31, 32, 33, 34, 35, 36, 37, 38, 39, 40, 41, 42, 43, 44, 45, 46, 47, 48, 49, 53, 55, 62, 71, 72, 81, 87, 89, 94, 99, 322, 324, 328, 329, 332, 334, 339, 341, 345, 350, 354, 358, 366,367, 368, 375, 381, 401, 404, 405, 406, 407, 408, 409, 421, 442, 443, 449, 455, 457, 459, 460, 462, 463, 464, 501, 502, 503, 504, 506, 507, 508, 509, 510, 511, 513, 516, 517, 518, 544, 558, 591, 592, 593, 594, 595, 596, 621, 622, 624, 626, 628, 754, 759, 771, 1001, 1002, 1003, 1004, 1005, 1006, BF, BX, BY01, BY02, BY03, JTY, KT, HW01, HW04, HW10, HW10, HW14, HW18, HW19, HW20, HL01, HL06, HL07, HL09, HL12, HL13, HL14, HZ03, HZ07, HZ09, HZ10, HZ11, HZ13, HZ14, XB.

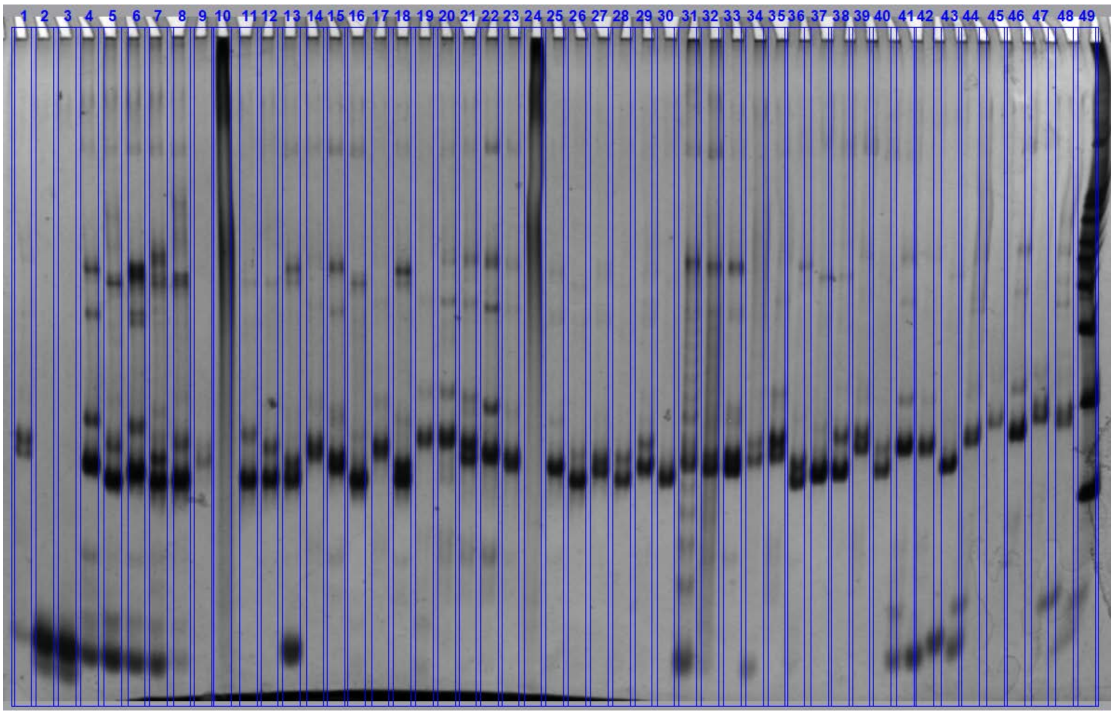
1 2 3 4 5 6 7 8 9 10 11 12 13 14 15 16 17 18 19 20 21 22 23 24 25 26 27 28 29 30 31 32 33 34 35 36 37 38 39 40 41 42 43 44 45 46 47 48 49

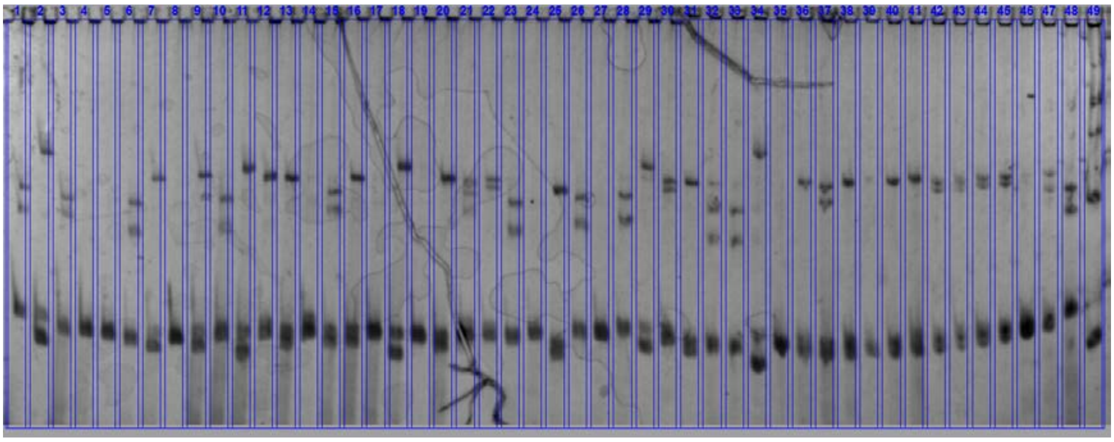
1 2 3 4 5 6 7 8 9 10 11 12 13 14 15 16 17 18 19 20 21 22 23 24 25 26 27 28 29 30 31 32 33 34 35 36 37 38 39 40 41 42 43 44 45 46 47 48 49

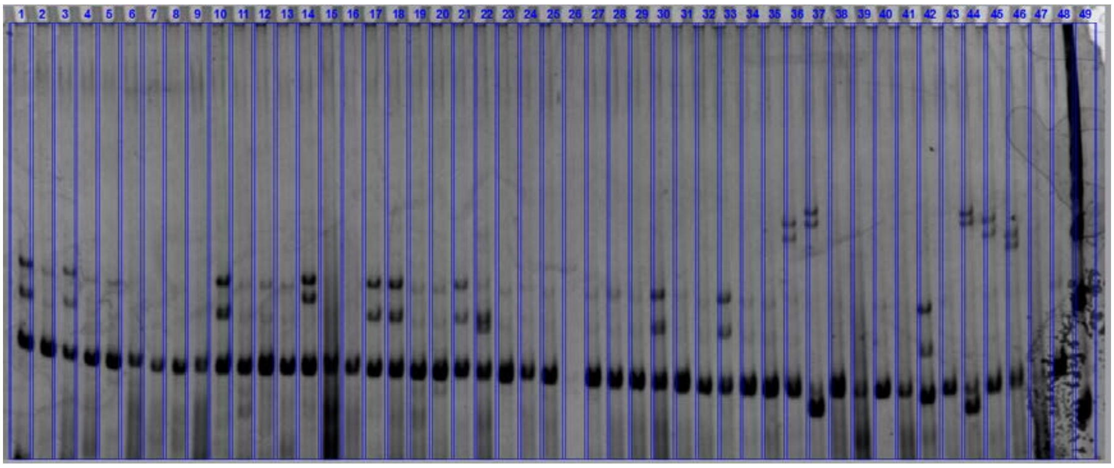

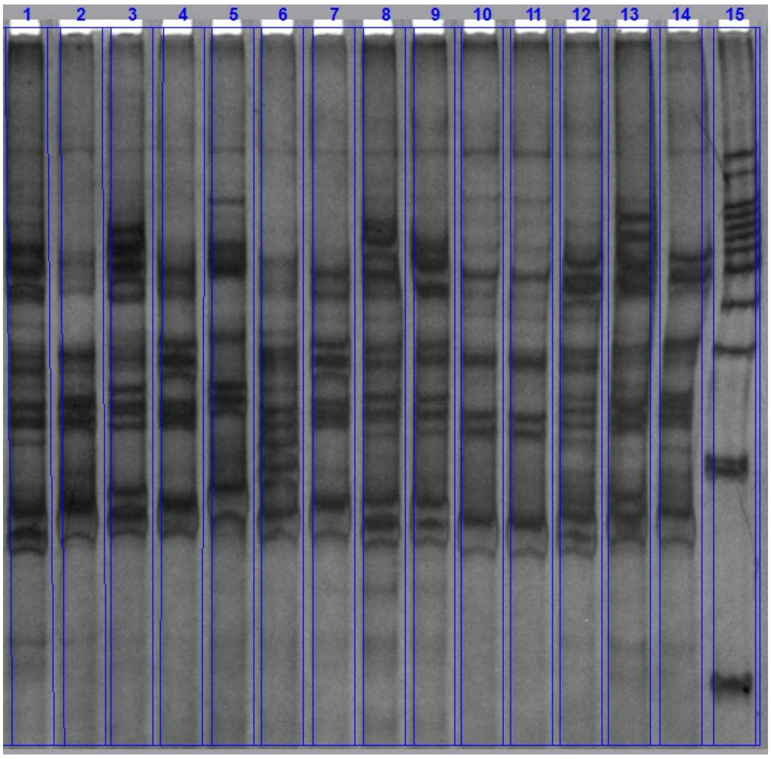

The gel images of primer L46. The accessions from left to right in each gel image were 1, 2, 3, 4, 5, 6, 7, 8, 9, 10, 11, 13, 14, 16, 18, 19, 20, 21, 22, 23, 24, 25, 26, 27, 28, 29, 30, 31, 32, 33, 34, 35, 36, 37, 38, 39, 40, 41, 42, 43, 44, 45, 46, 47, 48, 49, 53, 55, 62, 71, 72, 81, 87, 89, 94, 99, 322, 324, 328, 329, 332, 334, 339, 341, 345, 350, 354, 358, 366,367, 368, 375, 381, 401, 404, 405, 406, 407, 408, 409, 421, 442, 443, 449, 455, 457, 459, 460, 462, 463, 464, 501, 502, 503, 504, 506, 507, 508, 509, 510, 511, 513, 516, 517, 518, 544, 558, 591, 592, 593, 594, 595, 596, 621, 622, 624, 626, 628, 754, 759, 771, 1001, 1002, 1003, 1004, 1005, 1006, BF, BX, BY01, BY02, BY03, JTY, KT, HW01, HW04, HW10, HW10, HW14, HW18, HW19, HW20, HL01, HL06, HL07, HL09, HL12, HL13, HL14, HZ03, HZ07, HZ09, HZ10, HZ11, HZ13, HZ14, XB.

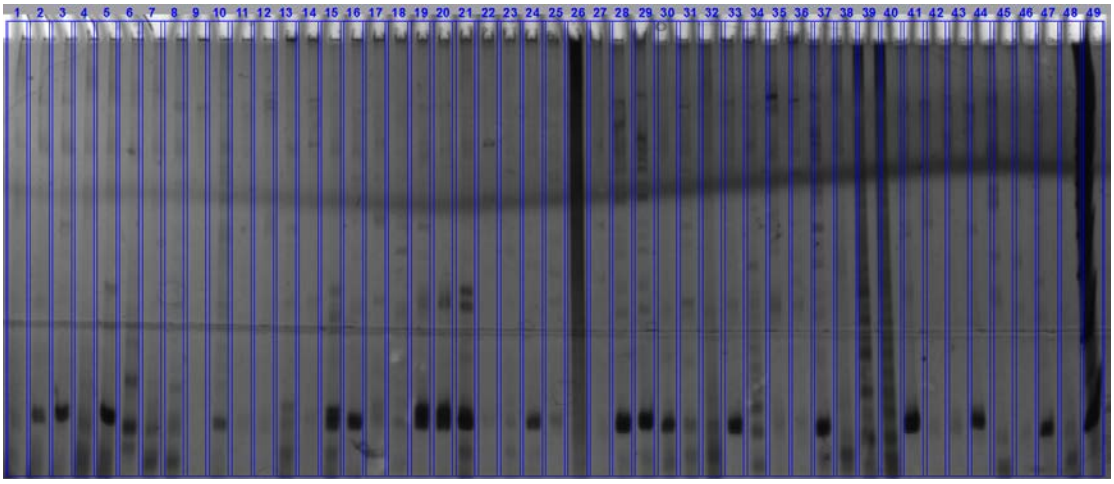
1 2 3 4 5 6 7 8 9 10 11 12 13 14 15 16 17 18 19 20 21 22 23 24 25 26 27 28 29 30 31 32 33 34 35 36 37 38 39 40 41 42 43 44 45 46 47 48 49

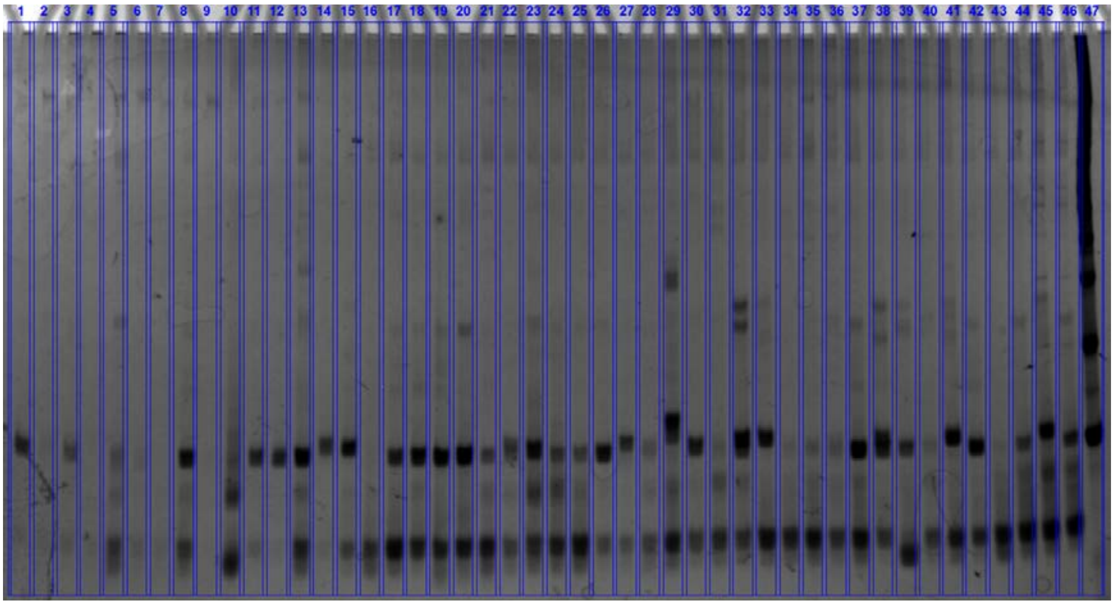
1 2 3 4 5 6 7 8 9 10 11 12 13 14 15 16 17 18 19 20 21 22 23 24 25 26 27 28 29 30 31 32 33 34 35 36 37 38 39 40 41 42 43 44 45 46 47

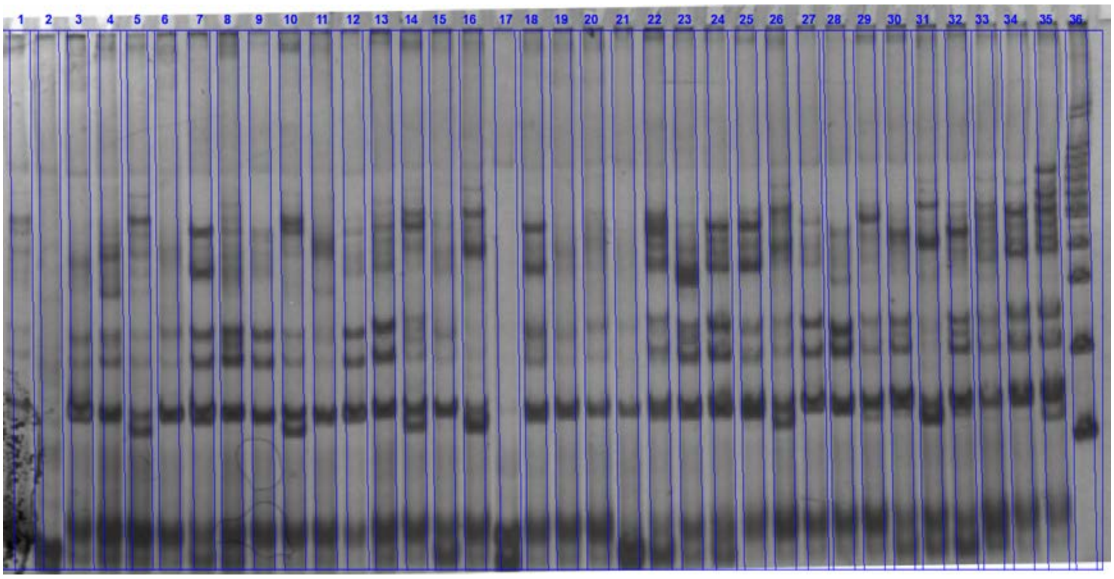
1 2 3 4 5 6 7 8 9 10 11 12 13 14 15 16 17 18 19 20 21 22 23 24 25 26 27 28 29 30 31 32 33 34 35 36

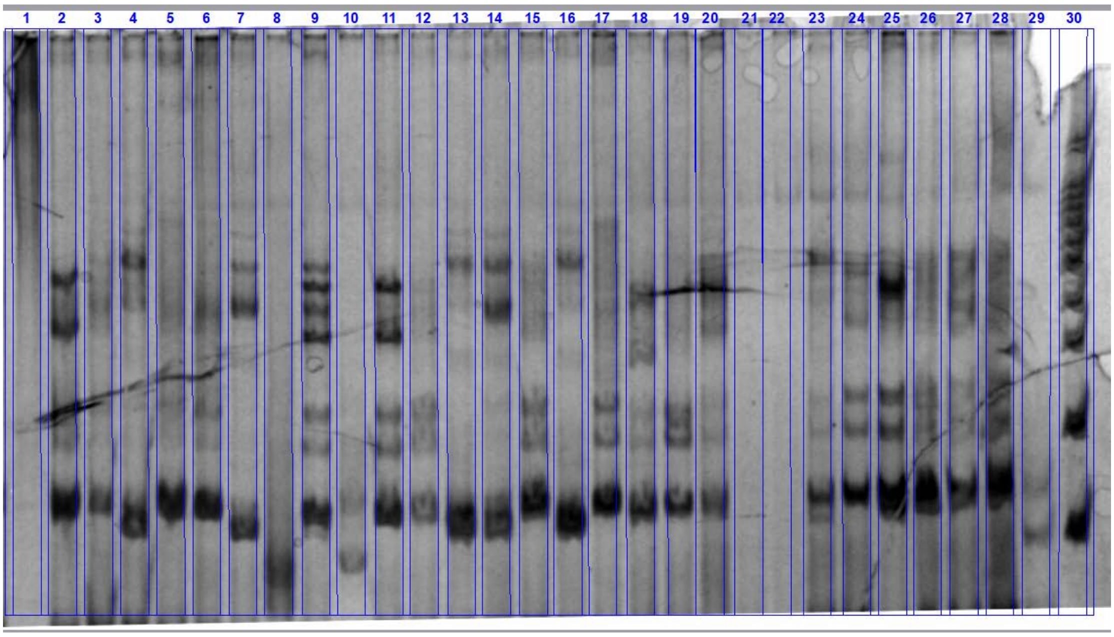

The gel images of primer L49. The accessions from left to right in each gel image were 1, 2, 3, 4, 5, 6, 7, 8, 9, 10, 11, 13, 14, 16, 18, 19, 20, 21, 22, 23, 24, 25, 26, 27, 28, 29, 30, 31, 32, 33, 34, 35, 36, 37, 38, 39, 40, 41, 42, 43, 44, 45, 46, 47, 48, 49, 53, 55, 62, 71, 72, 81, 87, 89, 94, 99, 322, 324, 328, 329, 332, 334, 339, 341, 345, 350, 354, 358, 366,367, 368, 375, 381, 401, 404, 405, 406, 407, 408, 409, 421, 442, 443, 449, 455, 457, 459, 460, 462, 463, 464, 501, 502, 503, 504, 506, 507, 508, 509, 510, 511, 513, 516, 517, 518, 544, 558, 591, 592, 593, 594, 595, 596, 621, 622, 624, 626, 628, 754, 759, 771, 1001, 1002, 1003, 1004, 1005, 1006, BF, BX, BY01, BY02, BY03, JTY, KT, HW01, HW04, HW10, HW10, HW14, HW18, HW19, HW20, HL01, HL06, HL07, HL09, HL12, HL13, HL14, HZ03, HZ07, HZ09, HZ10, HZ11, HZ13, HZ14, XB.

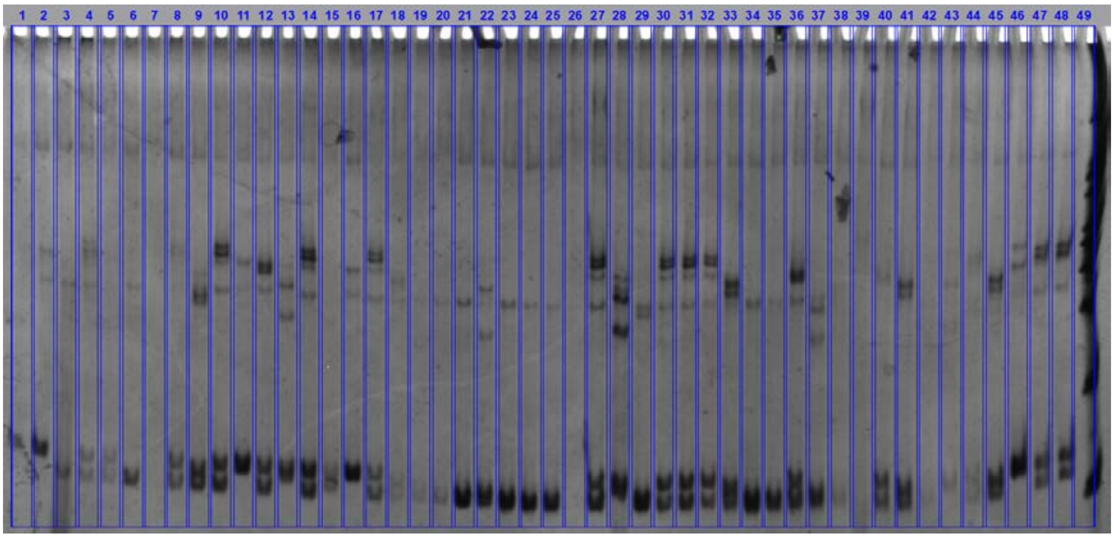
1 2 3 4 5 6 7 8 9 10 11 12 13 14 15 16 17 18 19 20 21 22 23 24 25 26 27 28 29 30 31 32 33 34 35 36 37 38 39 40 41 42 43 44 45 46 47 48 49
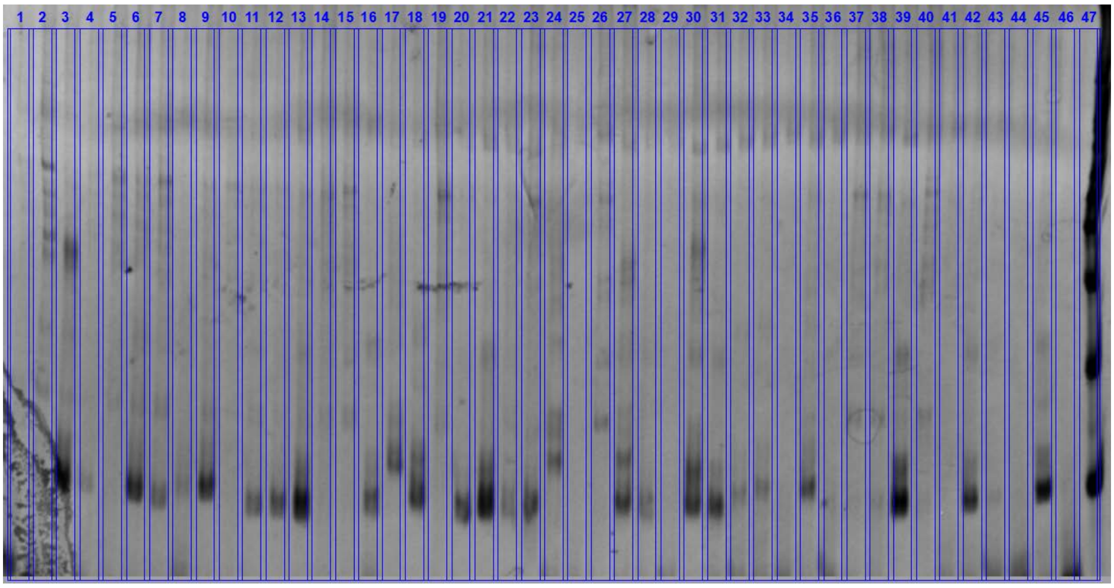
1 2 3 4 5 6 7 8 9 10 11 12 13 14 15 16 17 18 19 20 21 22 23 24 25 26 27 28 29 30 31 32 33 34 35 36 37 38 39 40 41 42 43 44 45 46 47
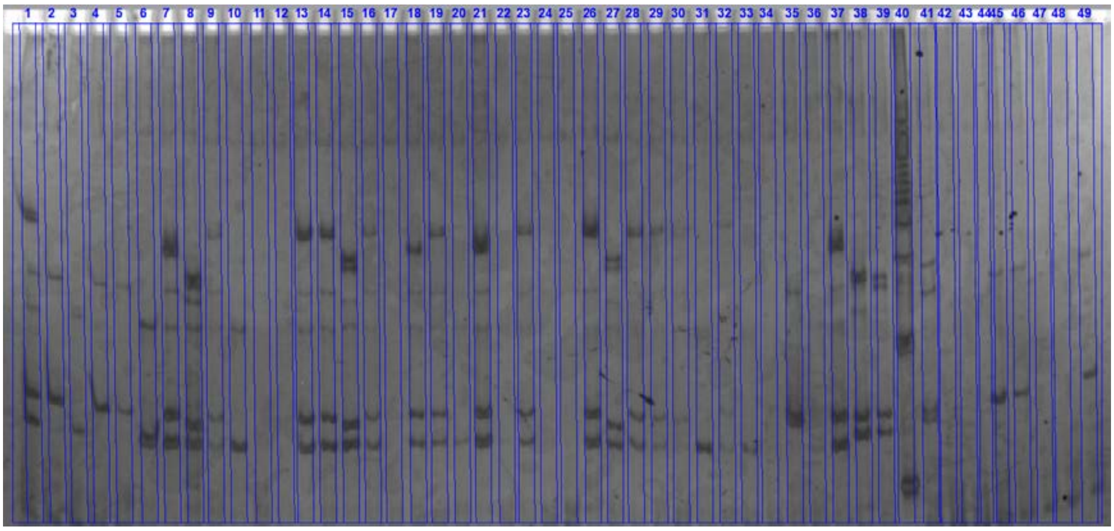
1 2 3 4 5 6 7 8 9 10 11 12 13 14 15 16 17 18 19 20 21 22 23 24 25 26 27 28 29 30 31 32 33 34 34 35 36 37 38 39 40 41 42 43 4445 46 47 48 49

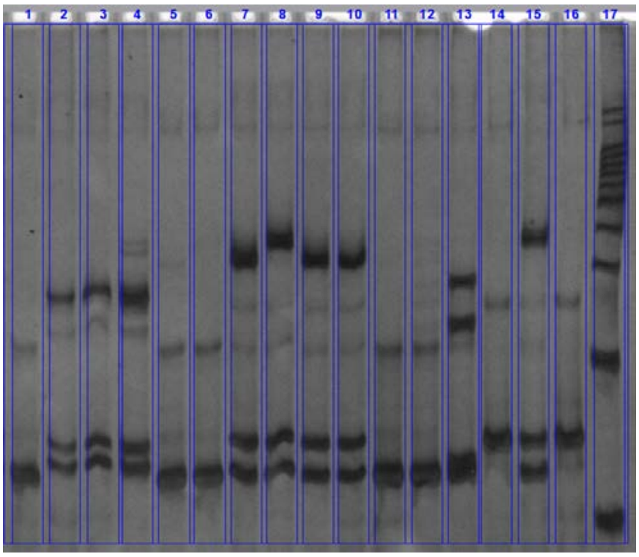

The gel images of primer L62. The accessions from left to right in each gel image were 1, 2, 3, 4, 5, 6, 7, 8, 9, 10, 11, 13, 14, 16, 18, 19, 20, 21, 22, 23, 24, 25, 26, 27, 28, 29, 30, 31, 32, 33, 34, 35, 36, 37, 38, 39, 40, 41, 42, 43, 44, 45, 46, 47, 48, 49, 53, 55, 62, 71, 72, 81, 87, 89, 94, 99, 322, 324, 328, 329, 332, 334, 339, 341, 345, 350, 354, 358, 366,367, 368, 375, 381, 401, 404, 405, 406, 407, 408, 409, 421, 442, 443, 449, 455, 457, 459, 460, 462, 463, 464, 501, 502, 503, 504, 506, 507, 508, 509, 510, 511, 513, 516, 517, 518, 544, 558, 591, 592, 593, 594, 595, 596, 621, 622, 624, 626, 628, 754, 759, 771, 1001, 1002, 1003, 1004, 1005, 1006, BF, BX, BY01, BY02, BY03, JTY, KT, HW01, HW04, HW10, HW10, HW14, HW18, HW19, HW20, HL01, HL06, HL07, HL09, HL12, HL13, HL14, HZ03, HZ07, HZ09, HZ10, HZ11, HZ13, HZ14, XB.

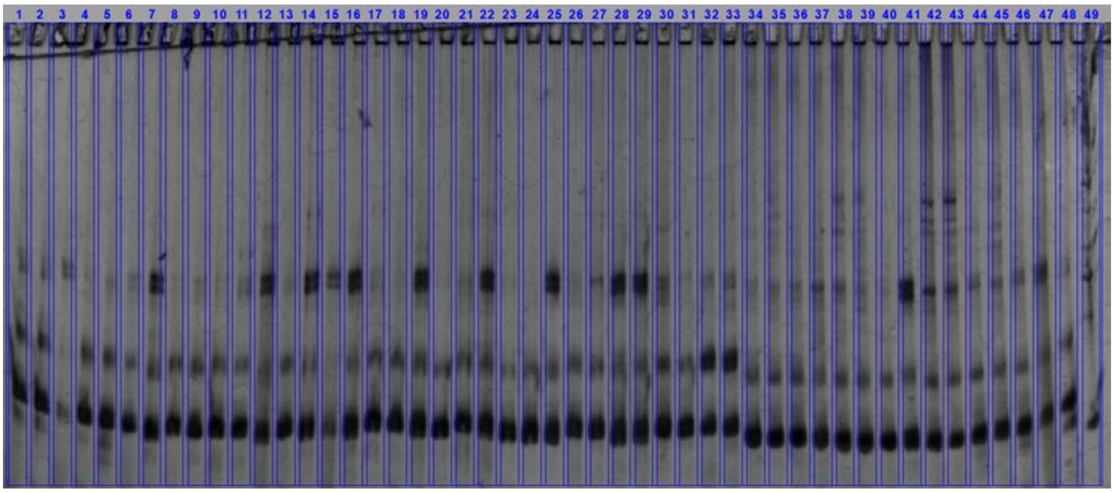
1 2 3 4 5 6 7 8 9 10 11 12 13 14 15 16 17 18 19 20 21 22 23 24 25 26 27 28 29 30 31 32 33 34 35 36 37 38 39 40 41 42 43 44 45 46 47 48 49

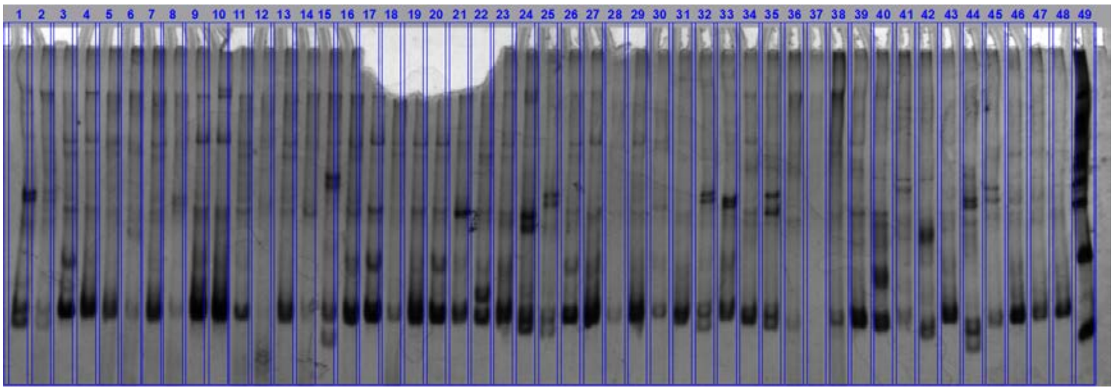
1 2 3 4 5 6 7 8 9 10 11 12 13 14 15 16 17 18 19 20 21 22 23 24 25 26 27 28 29 30 31 32 33 34 35 36 37 38 39 40 41 42 43 44 45 46 47 48 49

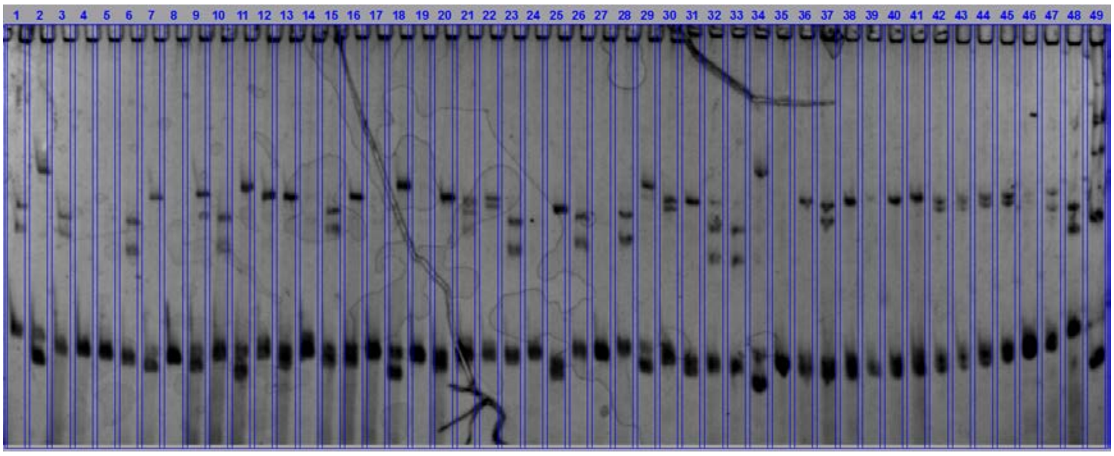
1 2 3 4 5 6 7 8 9 10 11 12 13 14 15 16 17 18 19 20 21 22 23 24 25 26 27 28 29 30 31 32 33 34 35 36 37 38 39 40 41 42 43 44 45 46 47 48 49

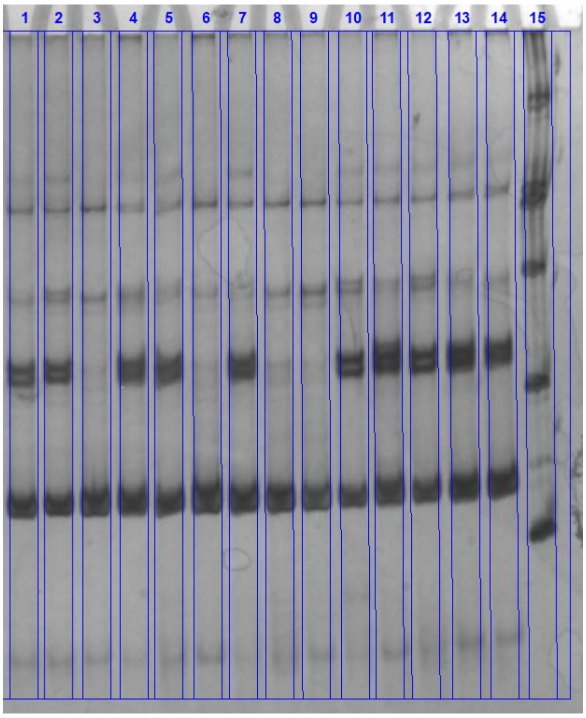

The gel images of primer L62H. The accessions from left to right in each gel image were 1, 2, 3, 4, 5, 6, 7, 8, 9, 10, 11, 13, 14, 16, 18, 19, 20, 21, 22, 23, 24, 25, 26, 27, 28, 29, 30, 31, 32, 33, 34, 35, 36, 37, 38, 39, 40, 41, 42, 43, 44, 45, 46, 47, 48, 49, 53, 55, 62, 71, 72, 81, 87, 89, 94, 99, 322, 324, 328, 329, 332, 334, 339, 341, 345, 350, 354, 358, 366,367, 368, 375, 381, 401, 404, 405, 406, 407, 408, 409, 421, 442, 443, 449, 455, 457, 459, 460, 462, 463, 464, 501, 502, 503, 504, 506, 507, 508, 509, 510, 511, 513, 516, 517, 518, 544, 558, 591, 592, 593, 594, 595, 596, 621, 622, 624, 626, 628, 754, 759, 771, 1001, 1002, 1003, 1004, 1005, 1006, BF, BX, BY01, BY02, BY03, JTY, KT, HW01, HW04, HW10, HW10, HW14, HW18, HW19, HW20, HL01, HL06, HL07, HL09, HL12, HL13, HL14, HZ03, HZ07, HZ09, HZ10, HZ11, HZ13, HZ14, XB.

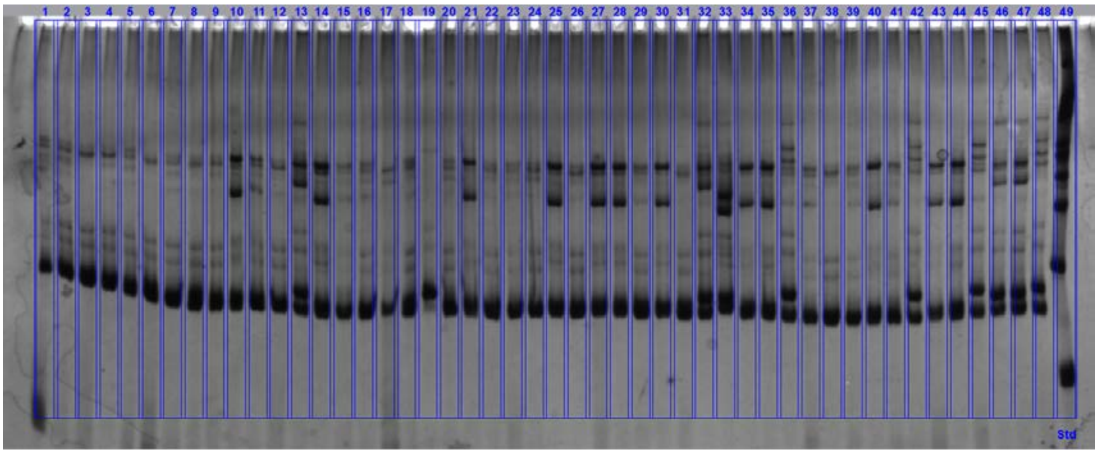
1 2 3 4 5 6 7 8 9 10 11 12 13 14 15 16 17 18 19 20 21 22 23 24 25 26 27 28 29 30 31 32 33 34 35 36 37 38 39 40 41 42 43 44 45 46 47 48 49
Std

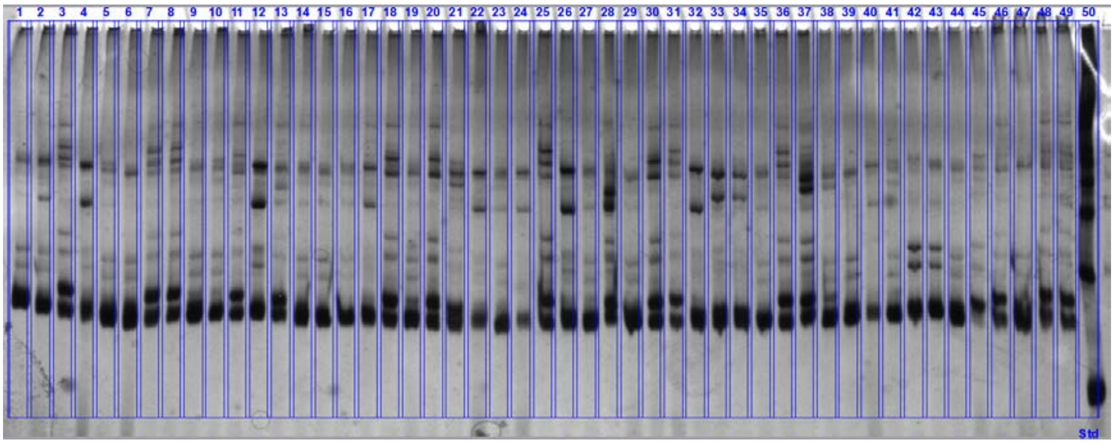
1 2 3 4 5 6 7 8 9 10 11 12 13 14 15 16 17 18 19 20 21 22 23 24 25 26 27 28 29 30 31 32 33 34 35 36 37 38 39 40 41 42 43 44 45 46 47 48 49 50
Std

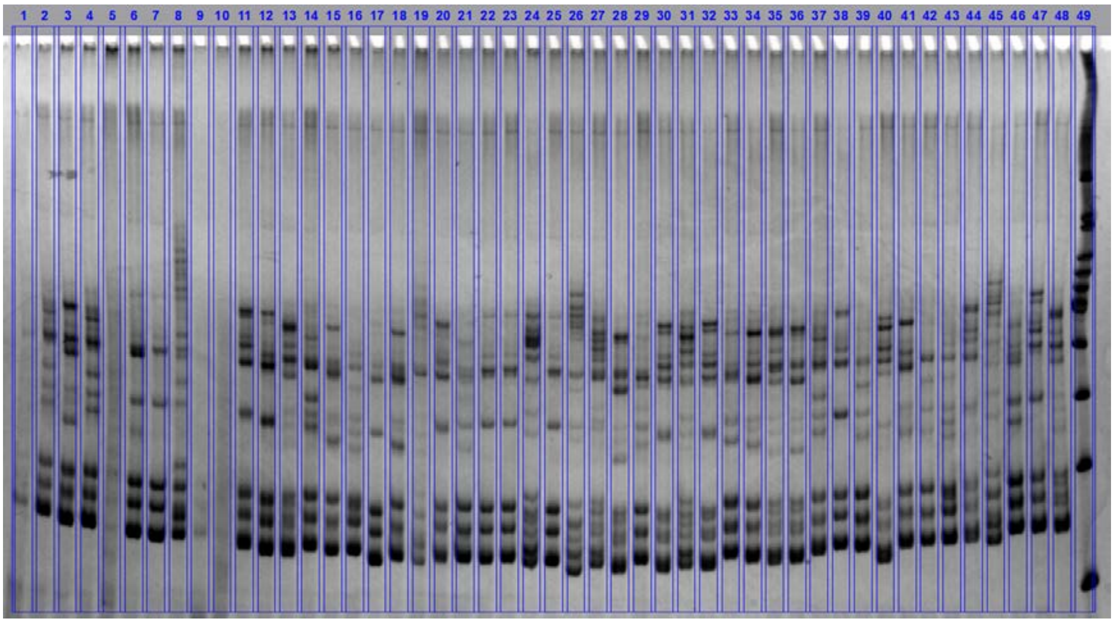
1 2 3 4 5 6 7 8 9 10 11 12 13 14 15 16 17 18 19 20 21 22 23 24 25 26 27 28 29 30 31 32 33 34 35 36 37 38 39 40 41 42 43 44 45 46 47 48 49

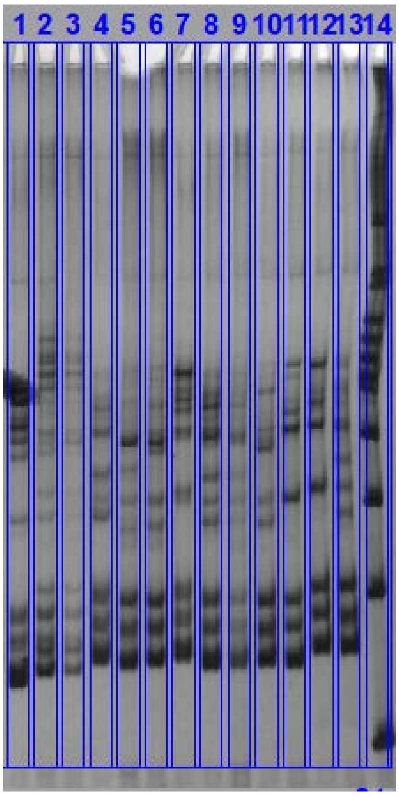

The gel images of primer L70H. The accessions from left to right in each gel image were 1, 2, 3, 4, 5, 6, 7, 8, 9, 10, 11, 13, 14, 16, 18, 19, 20, 21, 22, 23, 24, 25, 26, 27, 28, 29, 30, 31, 32, 33, 34, 35, 36, 37, 38, 39, 40, 41, 42, 43, 44, 45, 46, 47, 48, 49, 53, 55, 62, 71, 72, 81, 87, 89, 94, 99, 322, 324, 328, 329, 332, 334, 339, 341, 345, 350, 354, 358, 366,367, 368, 375, 381, 401, 404, 405, 406, 407, 408, 409, 421, 442, 443, 449, 455, 457, 459, 460, 462, 463, 464, 501, 502, 503, 504, 506, 507, 508, 509, 510, 511, 513, 516, 517, 518, 544, 558, 591, 592, 593, 594, 595, 596, 621, 622, 624, 626, 628, 754, 759, 771, 1001, 1002, 1003, 1004, 1005, 1006, BF, BX, BY01, BY02, BY03, JTY, KT, HW01, HW04, HW10, HW10, HW14, HW18, HW19, HW20, HL01, HL06, HL07, HL09, HL12, HL13, HL14, HZ03, HZ07, HZ09, HZ10, HZ11, HZ13, HZ14, XB.

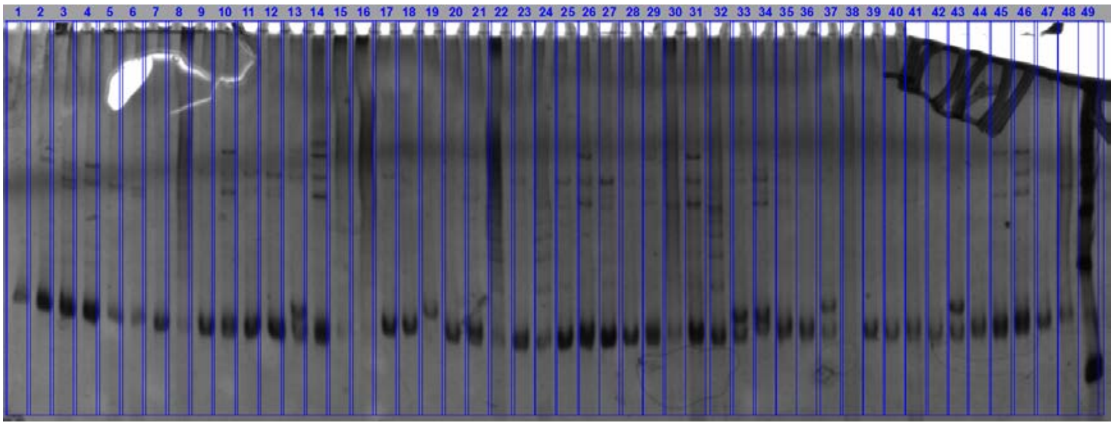
1 2 3 4 5 6 7 8 9 10 11 12 13 14 15 16 17 18 19 20 21 22 23 24 25 26 27 28 29 30 31 32 33 34 35 36 37 38 39 40 41 42 43 44 45 46 47 48 49

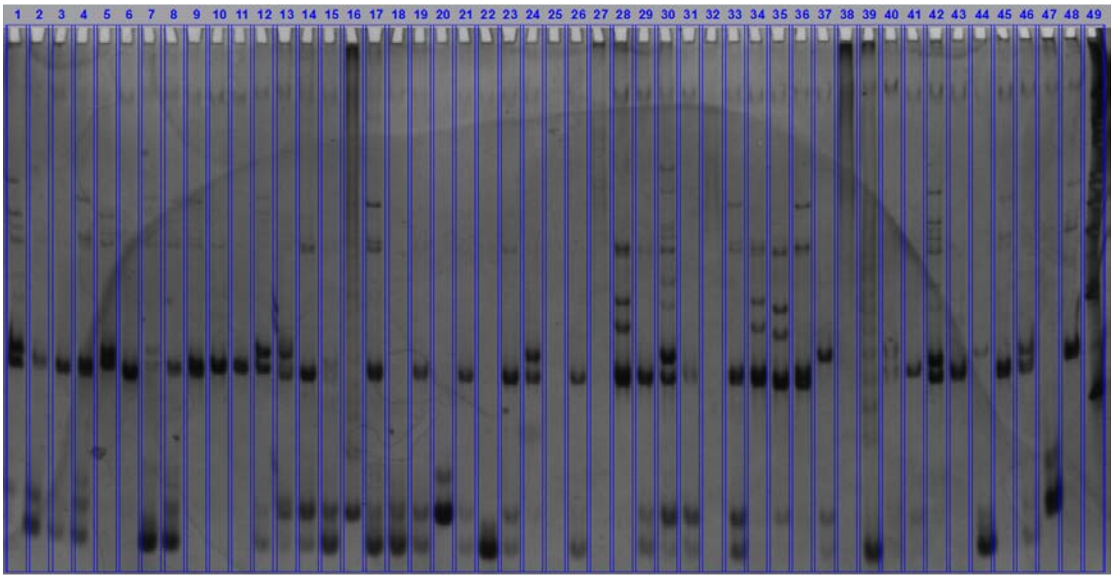
1 2 3 4 5 6 7 8 9 10 11 12 13 14 15 16 17 18 19 20 21 22 23 24 25 26 27 28 29 30 31 32 33 34 35 36 37 38 39 40 41 42 43 44 45 46 47 48 49

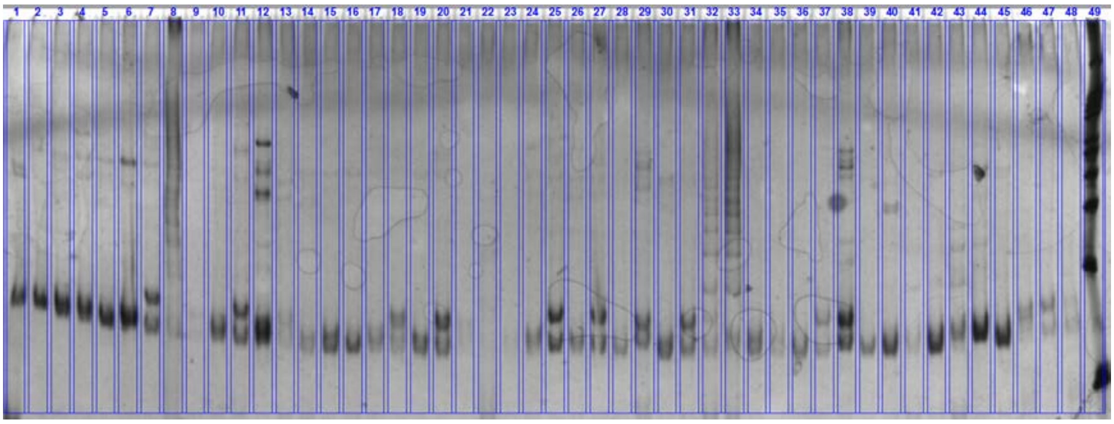
1 2 3 4 5 6 7 8 9 10 11 12 13 14 15 16 17 18 19 20 21 22 23 24 25 26 27 28 29 30 31 32 33 34 35 36 37 38 39 40 41 42 43 44 45 46 47 48 49

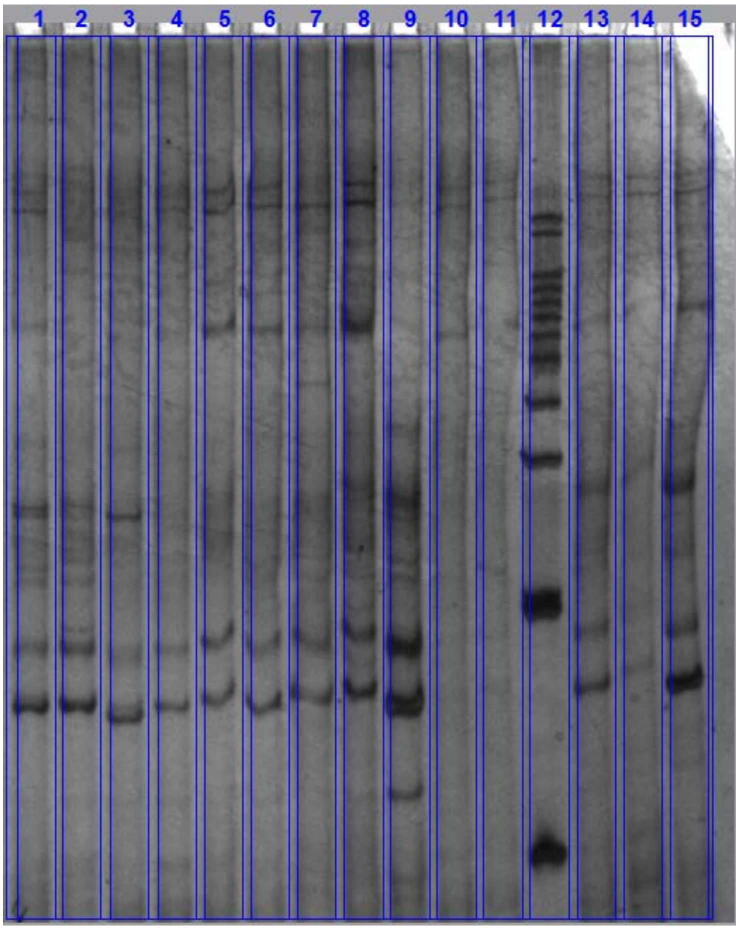

The gel images of primer L70H. The accessions from left to right in each gel image were 1, 2, 3, 4, 5, 6, 7, 8, 9, 10, 11, 13, 14, 16, 18, 19, 20, 21, 22, 23, 24, 25, 26, 27, 28, 29, 30, 31, 32, 33, 34, 35, 36, 37, 38, 39, 40, 41, 42, 43, 44, 45, 46, 47, 48, 49, 53, 55, 62, 71, 72, 81, 87, 89, 94, 99, 322, 324, 328, 329, 332, 334, 339, 341, 345, 350, 354, 358, 366,367, 368, 375, 381, 401, 404, 405, 406, 407, 408, 409, 421, 442, 443, 449, 455, 457, 459, 460, 462, 463, 464, 501, 502, 503, 504, 506, 507, 508, 509, 510, 511, 513, 516, 517, 518, 544, 558, 591, 592, 593, 594, 595, 596, 621, 622, 624, 626, 628, 754, 759, 771, 1001, 1002, 1003, 1004, 1005, 1006, BF, BX, BY01, BY02, BY03, JTY, KT, HW01, HW04, HW10, HW10, HW14, HW18, HW19, HW20, HL01, HL06, HL07, HL09, HL12, HL13, HL14, HZ03, HZ07, HZ09, HZ10, HZ11, HZ13, HZ14, XB.

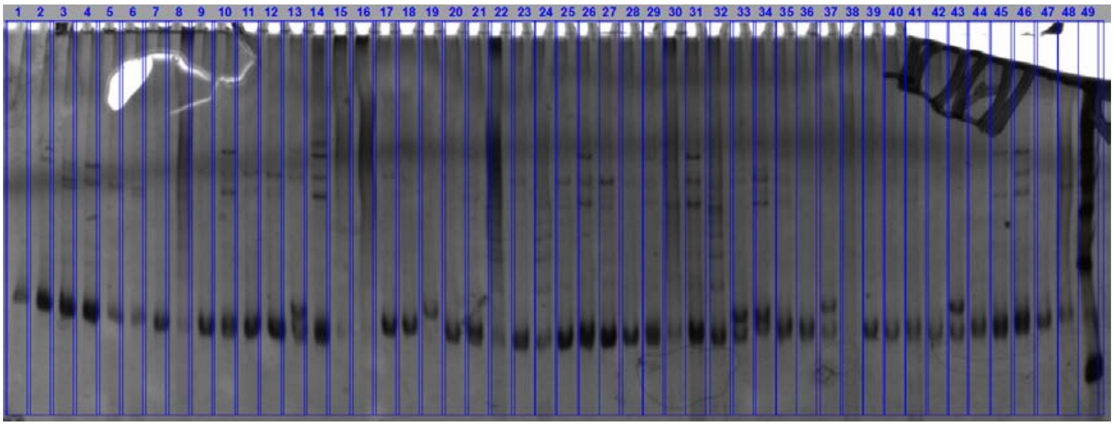
1 2 3 4 5 6 7 8 9 10 11 12 13 14 15 16 17 18 19 20 21 22 23 24 25 26 27 28 29 30 31 32 33 34 35 36 37 38 39 40 41 42 43 44 45 46 47 48 49

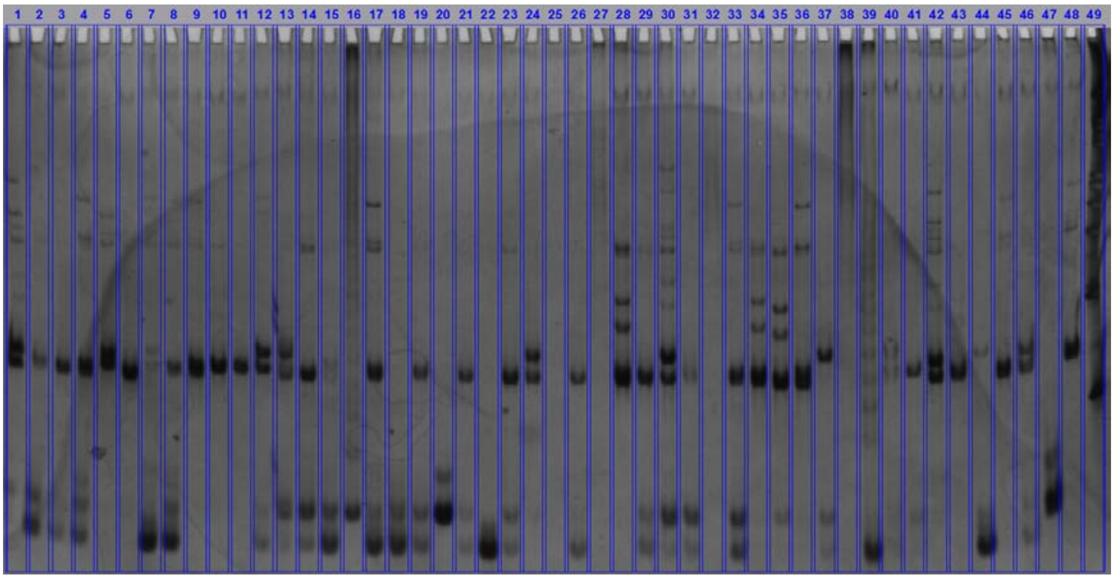
1 2 3 4 5 6 7 8 9 10 11 12 13 14 15 16 17 18 19 20 21 22 23 24 25 26 27 28 29 30 31 32 33 34 35 36 37 38 39 40 41 42 43 44 45 46 47 48 49

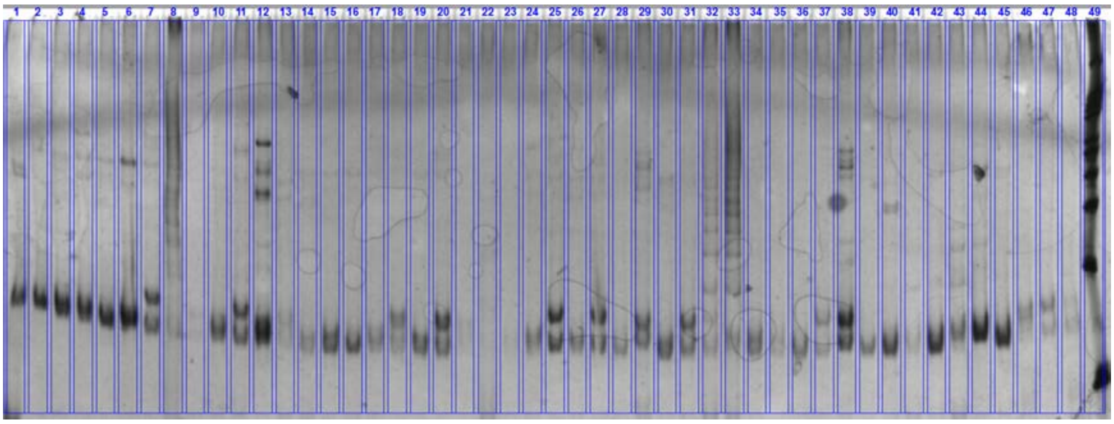
1 2 3 4 5 6 7 8 9 10 11 12 13 14 15 16 17 18 19 20 21 22 23 24 25 26 27 28 29 30 31 32 33 34 35 36 37 38 39 40 41 42 43 44 45 46 47 48 49

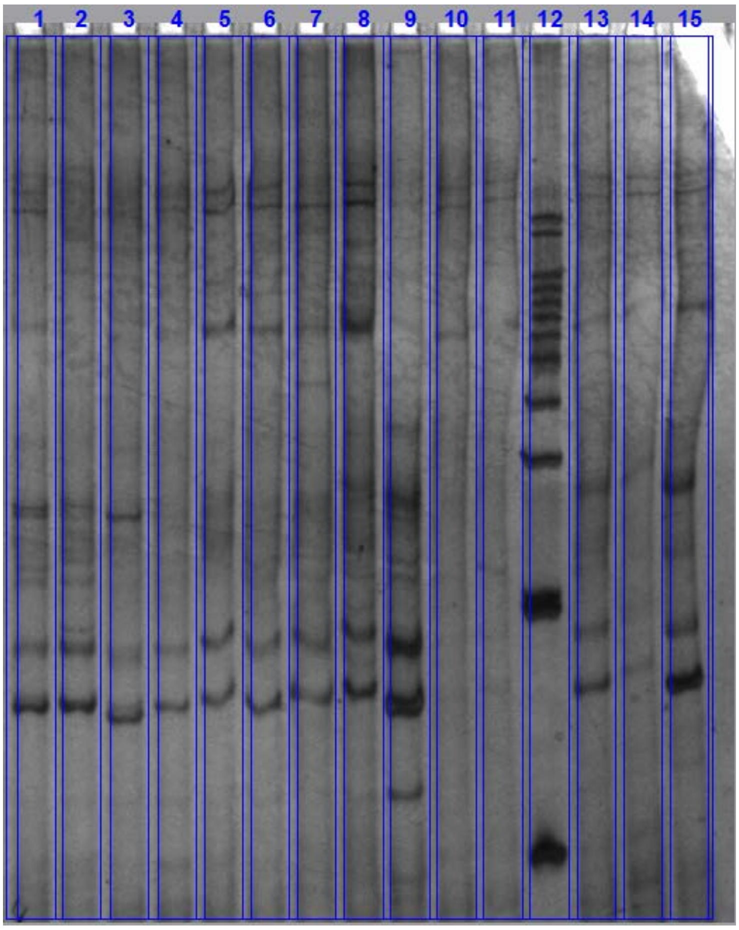

The gel images of primer L75. The accessions from left to right in each gel image were 1, 2, 3, 4, 5, 6, 7, 8, 9, 10, 11, 13, 14, 16, 18, 19, 20, 21, 22, 23, 24, 25, 26, 27, 28, 29, 30, 31, 32, 33, 34, 35, 36, 37, 38, 39, 40, 41, 42, 43, 44, 45, 46, 47, 48, 49, 53, 55, 62, 71, 72, 81, 87, 89, 94, 99, 322, 324, 328, 329, 332, 334, 339, 341, 345, 350, 354, 358, 366,367, 368, 375, 381, 401, 404, 405, 406, 407, 408, 409, 421, 442, 443, 449, 455, 457, 459, 460, 462, 463, 464, 501, 502, 503, 504, 506, 507, 508, 509, 510, 511, 513, 516, 517, 518, 544, 558, 591, 592, 593, 594, 595, 596, 621, 622, 624, 626, 628, 754, 759, 771, 1001, 1002, 1003, 1004, 1005, 1006, BF, BX, BY01, BY02, BY03, JTY, KT, HW01, HW04, HW10, HW10, HW14, HW18, HW19, HW20, HL01, HL06, HL07, HL09, HL12, HL13, HL14, HZ03, HZ07, HZ09, HZ10, HZ11, HZ13, HZ14, XB.

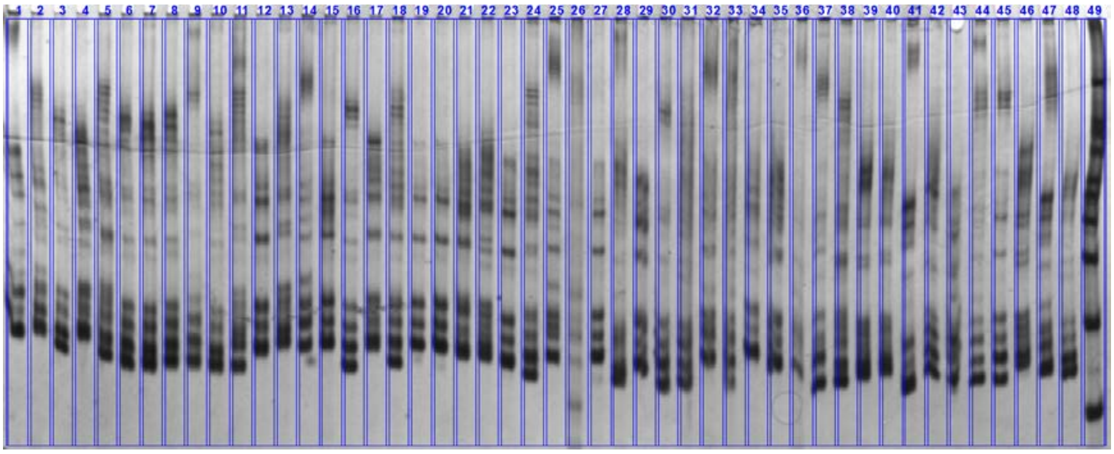
1 2 3 4 5 6 7 8 9 10 11 12 13 14 15 16 17 18 19 20 21 22 23 24 25 26 27 28 29 30 31 32 33 34 35 36 37 38 39 40 41 42 43 44 45 46 47 48 49

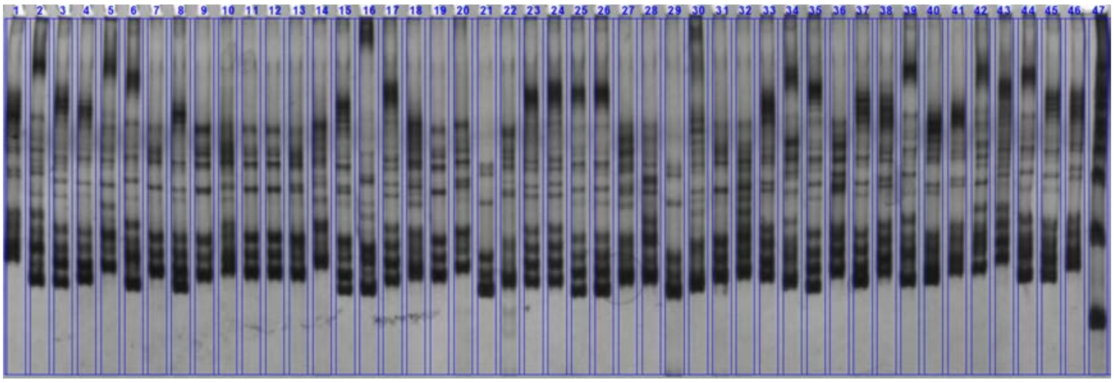
1 2 3 4 5 6 7 8 9 10 11 12 13 14 15 16 17 18 19 20 21 22 23 24 25 26 27 28 29 30 31 32 33 34 35 36 37 38 39 40 41 42 43 44 45 46 47

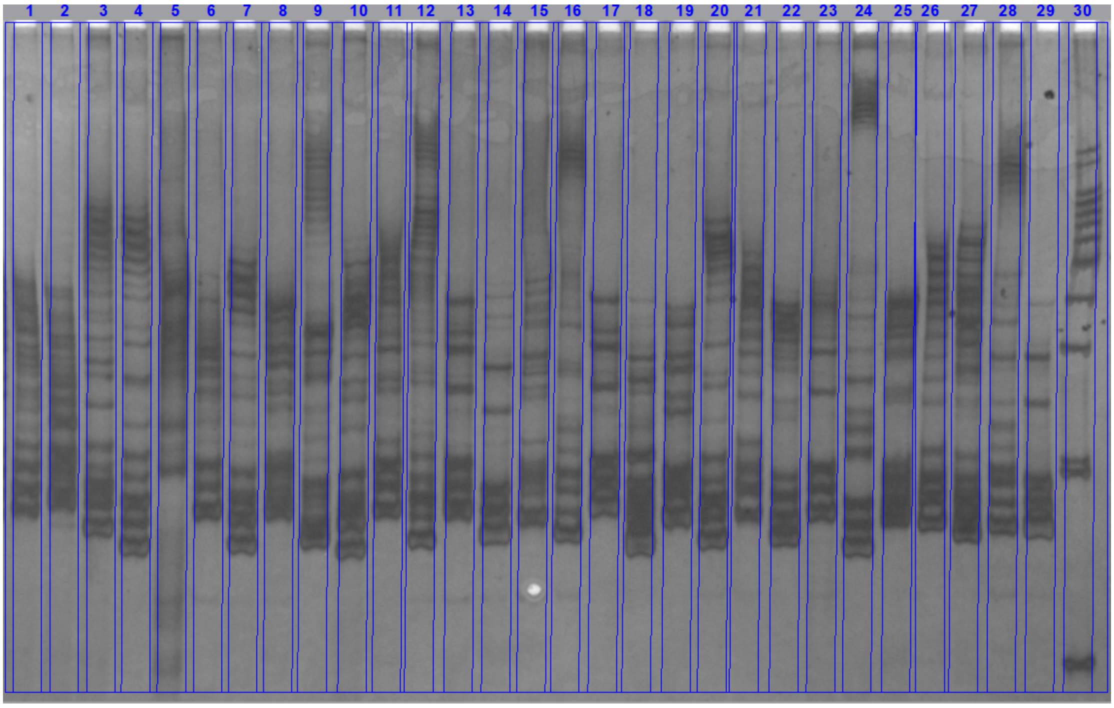
1 2 3 4 5 6 7 8 9 10 11 12 13 14 15 16 17 18 19 20 21 22 23 24 25 26 27 28 29 30

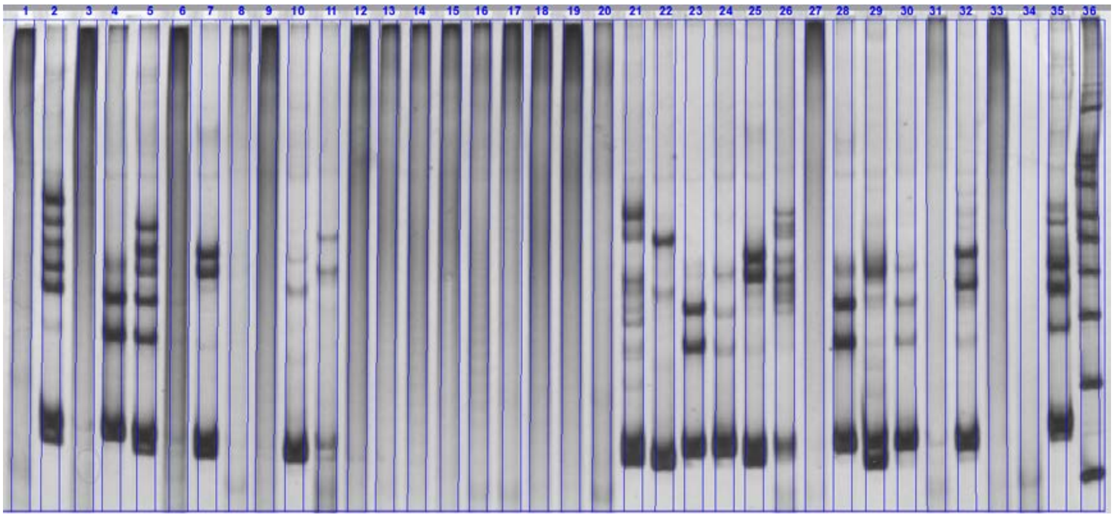

The gel images of primer L79H. The accessions from left to right in each gel image were 1, 2, 3, 4, 5, 6, 7, 8, 9, 10, 11, 13, 14, 16, 18, 19, 20, 21, 22, 23, 24, 25, 26, 27, 28, 29, 30, 31, 32, 33, 34, 35, 36, 37, 38, 39, 40, 41, 42, 43, 44, 45, 46, 47, 48, 49, 53, 55, 62, 71, 72, 81, 87, 89, 94, 99, 322, 324, 328, 329, 332, 334, 339, 341, 345, 350, 354, 358, 366,367, 368, 375, 381, 401, 404, 405, 406, 407, 408, 409, 421, 442, 443, 449, 455, 457, 459, 460, 462, 463, 464, 501, 502, 503, 504, 506, 507, 508, 509, 510, 511, 513, 516, 517, 518, 544, 558, 591, 592, 593, 594, 595, 596, 621, 622, 624, 626, 628, 754, 759, 771, 1001, 1002, 1003, 1004, 1005, 1006, BF, BX, BY01, BY02, BY03, JTY, KT, HW01, HW04, HW10, HW10, HW14, HW18, HW19, HW20, HL01, HL06, HL07, HL09, HL12, HL13, HL14, HZ03, HZ07, HZ09, HZ10, HZ11, HZ13, HZ14, XB.

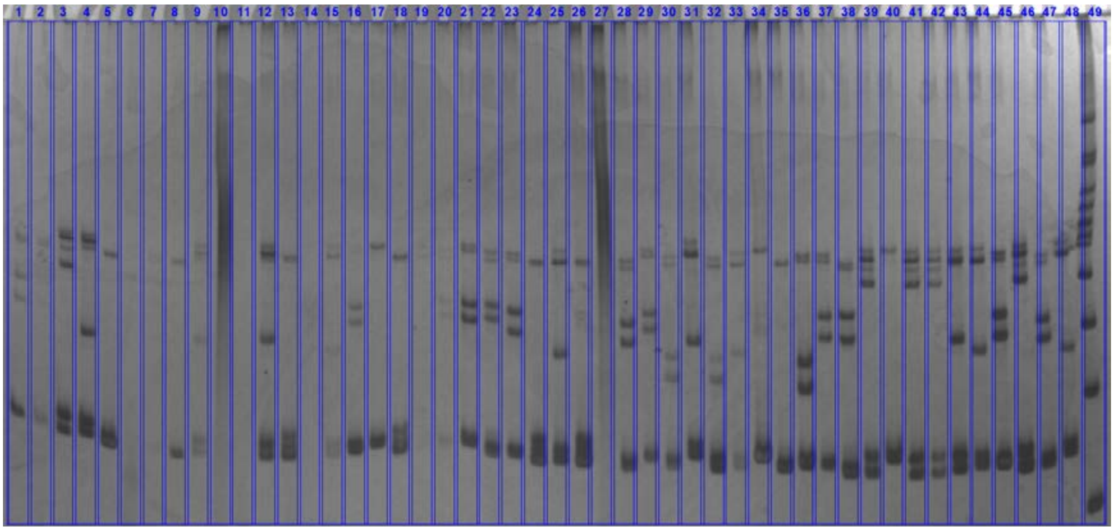
1 2 3 4 5 6 7 8 9 10 11 12 13 14 15 16 17 18 19 20 21 22 23 24 25 26 27 28 29 30 31 32 33 34 35 36 37 38 39 40 41 42 43 44 45 46 47 48 49

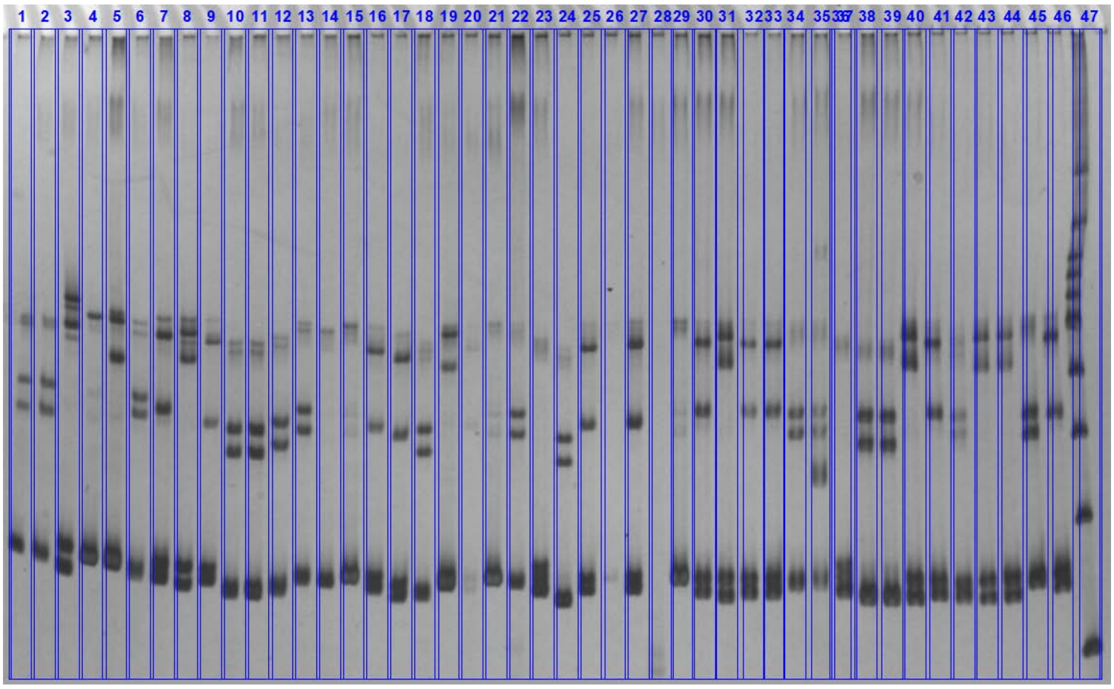
1 2 3 4 5 6 7 8 9 10 11 12 13 14 15 16 17 18 19 20 21 22 23 24 25 26 27 28 29 30 31 32 33 34 35 36 37 38 39 40 41 42 43 44 45 46 47

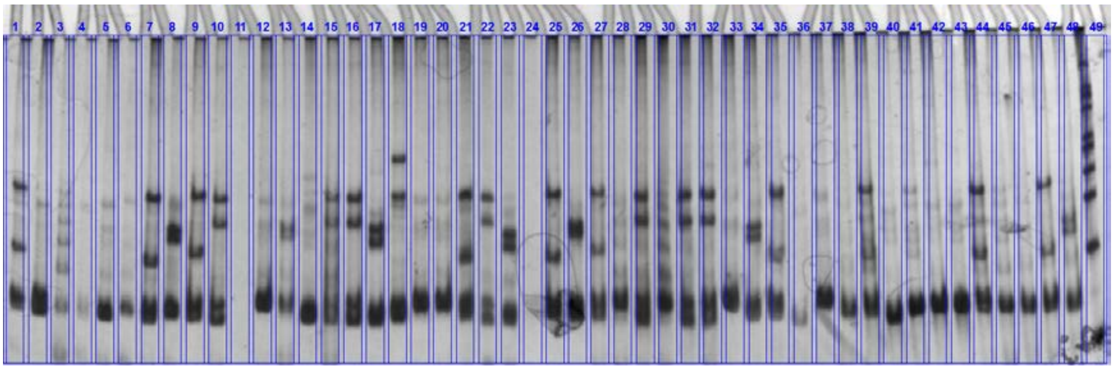
1 2 3 4 5 6 7 8 9 10 11 12 13 14 15 16 17 18 19 20 21 22 23 24 25 26 27 28 29 30 31 32 33 34 35 36 37 38 39 40 41 42 43 44 45 46 47 48 49

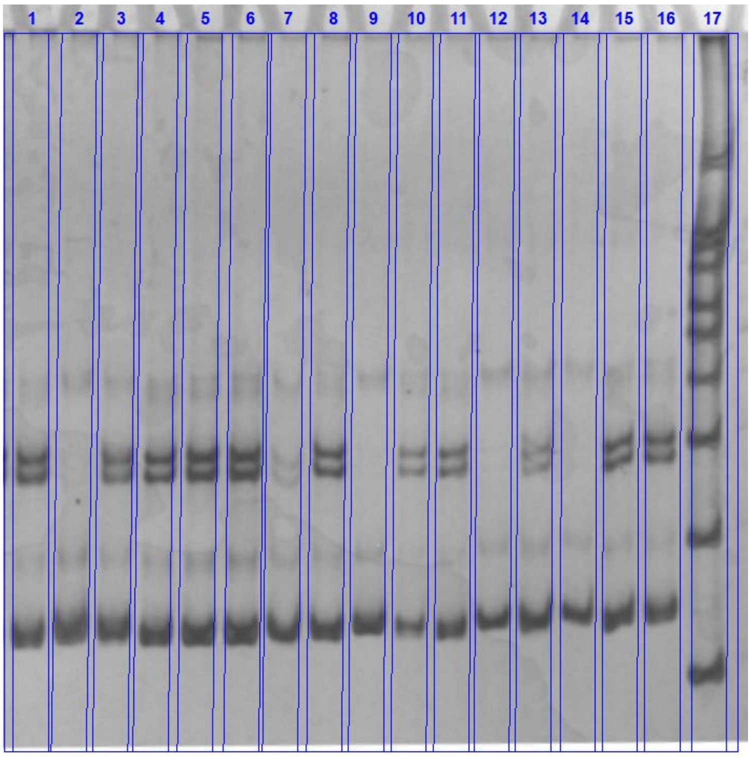

The gel images of primer P3. The accessions from left to right in each gel image were 1, 2, 3, 4, 5, 6, 7, 8, 9, 10, 11, 13, 14, 16, 18, 19, 20, 21, 22, 23, 24, 25, 26, 27, 28, 29, 30, 31, 32, 33, 34, 35, 36, 37, 38, 39, 40, 41, 42, 43, 44, 45, 46, 47, 48, 49, 53, 55, 62, 71, 72, 81, 87, 89, 94, 99, 322, 324, 328, 329, 332, 334, 339, 341, 345, 350, 354, 358, 366,367, 368, 375, 381, 401, 404, 405, 406, 407, 408, 409, 421, 442, 443, 449, 455, 457, 459, 460, 462, 463, 464, 501, 502, 503, 504, 506, 507, 508, 509, 510, 511, 513, 516, 517, 518, 544, 558, 591, 592, 593, 594, 595, 596, 621, 622, 624, 626, 628, 754, 759, 771, 1001, 1002, 1003, 1004, 1005, 1006, BF, BX, BY01, BY02, BY03, JTY, KT, HW01, HW04, HW10, HW10, HW14, HW18, HW19, HW20, HL01, HL06, HL07, HL09, HL12, HL13, HL14, HZ03, HZ07, HZ09, HZ10, HZ11, HZ13, HZ14, XB.

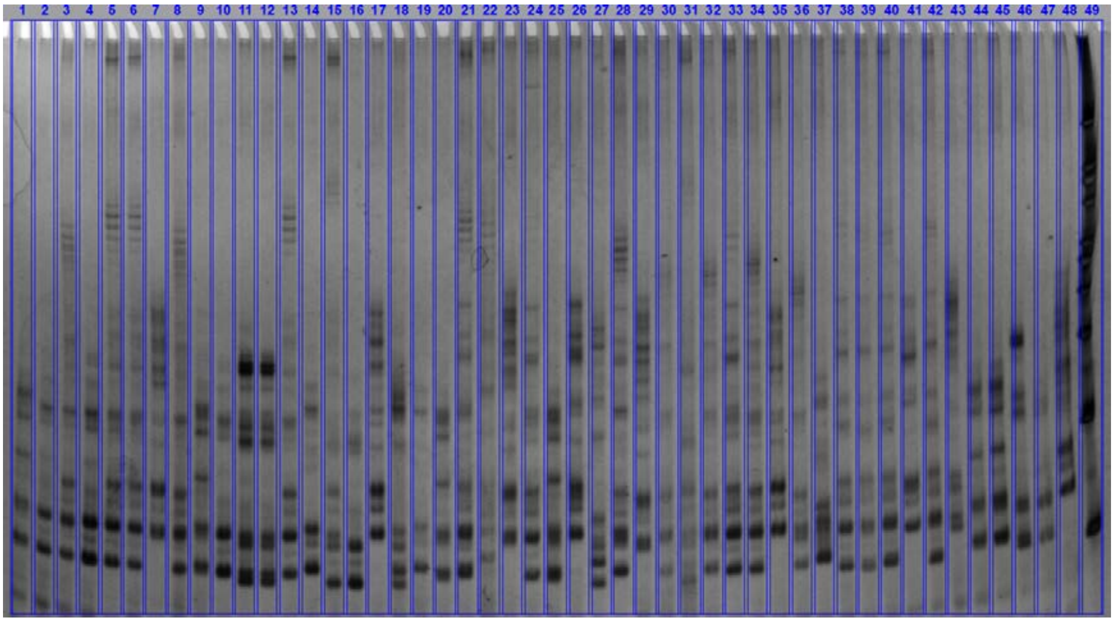
1 2 3 4 5 6 7 8 9 10 11 12 13 14 15 16 17 18 19 20 21 22 23 24 25 26 27 28 29 30 31 32 33 34 35 36 37 38 39 40 41 42 43 44 45 46 47 48 49

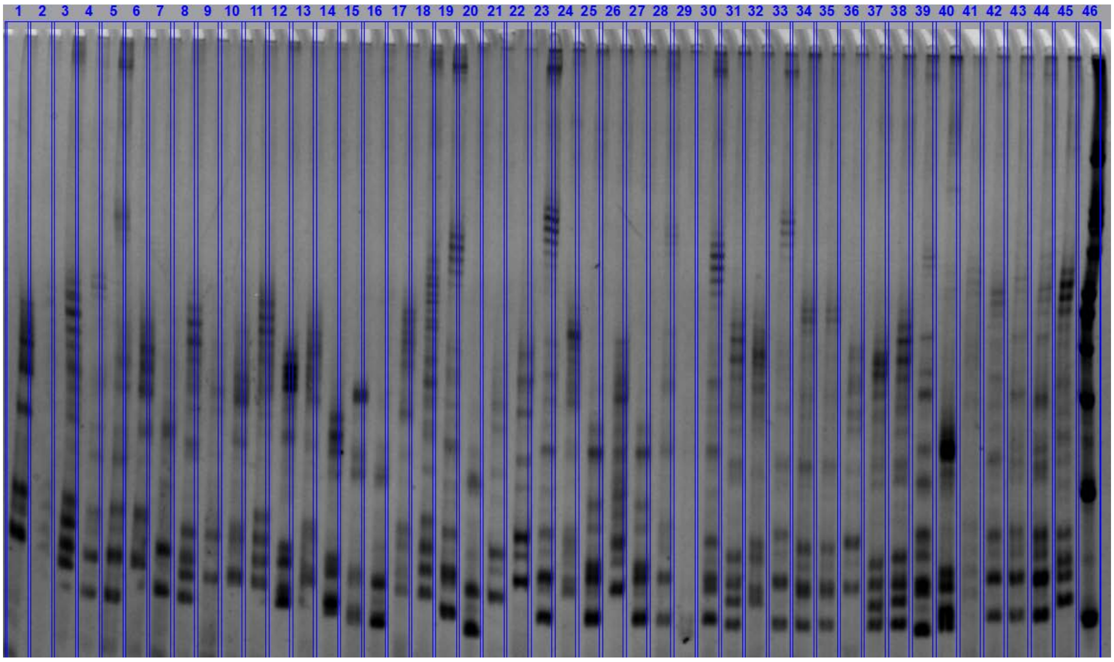
1 2 3 4 5 6 7 8 9 10 11 12 13 14 15 16 17 18 19 20 21 22 23 24 25 26 27 28 29 30 31 32 33 34 35 36 37 38 39 40 41 42 43 44 45 46

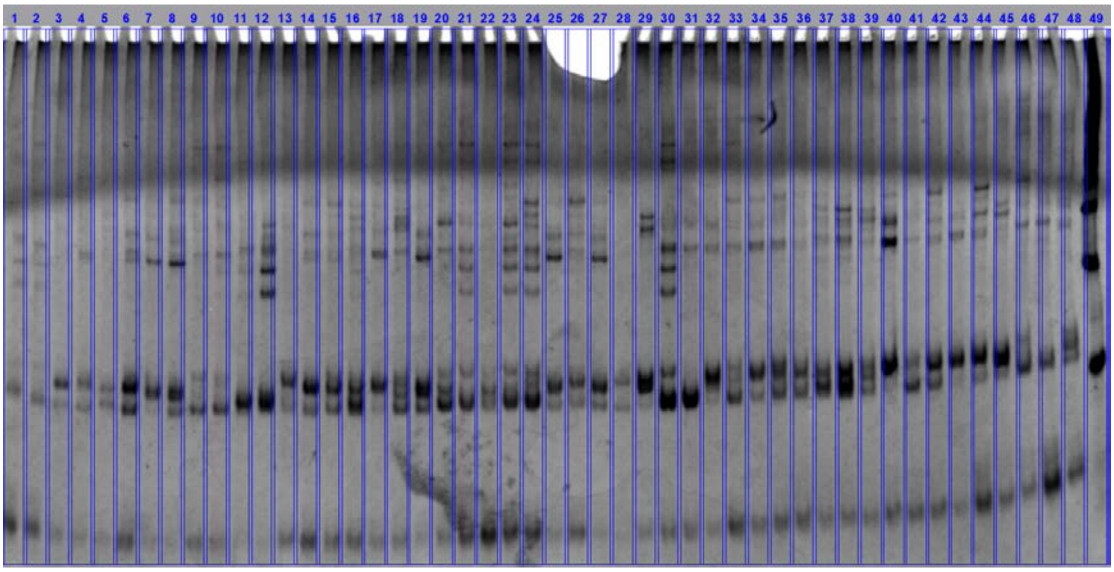

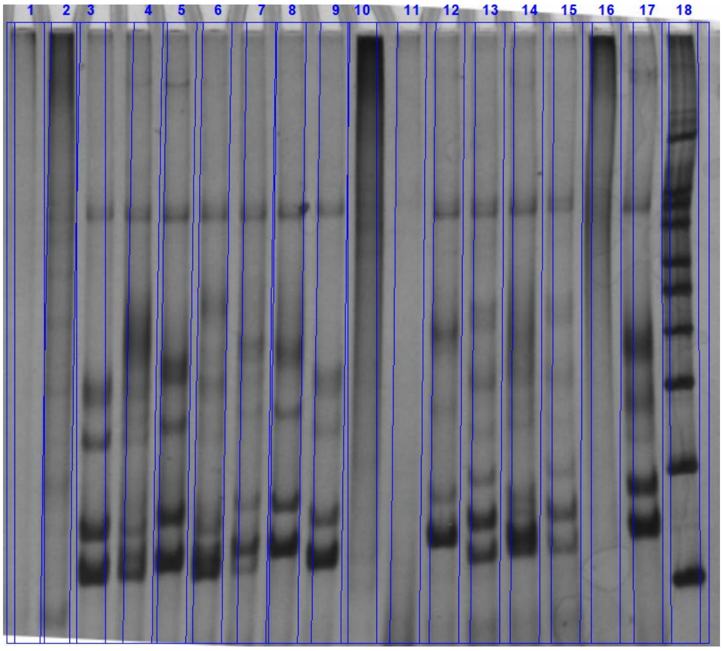

The gel images of primer P21. The accessions from left to right in each gel image were 1, 2, 3, 4, 5, 6, 7, 8, 9, 10, 11, 13, 14, 16, 18, 19, 20, 21, 22, 23, 24, 25, 26, 27, 28, 29, 30, 31, 32, 33, 34, 35, 36, 37, 38, 39, 40, 41, 42, 43, 44, 45, 46, 47, 48, 49, 53, 55, 62, 71, 72, 81, 87, 89, 94, 99, 322, 324, 328, 329, 332, 334, 339, 341, 345, 350, 354, 358, 366,367, 368, 375, 381, 401, 404, 405, 406, 407, 408, 409, 421, 442, 443, 449, 455, 457, 459, 460, 462, 463, 464, 501, 502, 503, 504, 506, 507, 508, 509, 510, 511, 513, 516, 517, 518, 544, 558, 591, 592, 593, 594, 595, 596, 621, 622, 624, 626, 628, 754, 759, 771, 1001, 1002, 1003, 1004, 1005, 1006, BF, BX, BY01, BY02, BY03, JTY, KT, HW01, HW04, HW10, HW10, HW14, HW18, HW19, HW20, HL01, HL06, HL07, HL09, HL12, HL13, HL14, HZ03, HZ07, HZ09, HZ10, HZ11, HZ13, HZ14, XB.

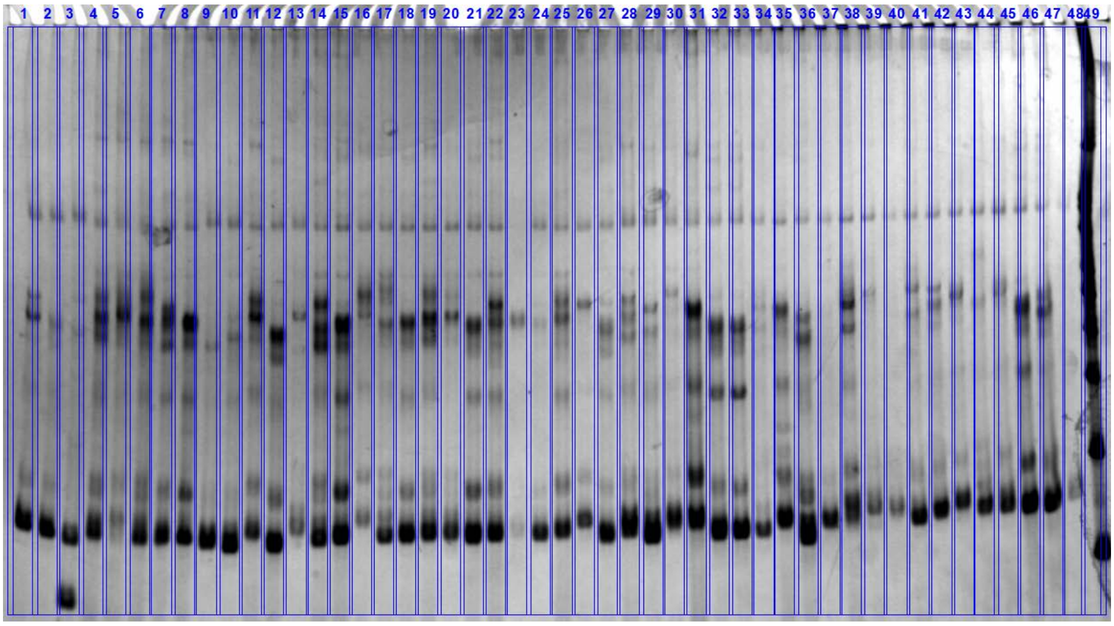
1 2 3 4 5 6 7 8 9 10 11 12 13 14 15 16 17 18 19 20 21 22 23 24 25 26 27 28 29 30 31 32 33 34 35 36 37 38 39 40 41 42 43 44 45 46 47 48 49

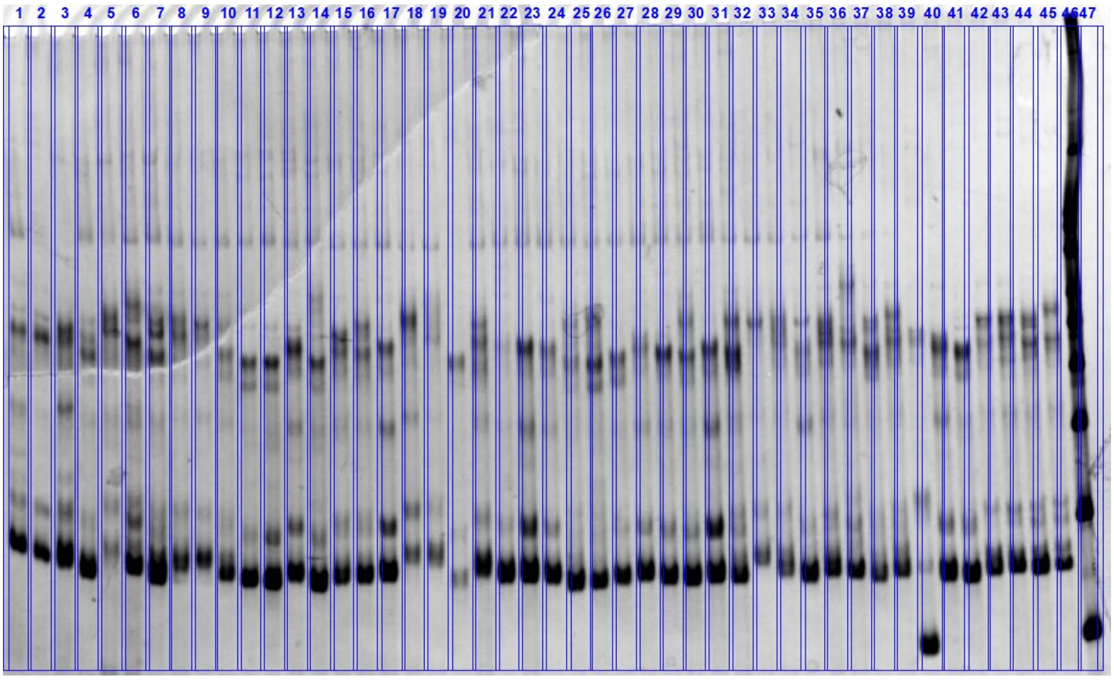
1 2 3 4 5 6 7 8 9 10 11 12 13 14 15 16 17 18 19 20 21 22 23 24 25 26 27 28 29 30 31 32 33 34 35 36 37 38 39 40 41 42 43 44 45 46 47

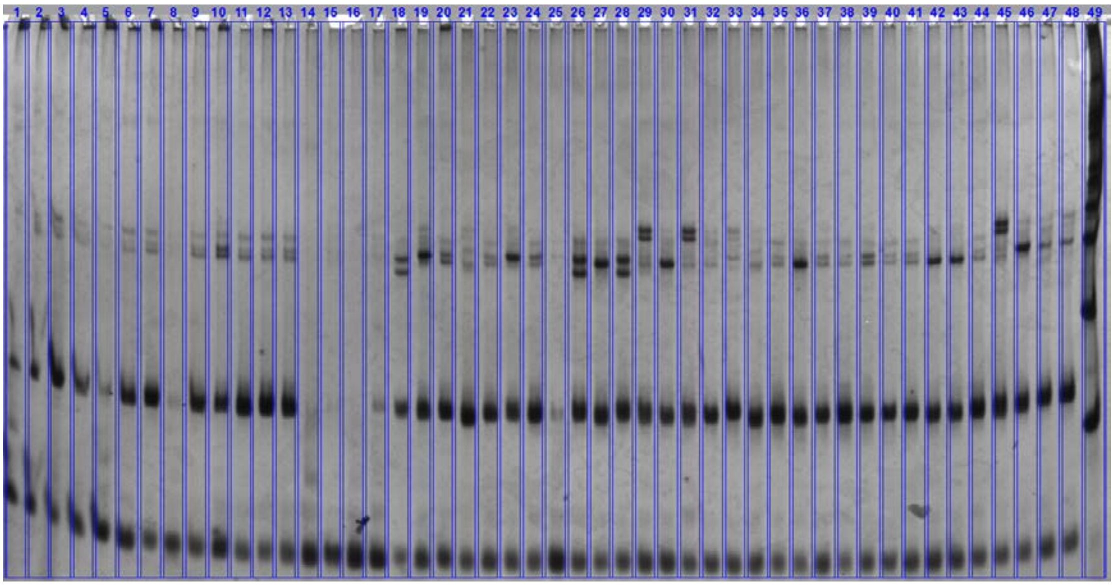

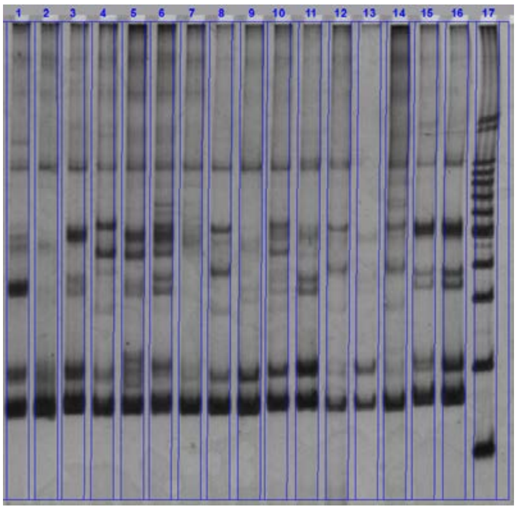

The gel images of primer P40H. The accessions from left to right in each gel image were 1, 2, 3, 4, 5, 6, 7, 8, 9, 10, 11, 13, 14, 16, 18, 19, 20, 21, 22, 23, 24, 25, 26, 27, 28, 29, 30, 31, 32, 33, 34, 35, 36, 37, 38, 39, 40, 41, 42, 43, 44, 45, 46, 47, 48, 49, 53, 55, 62, 71, 72, 81, 87, 89, 94, 99, 322, 324, 328, 329, 332, 334, 339, 341, 345, 350, 354, 358, 366,367, 368, 375, 381, 401, 404, 405, 406, 407, 408, 409, 421, 442, 443, 449, 455, 457, 459, 460, 462, 463, 464, 501, 502, 503, 504, 506, 507, 508, 509, 510, 511, 513, 516, 517, 518, 544, 558, 591, 592, 593, 594, 595, 596, 621, 622, 624, 626, 628, 754, 759, 771, 1001, 1002, 1003, 1004, 1005, 1006, BF, BX, BY01, BY02, BY03, JTY, KT, HW01, HW04, HW10, HW10, HW14, HW18, HW19, HW20, HL01, HL06, HL07, HL09, HL12, HL13, HL14, HZ03, HZ07, HZ09, HZ10, HZ11, HZ13, HZ14, XB.

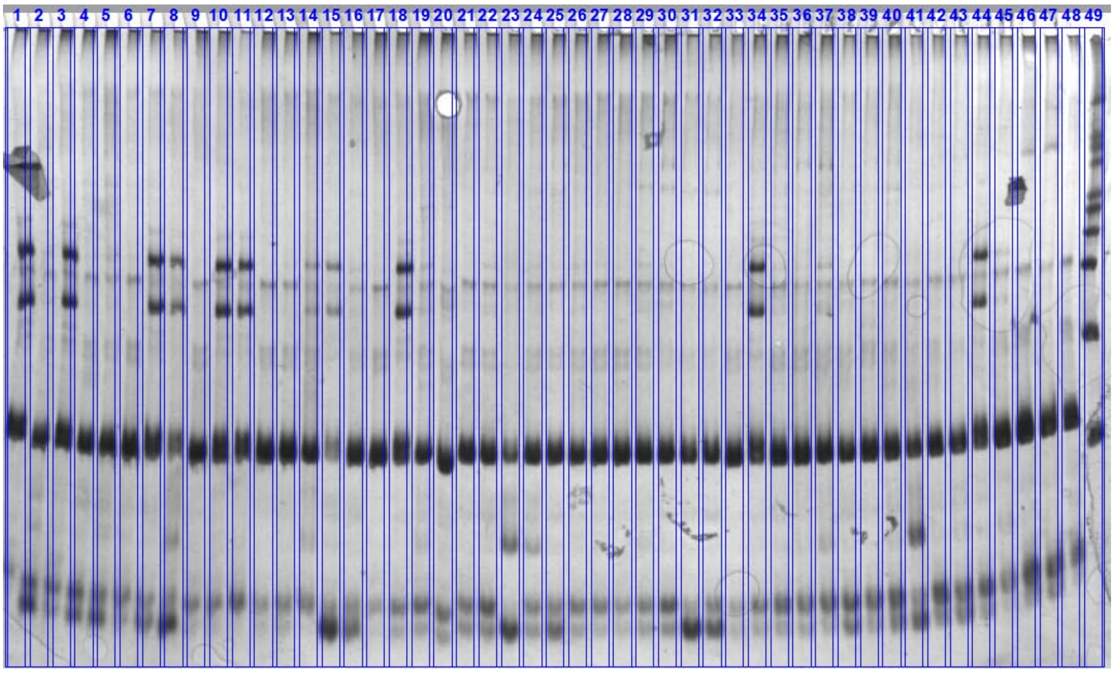
1 2 3 4 5 6 7 8 9 10 11 12 13 14 15 16 17 18 19 20 21 22 23 24 25 26 27 28 29 30 31 32 33 34 35 36 37 38 39 40 41 42 43 44 45 46 47 48 49

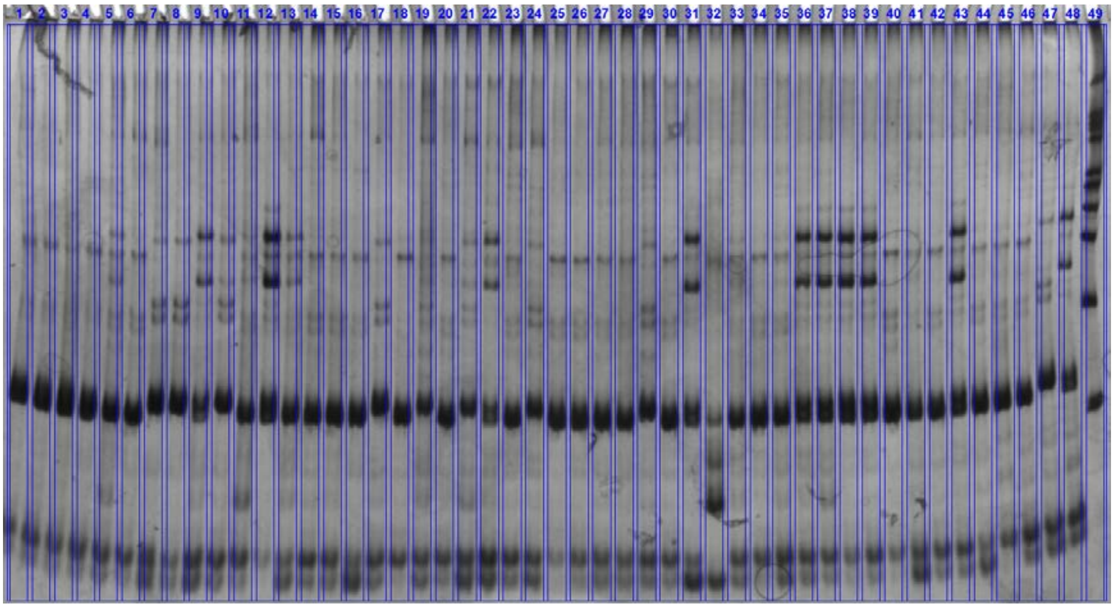
1 2 3 4 5 6 7 8 9 10 11 12 13 14 15 16 17 18 19 20 21 22 23 24 25 26 27 28 29 30 31 32 33 34 35 36 37 38 39 40 41 42 43 44 45 46 47 48 49

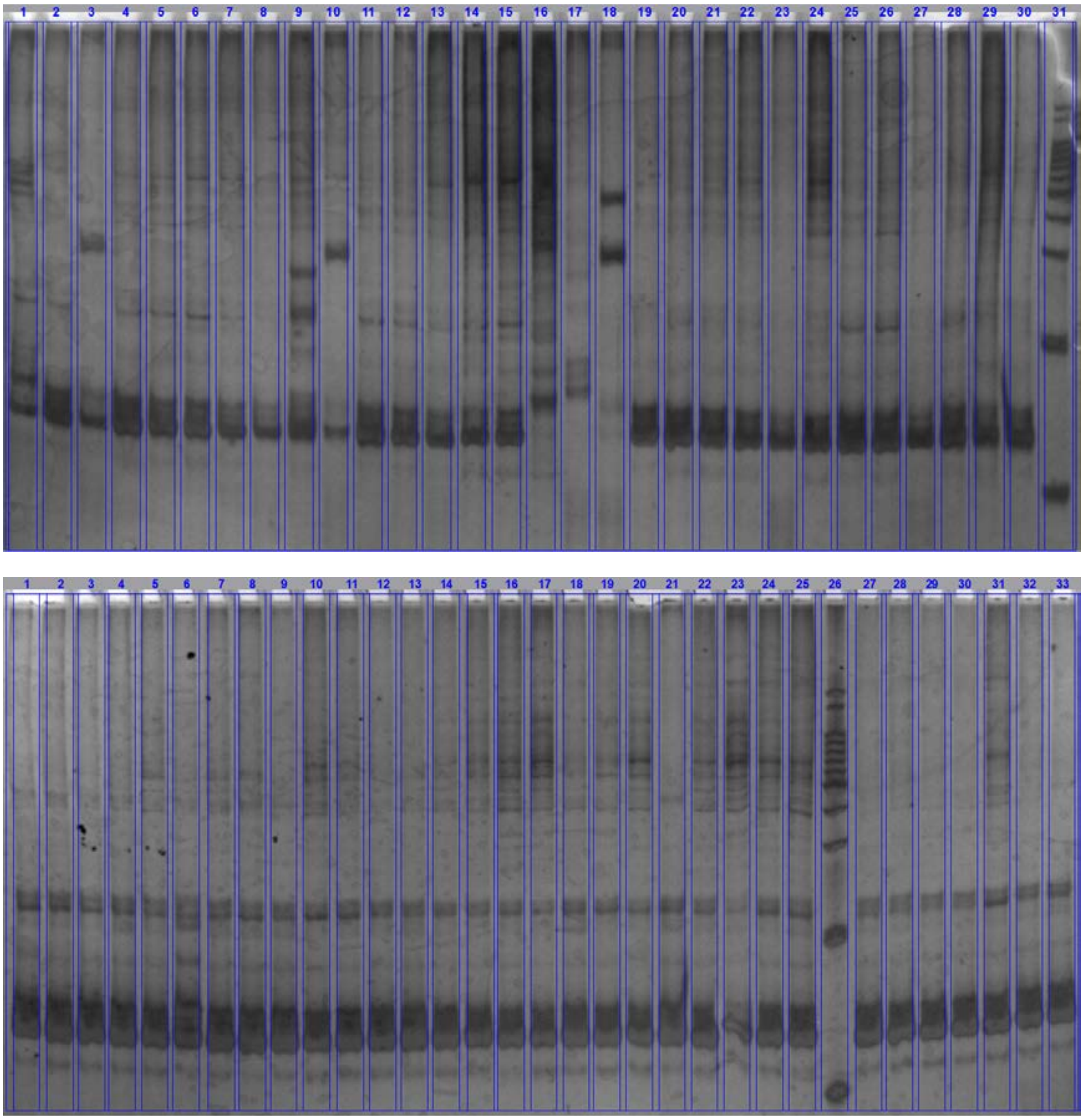

The gel images of primer P57H. The accessions from left to right in each gel image were 1, 2, 3, 4, 5, 6, 7, 8, 9, 10, 11, 13, 14, 16, 18, 19, 20, 21, 22, 23, 24, 25, 26, 27, 28, 29, 30, 31, 32, 33, 34, 35, 36, 37, 38, 39, 40, 41, 42, 43, 44, 45, 46, 47, 48, 49, 53, 55, 62, 71, 72, 81, 87, 89, 94, 99, 322, 324, 328, 329, 332, 334, 339, 341, 345, 350, 354, 358, 366,367, 368, 375, 381, 401, 404, 405, 406, 407, 408, 409, 421, 442, 443, 449, 455, 457, 459, 460, 462, 463, 464, 501, 502, 503, 504, 506, 507, 508, 509, 510, 511, 513, 516, 517, 518, 544, 558, 591, 592, 593, 594, 595, 596, 621, 622, 624, 626, 628, 754, 759, 771, 1001, 1002, 1003, 1004, 1005, 1006, BF, BX, BY01, BY02, BY03, JTY, KT, HW01, HW04, HW10, HW10, HW14, HW18, HW19, HW20, HL01, HL06, HL07, HL09, HL12, HL13, HL14, HZ03, HZ07, HZ09, HZ10, HZ11, HZ13, HZ14, XB.

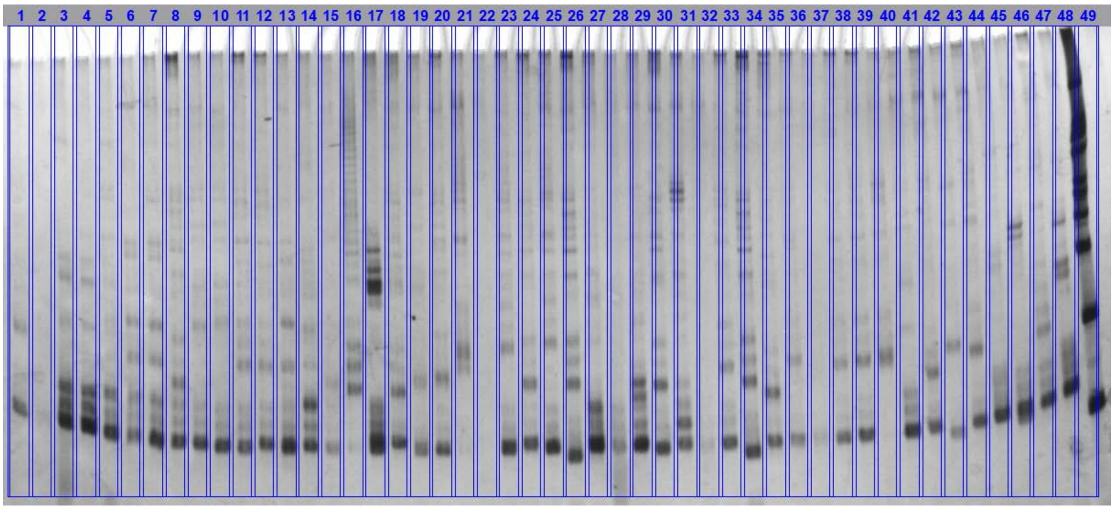
1 2 3 4 5 6 7 8 9 10 11 12 13 14 15 16 17 18 19 20 21 22 23 24 25 26 27 28 29 30 31 32 33 34 35 36 37 38 39 40 41 42 43 44 45 46 47 48 49

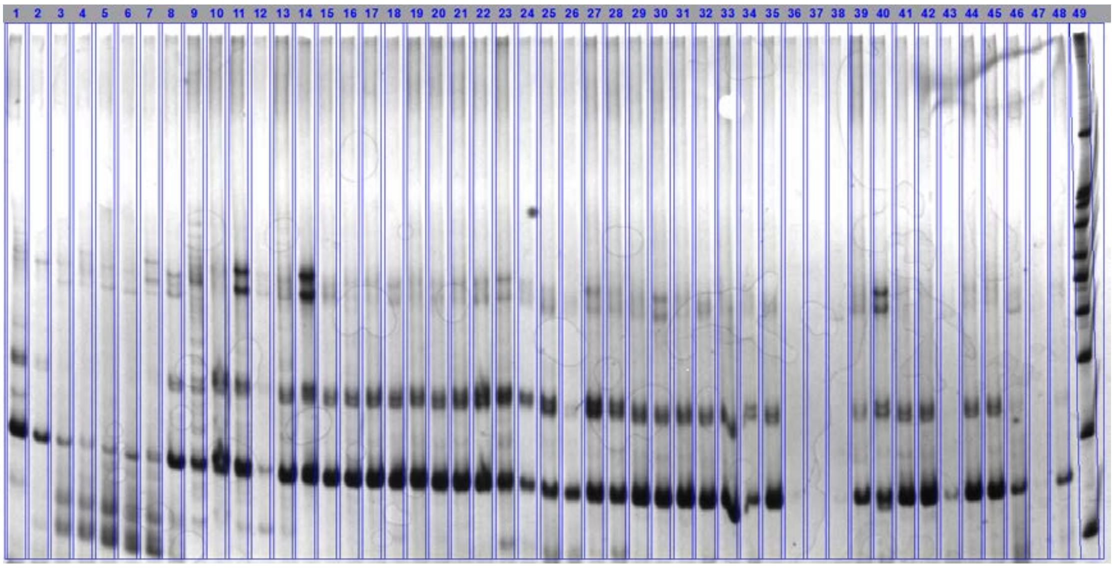
1 2 3 4 5 6 7 8 9 10 11 12 13 14 15 16 17 18 19 20 21 22 23 24 25 26 27 28 29 30 31 32 33 34 35 36 37 38 39 40 41 42 43 44 45 46 47 48 49

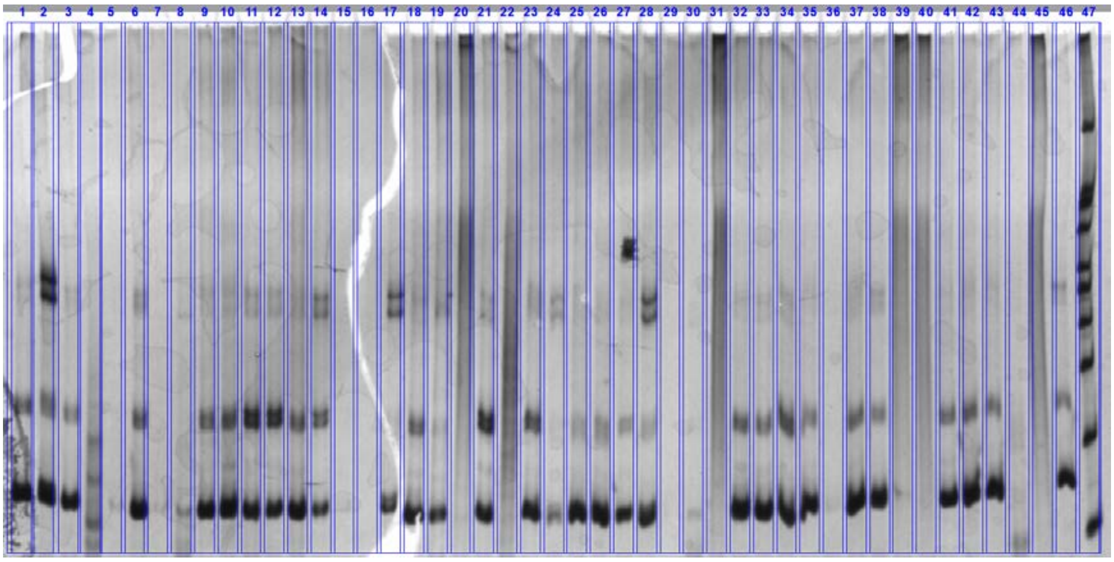
1 2 3 4 5 6 7 8 9 10 11 12 13 14 15 16 17 18 19 20 21 22 23 24 25 26 27 28 29 30 31 32 33 34 35 36 37 38 39 40 41 42 43 44 45 46 47

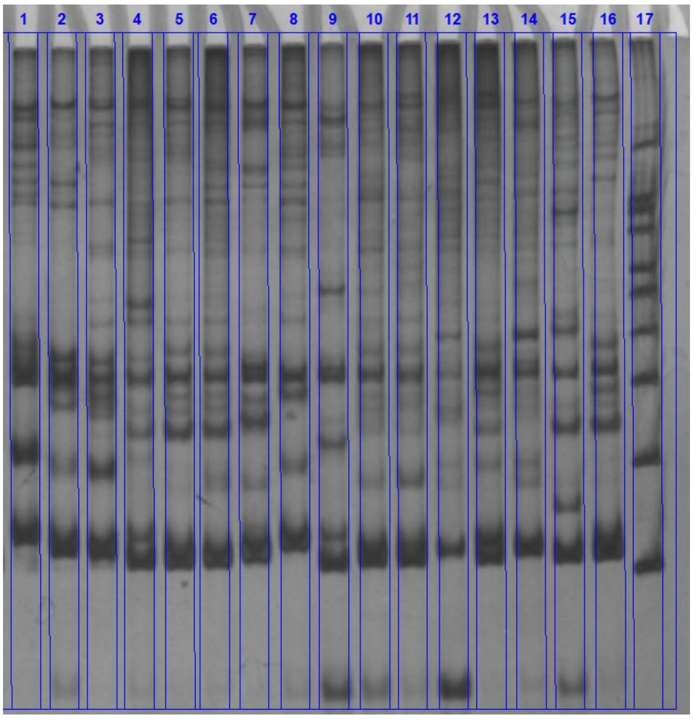

The gel images of primer X8H. The accessions from left to right in each gel image were 1, 2, 3, 4, 5, 6, 7, 8, 9, 10, 11, 13, 14, 16, 18, 19, 20, 21, 22, 23, 24, 25, 26, 27, 28, 29, 30, 31, 32, 33, 34, 35, 36, 37, 38, 39, 40, 41, 42, 43, 44, 45, 46, 47, 48, 49, 53, 55, 62, 71, 72, 81, 87, 89, 94, 99, 322, 324, 328, 329, 332, 334, 339, 341, 345, 350, 354, 358, 366,367, 368, 375, 381, 401, 404, 405, 406, 407, 408, 409, 421, 442, 443, 449, 455, 457, 459, 460, 462, 463, 464, 501, 502, 503, 504, 506, 507, 508, 509, 510, 511, 513, 516, 517, 518, 544, 558, 591, 592, 593, 594, 595, 596, 621, 622, 624, 626, 628, 754, 759, 771, 1001, 1002, 1003, 1004, 1005, 1006, BF, BX, BY01, BY02, BY03, JTY, KT, HW01, HW04, HW10, HW10, HW14, HW18, HW19, HW20, HL01, HL06, HL07, HL09, HL12, HL13, HL14, HZ03, HZ07, HZ09, HZ10, HZ11, HZ13, HZ14, XB.

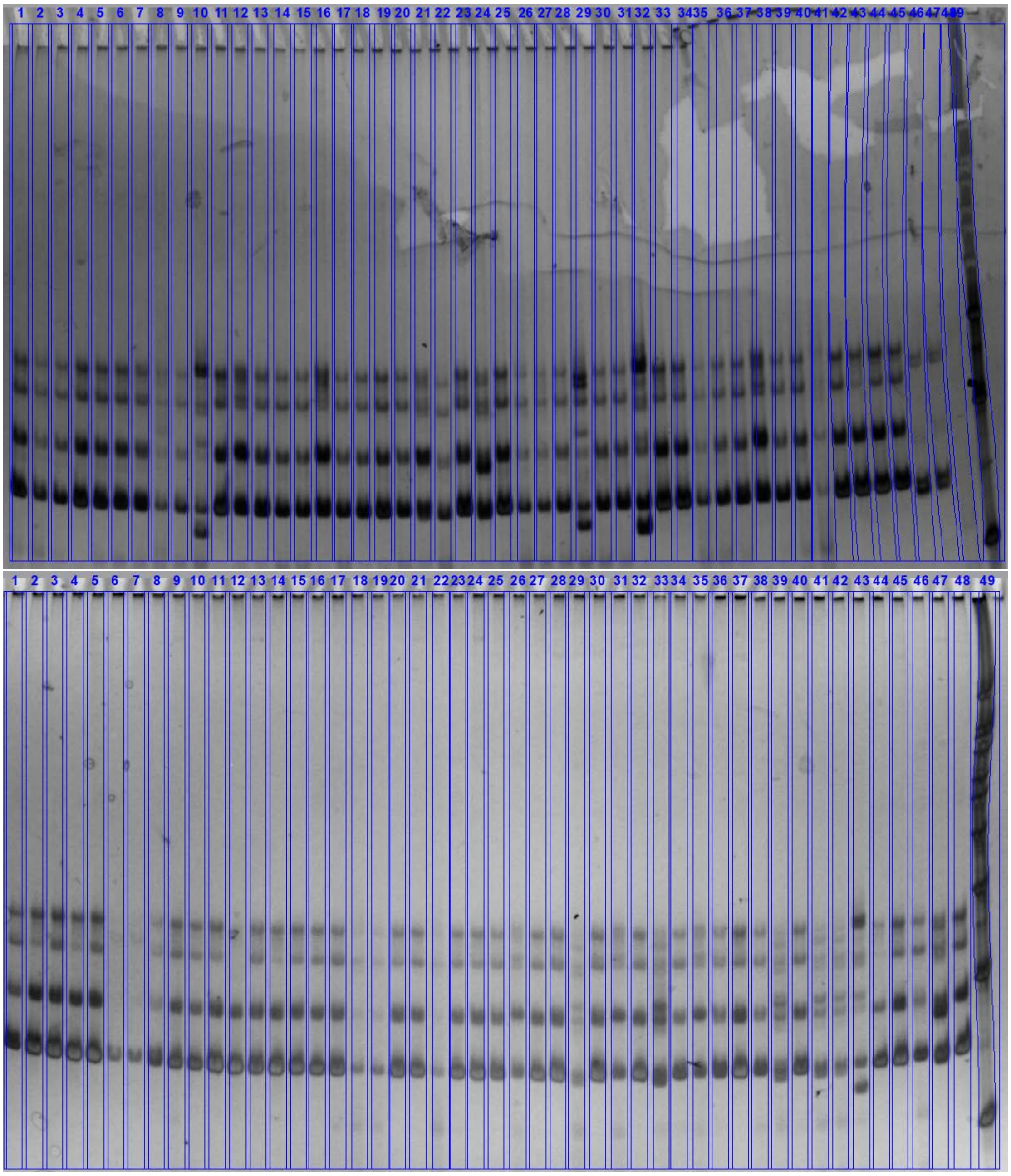

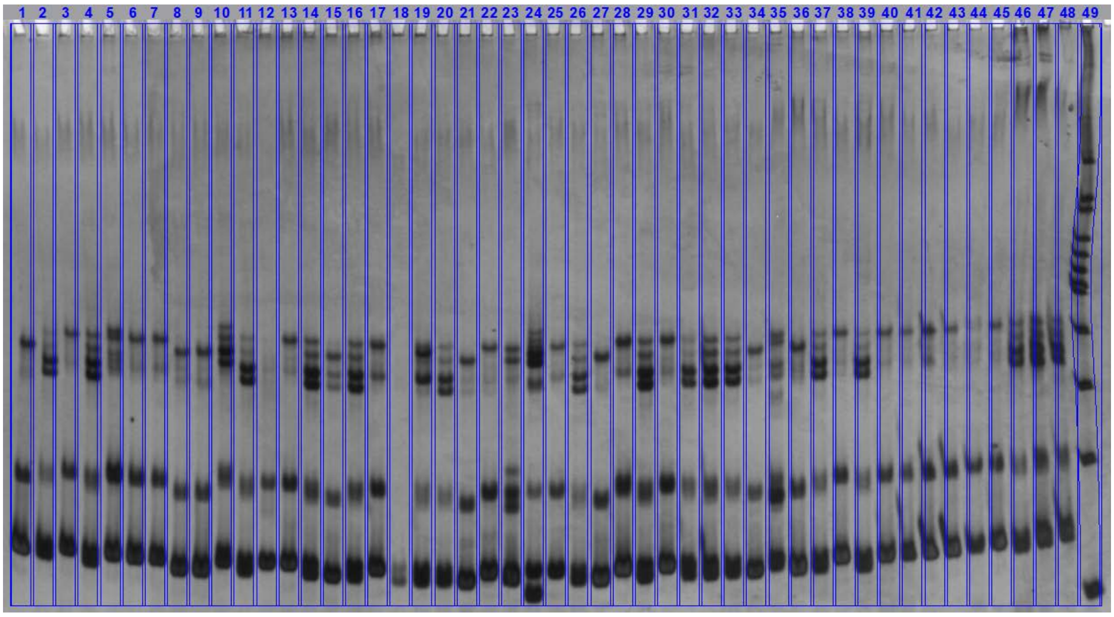

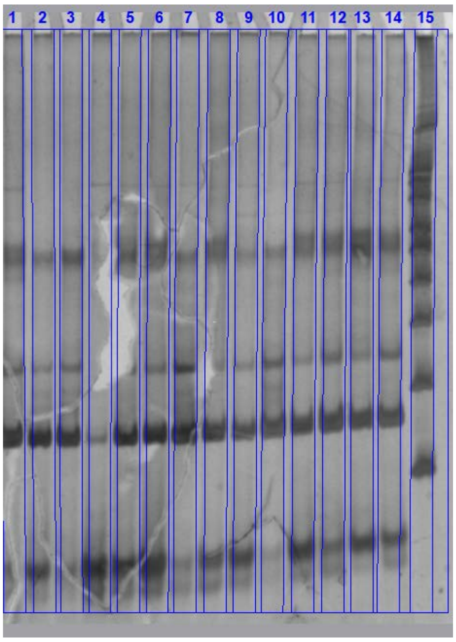

The gel images of primer X11H. The accessions from left to right in each gel image were 1, 2, 3, 4, 5, 6, 7, 8, 9, 10, 11, 13, 14, 16, 18, 19, 20, 21, 22, 23, 24, 25, 26, 27, 28, 29, 30, 31, 32, 33, 34, 35, 36, 37, 38, 39, 40, 41, 42, 43, 44, 45, 46, 47, 48, 49, 53, 55, 62, 71, 72, 81, 87, 89, 94, 99, 322, 324, 328, 329, 332, 334, 339, 341, 345, 350, 354, 358, 366,367, 368, 375, 381, 401, 404, 405, 406, 407, 408, 409, 421, 442, 443, 449, 455, 457, 459, 460, 462, 463, 464, 501, 502, 503, 504, 506, 507, 508, 509, 510, 511, 513, 516, 517, 518, 544, 558, 591, 592, 593, 594, 595, 596, 621, 622, 624, 626, 628, 754, 759, 771, 1001, 1002, 1003, 1004, 1005, 1006, BF, BX, BY01, BY02, BY03, JTY, KT, HW01, HW04, HW10, HW10, HW14, HW18, HW19, HW20, HL01, HL06, HL07, HL09, HL12, HL13, HL14, HZ03, HZ07, HZ09, HZ10, HZ11, HZ13, HZ14, XB.

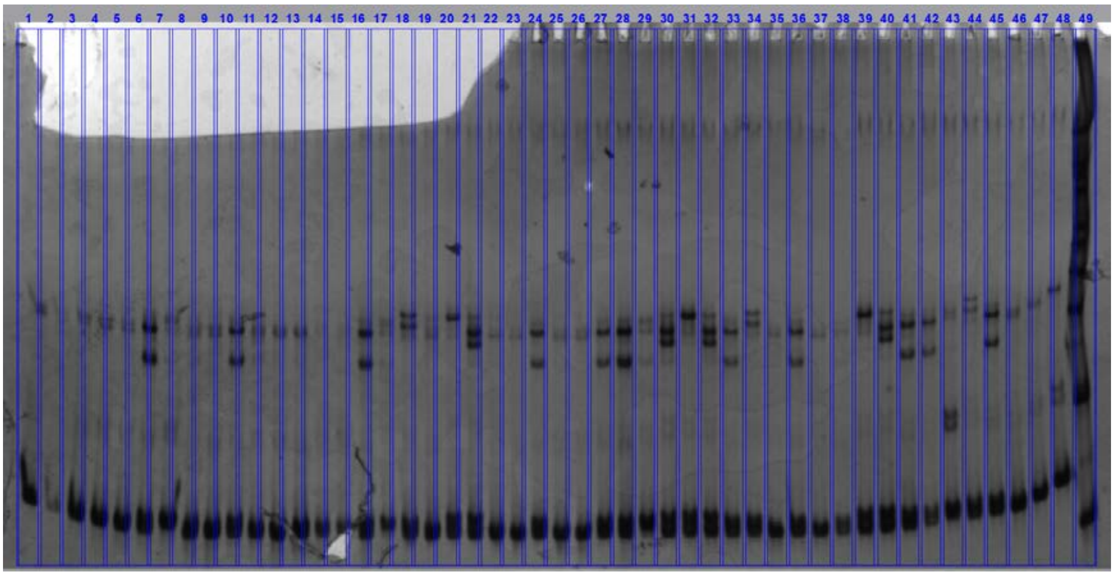
1 2 3 4 5 6 7 8 9 10 11 12 13 14 15 16 17 18 19 20 21 22 23 24 25 26 27 28 29 30 31 32 33 34 35 36 37 38 39 40 41 42 43 44 45 46 47 48 49

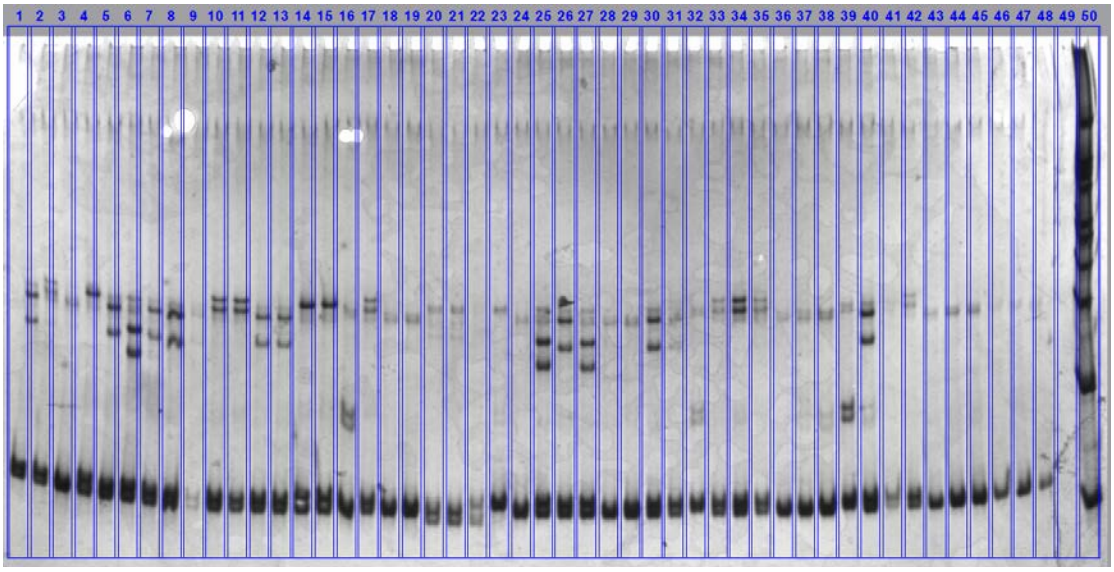
1 2 3 4 5 6 7 8 9 10 11 12 13 14 15 16 17 18 19 20 21 22 23 24 25 26 27 28 29 30 31 32 33 34 35 36 37 38 39 40 41 42 43 44 45 46 47 48 49 50

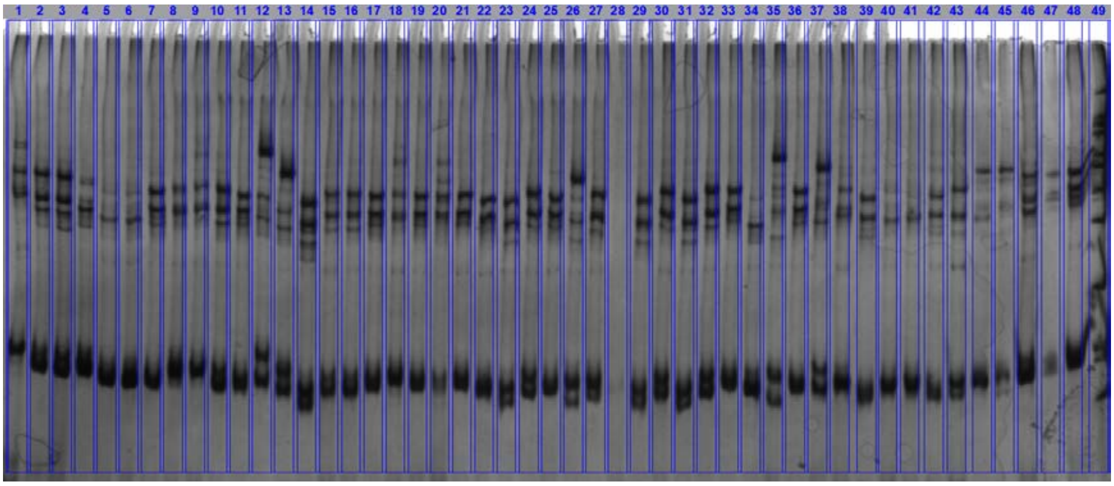
1 2 3 4 5 6 7 8 9 10 11 12 13 14 15 16 17 18 19 20 21 22 23 24 25 26 27 28 29 30 31 32 33 34 35 36 37 38 39 40 41 42 43 44 45 46 47 48 49

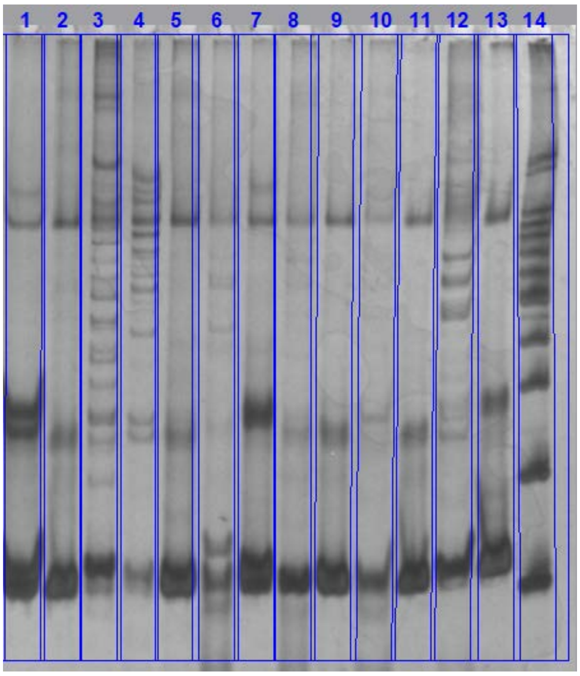

The gel images of primer X15H. The accessions from left to right in each gel image were 1, 2, 3, 4, 5, 6, 7, 8, 9, 10, 11, 13, 14, 16, 18, 19, 20, 21, 22, 23, 24, 25, 26, 27, 28, 29, 30, 31, 32, 33, 34, 35, 36, 37, 38, 39, 40, 41, 42, 43, 44, 45, 46, 47, 48, 49, 53, 55, 62, 71, 72, 81, 87, 89, 94, 99, 322, 324, 328, 329, 332, 334, 339, 341, 345, 350, 354, 358, 366,367, 368, 375, 381, 401, 404, 405, 406, 407, 408, 409, 421, 442, 443, 449, 455, 457, 459, 460, 462, 463, 464, 501, 502, 503, 504, 506, 507, 508, 509, 510, 511, 513, 516, 517, 518, 544, 558, 591, 592, 593, 594, 595, 596, 621, 622, 624, 626, 628, 754, 759, 771, 1001, 1002, 1003, 1004, 1005, 1006, BF, BX, BY01, BY02, BY03, JTY, KT, HW01, HW04, HW10, HW10, HW14, HW18, HW19, HW20, HL01, HL06, HL07, HL09, HL12, HL13, HL14, HZ03, HZ07, HZ09, HZ10, HZ11, HZ13, HZ14, XB.

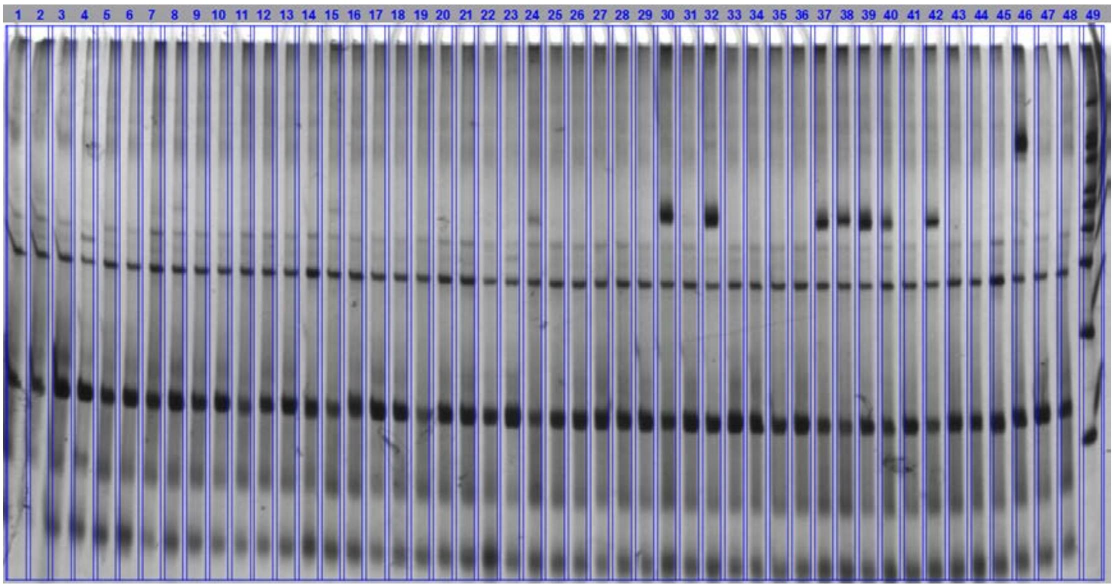
1 2 3 4 5 6 7 8 9 10 11 12 13 14 15 16 17 18 19 20 21 22 23 24 25 26 27 28 29 30 31 32 33 34 35 36 37 38 39 40 41 42 43 44 45 46 47 48 49

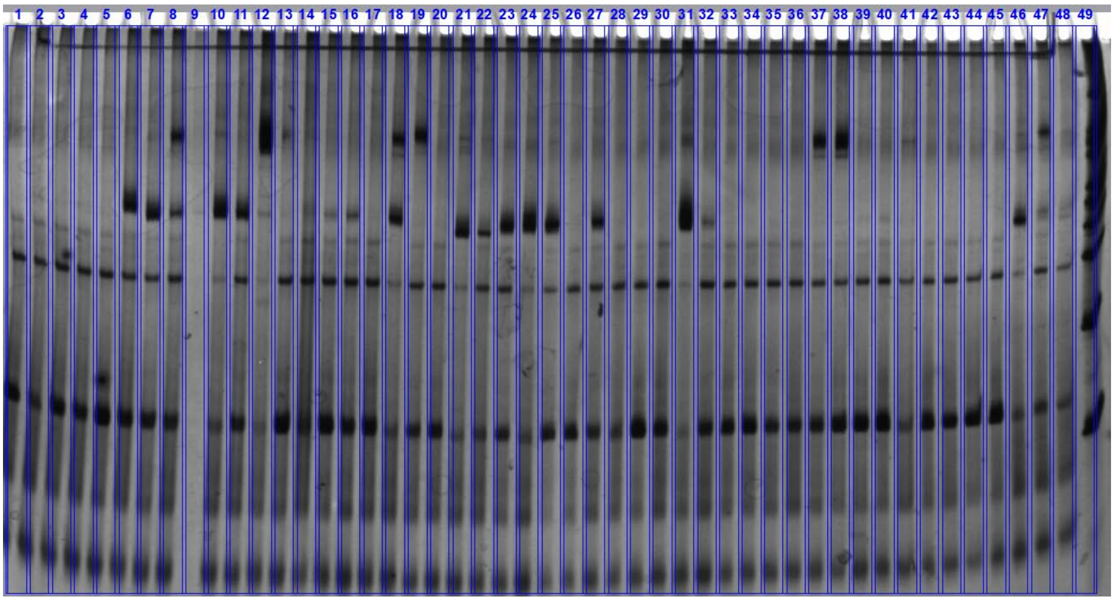
1 2 3 4 5 6 7 8 9 10 11 12 13 14 15 16 17 18 19 20 21 22 23 24 25 26 27 28 29 30 31 32 33 34 35 36 37 38 39 40 41 42 43 44 45 46 47 48 49

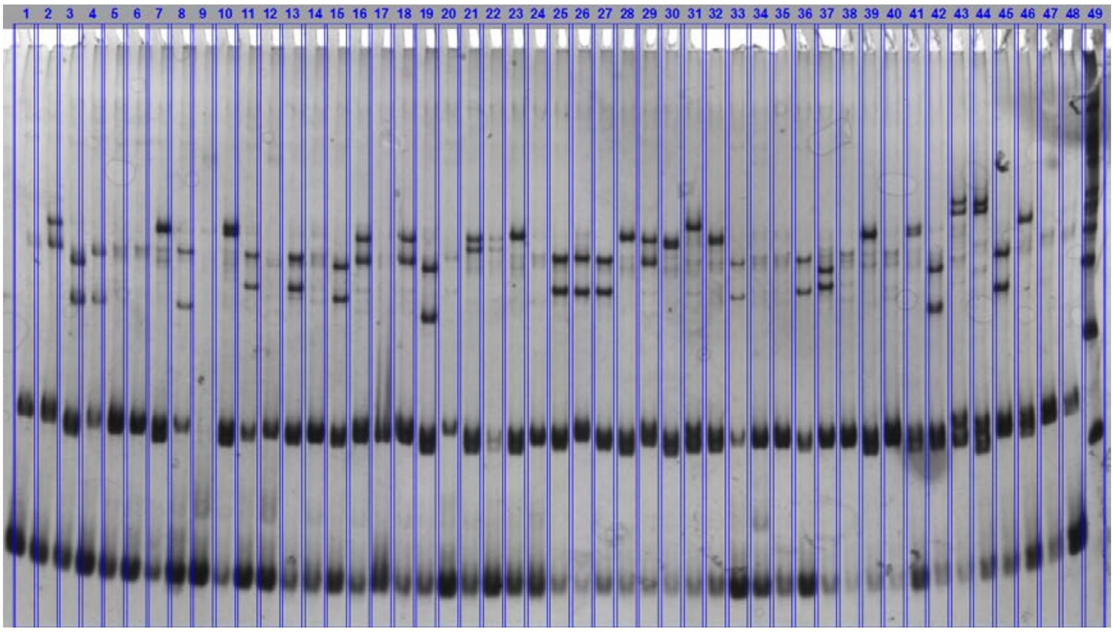
1 2 3 4 5 6 7 8 9 10 11 12 13 14 15 16 17 18 19 20 21 22 23 24 25 26 27 28 29 30 31 32 33 34 35 36 37 38 39 40 41 42 43 44 45 46 47 48 49

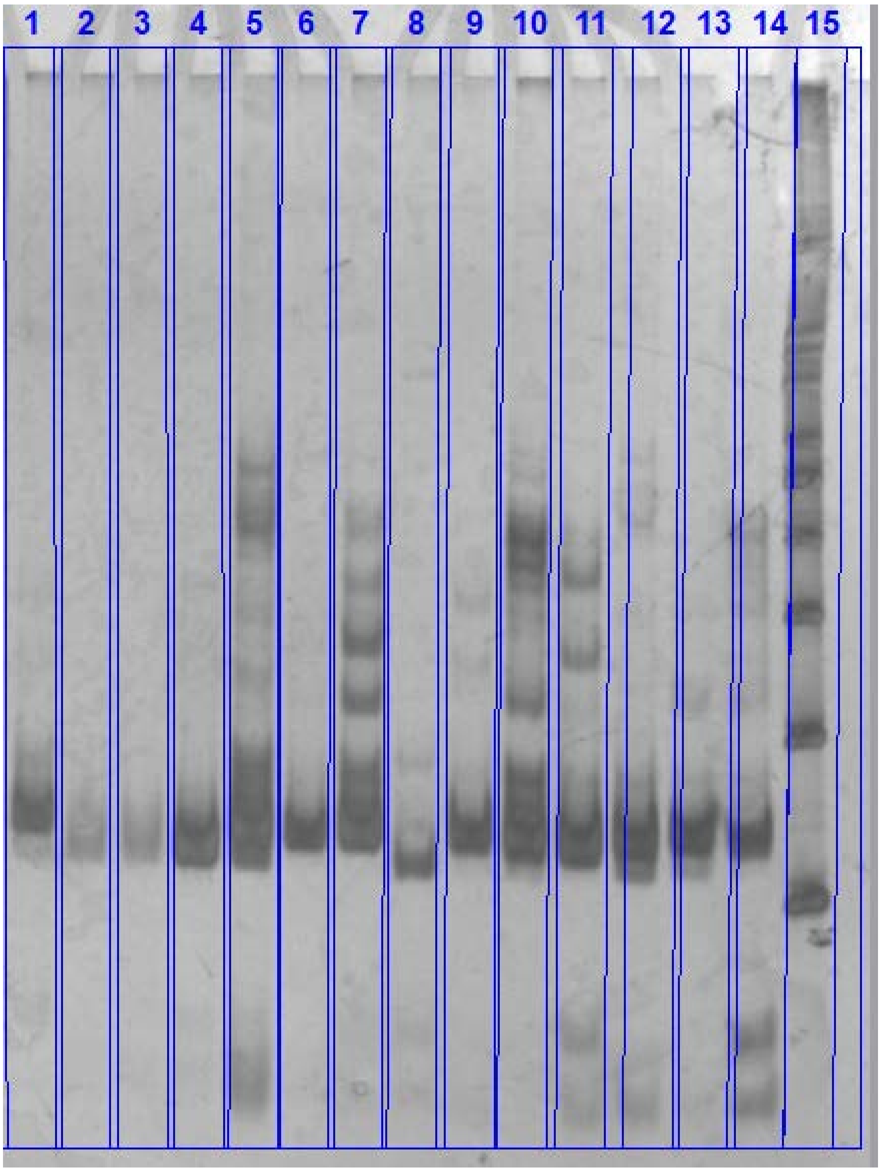

The gel images of primer X19H. The accessions from left to right in each gel image were 1, 2, 3, 4, 5, 6, 7, 8, 9, 10, 11, 13, 14, 16, 18, 19, 20, 21, 22, 23, 24, 25, 26, 27, 28, 29, 30, 31, 32, 33, 34, 35, 36, 37, 38, 39, 40, 41, 42, 43, 44, 45, 46, 47, 48, 49, 53, 55, 62, 71, 72, 81, 87, 89, 94, 99, 322, 324, 328, 329, 332, 334, 339, 341, 345, 350, 354, 358, 366,367, 368, 375, 381, 401, 404, 405, 406, 407, 408, 409, 421, 442, 443, 449, 455, 457, 459, 460, 462, 463, 464, 501, 502, 503, 504, 506, 507, 508, 509, 510, 511, 513, 516, 517, 518, 544, 558, 591, 592, 593, 594, 595, 596, 621, 622, 624, 626, 628, 754, 759, 771, 1001, 1002, 1003, 1004, 1005, 1006, BF, BX, BY01, BY02, BY03, JTY, KT, HW01, HW04, HW10, HW10, HW14, HW18, HW19, HW20, HL01, HL06, HL07, HL09, HL12, HL13, HL14, HZ03, HZ07, HZ09, HZ10, HZ11, HZ13, HZ14, XB.

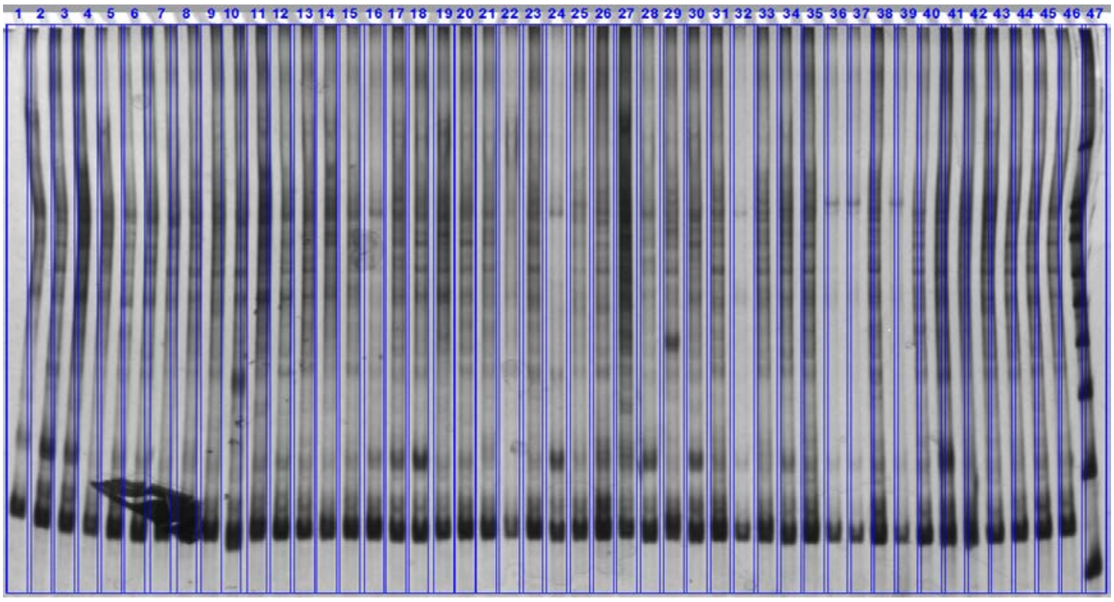
1 2 3 4 5 6 7 8 9 10 11 12 13 14 15 16 17 18 19 20 21 22 23 24 25 26 27 28 29 30 31 32 33 34 35 36 37 38 39 40 41 42 43 44 45 46 47

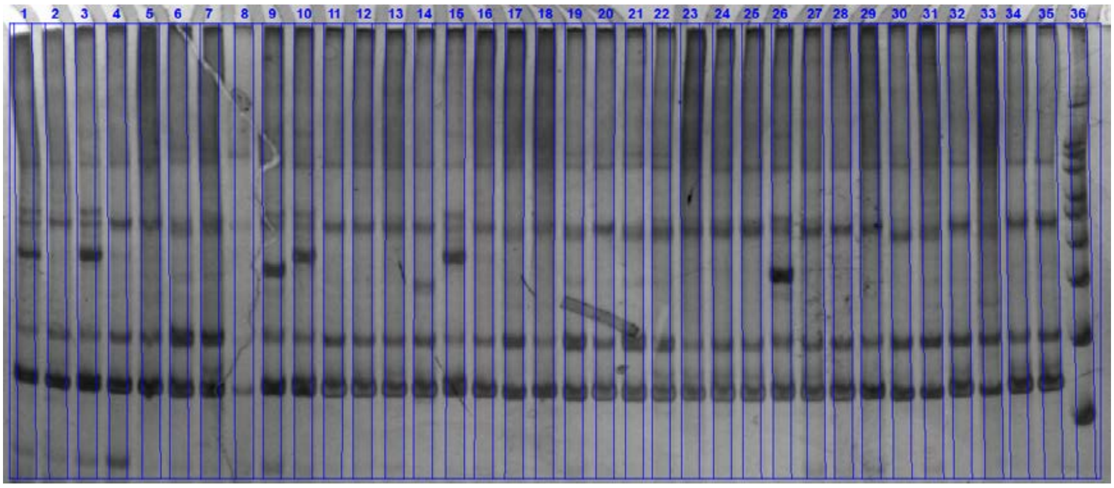
1 2 3 4 5 6 7 8 9 10 11 12 13 14 15 16 17 18 19 20 21 22 23 24 25 26 27 28 29 30 31 32 33 34 35 36

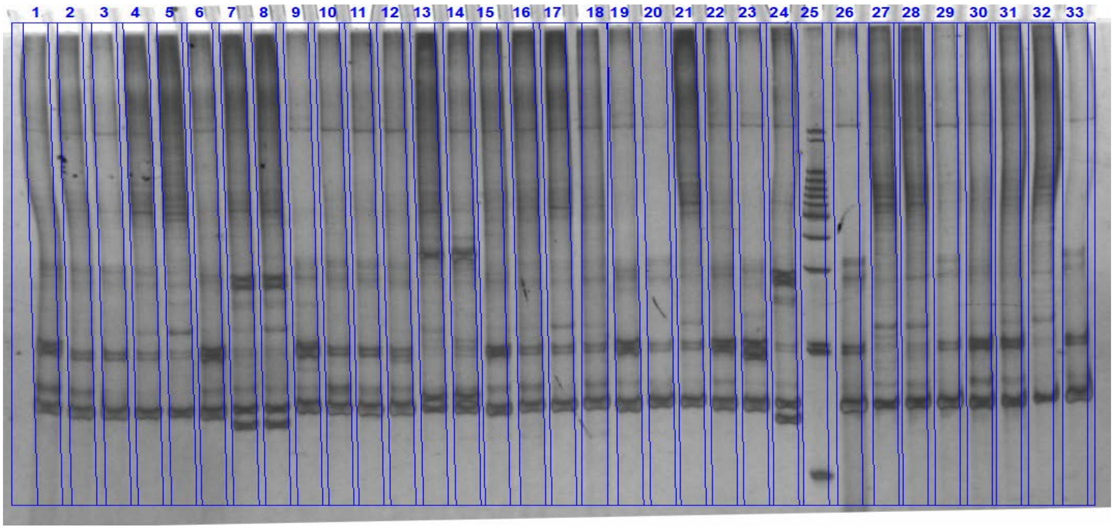
1 2 3 4 5 6 7 8 9 10 11 12 13 14 15 16 17 18 19 20 21 22 23 24 25 26 27 28 29 30 31 32 33

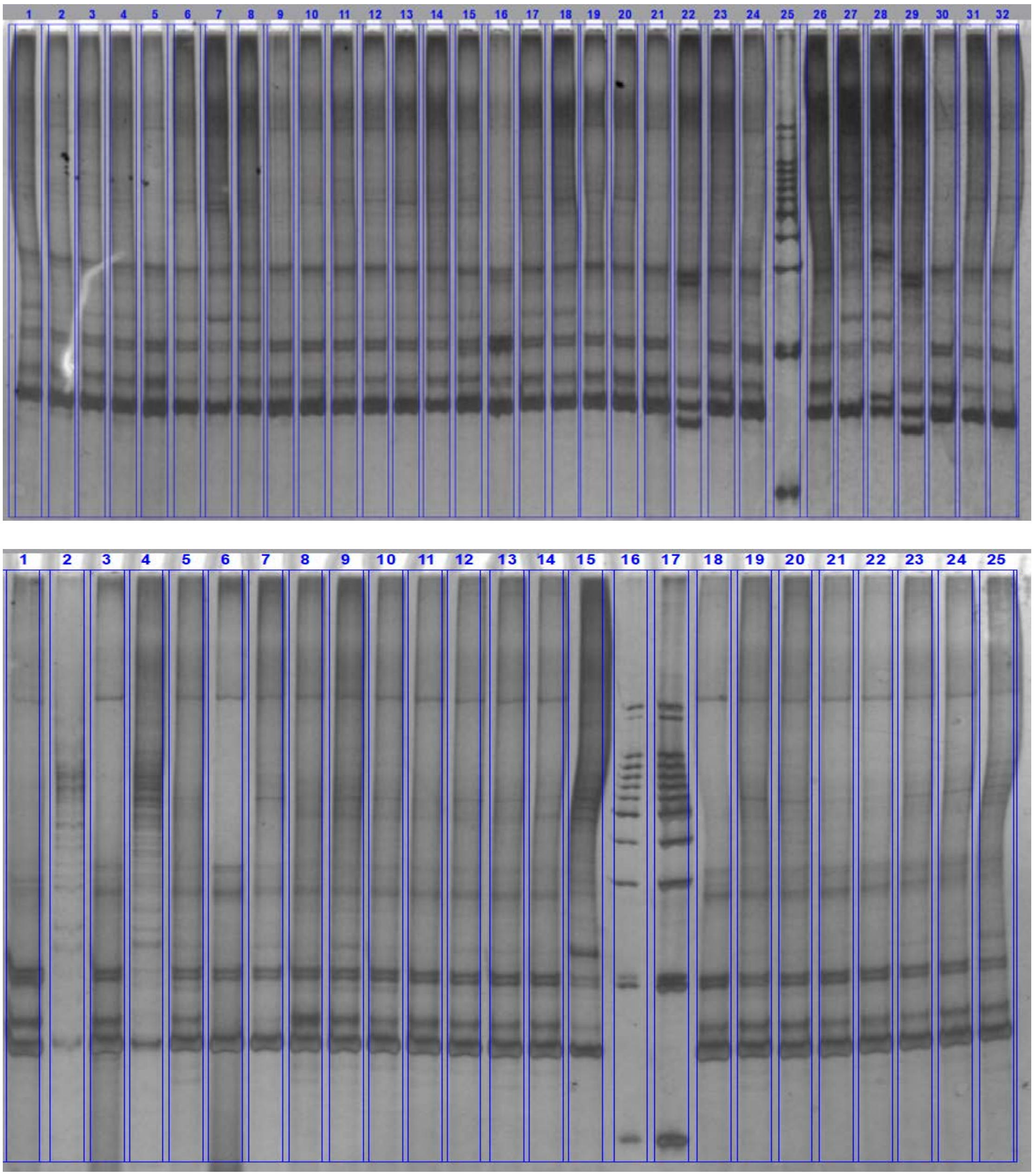

The gel images of primer X32H. The accessions from left to right in each gel image were 1, 2, 3, 4, 5, 6, 7, 8, 9, 10, 11, 13, 14, 16, 18, 19, 20, 21, 22, 23, 24, 25, 26, 27, 28, 29, 30, 31, 32, 33, 34, 35, 36, 37, 38, 39, 40, 41, 42, 43, 44, 45, 46, 47, 48, 49, 53, 55, 62, 71, 72, 81, 87, 89, 94, 99, 322, 324, 328, 329, 332, 334, 339, 341, 345, 350, 354, 358, 366,367, 368, 375, 381, 401, 404, 405, 406, 407, 408, 409, 421, 442, 443, 449, 455, 457, 459, 460, 462, 463, 464, 501, 502, 503, 504, 506, 507, 508, 509, 510, 511, 513, 516, 517, 518, 544, 558, 591, 592, 593, 594, 595, 596, 621, 622, 624, 626, 628, 754, 759, 771, 1001, 1002, 1003, 1004, 1005, 1006, BF, BX, BY01, BY02, BY03, JTY, KT, HW01, HW04, HW10, HW10, HW14, HW18, HW19, HW20, HL01, HL06, HL07, HL09, HL12, HL13, HL14, HZ03, HZ07, HZ09, HZ10, HZ11, HZ13, HZ14, XB.

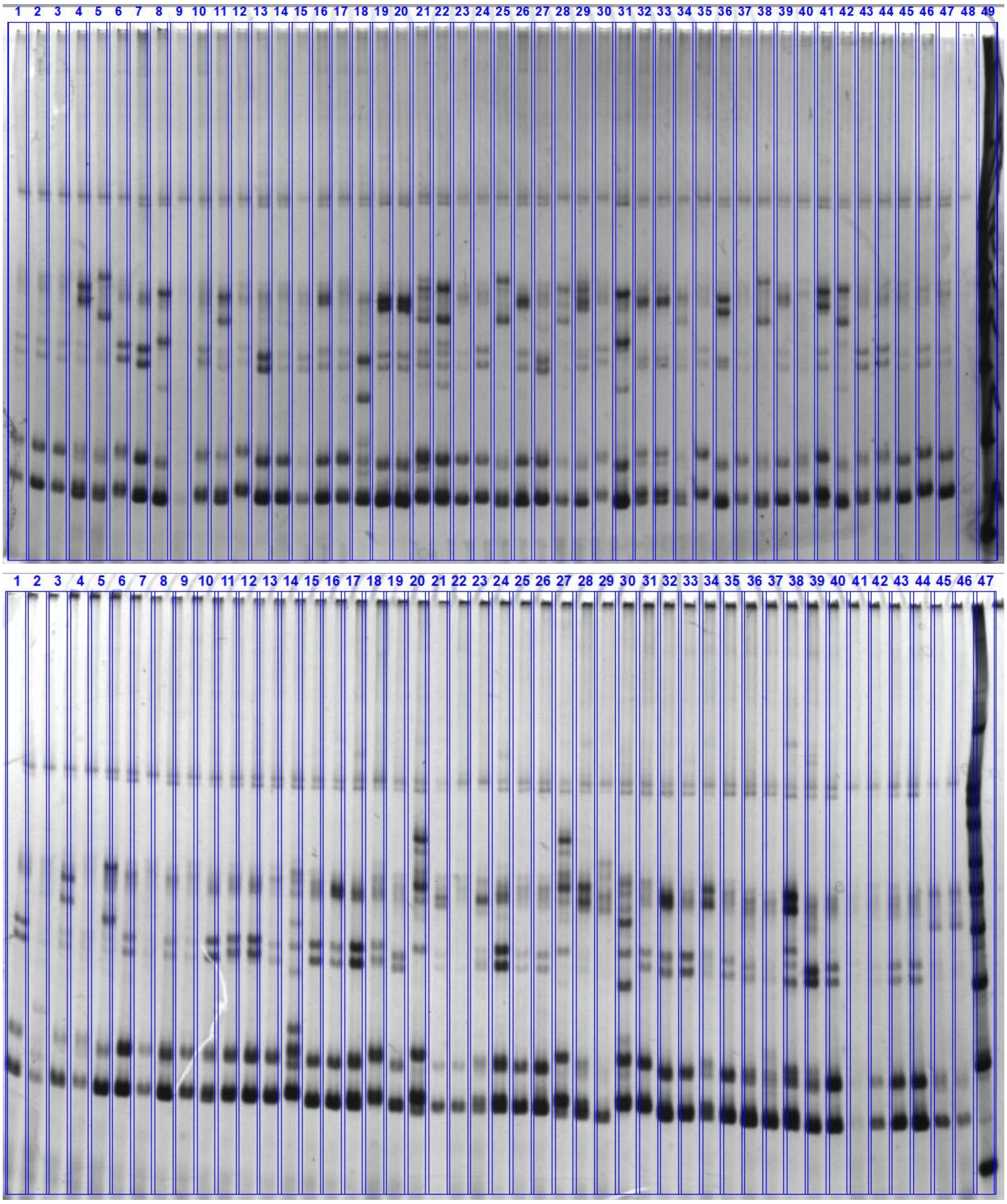
1 2 3 4 5 6 7 8 9 10 11 12 13 14 15 16 17 18 19 20 21 22 23 24 25 26 27 28 29 30 31 32 33 34 35 36 37 38 39 40 41 42 43 44 45 46 47 48 49
1 2 3 4 5 6 7 8 9 10 11 12 13 14 15 16 17 18 19 20 21 22 23 24 25 26 27 28 29 30 31 32 33 34 35 36 37 38 39 40 41 42 43 44 45 46 47

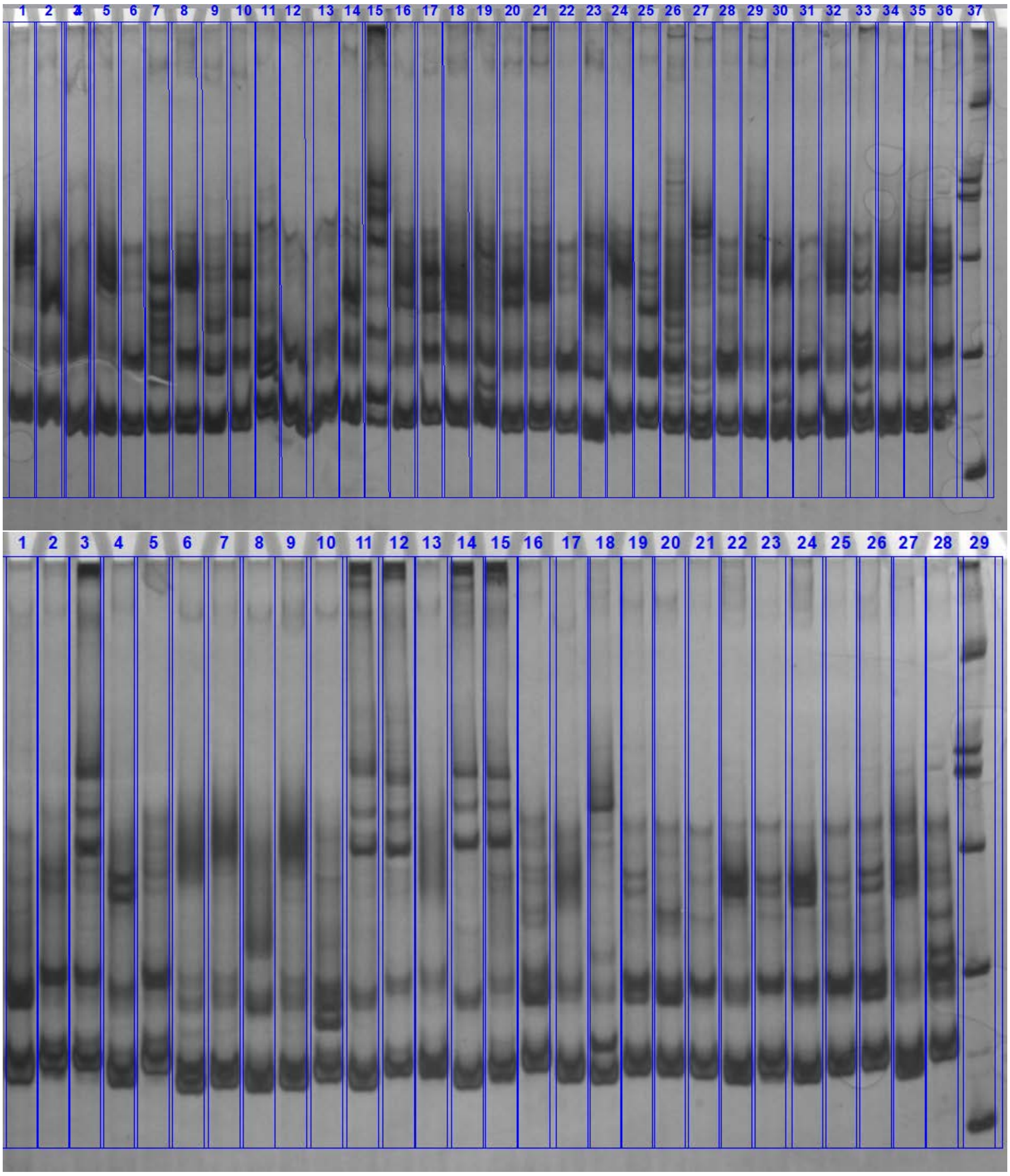

The gel images of primer X38H. The accessions from left to right in each gel image were 1, 2, 3, 4, 5, 6, 7, 8, 9, 10, 11, 13, 14, 16, 18, 19, 20, 21, 22, 23, 24, 25, 26, 27, 28, 29, 30, 31, 32, 33, 34, 35, 36, 37, 38, 39, 40, 41, 42, 43, 44, 45, 46, 47, 48, 49, 53, 55, 62, 71, 72, 81, 87, 89, 94, 99, 322, 324, 328, 329, 332, 334, 339, 341, 345, 350, 354, 358, 366,367, 368, 375, 381, 401, 404, 405, 406, 407, 408, 409, 421, 442, 443, 449, 455, 457, 459, 460, 462, 463, 464, 501, 502, 503, 504, 506, 507, 508, 509, 510, 511, 513, 516, 517, 518, 544, 558, 591, 592, 593, 594, 595, 596, 621, 622, 624, 626, 628, 754, 759, 771, 1001, 1002, 1003, 1004, 1005, 1006, BF, BX, BY01, BY02, BY03, JTY, KT, HW01, HW04, HW10, HW10, HW14, HW18, HW19, HW20, HL01, HL06, HL07, HL09, HL12, HL13, HL14, HZ03, HZ07, HZ09, HZ10, HZ11, HZ13, HZ14, XB.

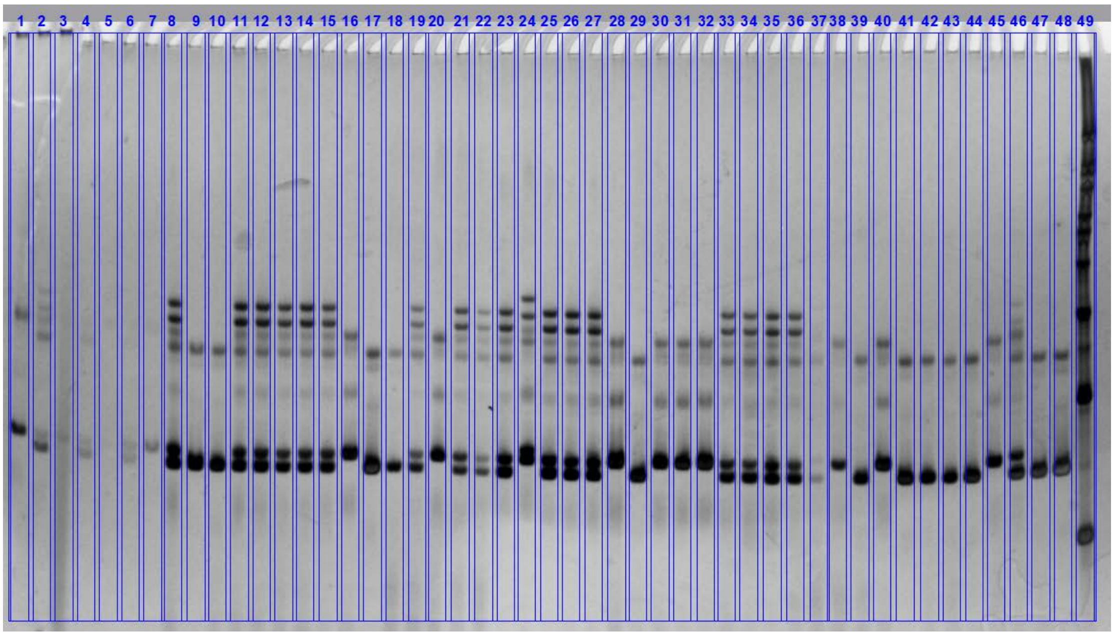
1 2 3 4 5 6 7 8 9 10 11 12 13 14 15 16 17 18 19 20 21 22 23 24 25 26 27 28 29 30 31 32 33 34 35 36 37 38 39 40 41 42 43 44 45 46 47 48 49

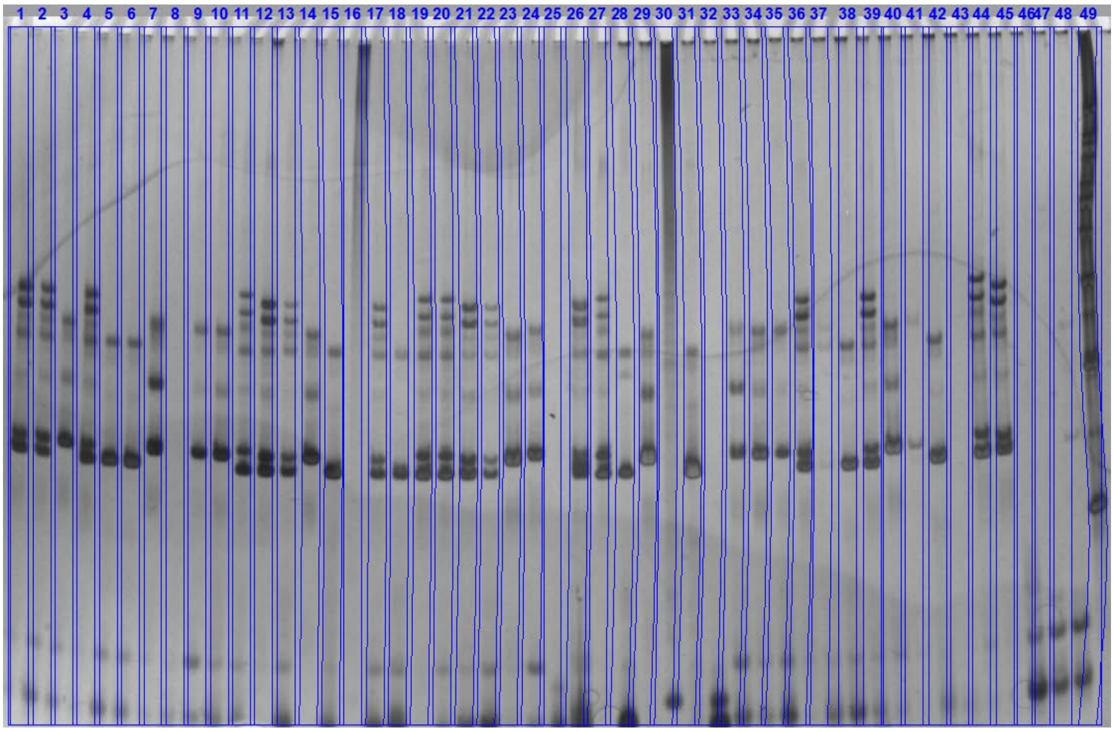
1 2 3 4 5 6 7 8 9 10 11 12 13 14 15 16 17 18 19 20 21 22 23 24 25 26 27 28 29 30 31 32 33 34 35 36 37 38 39 40 41 42 43 44 45 46 47 48 49

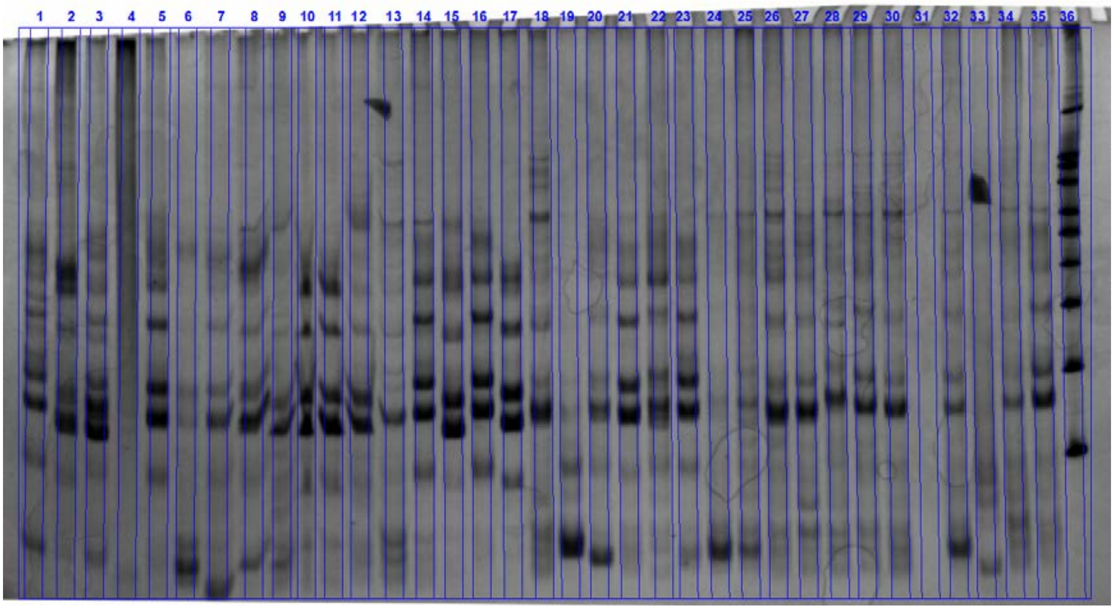

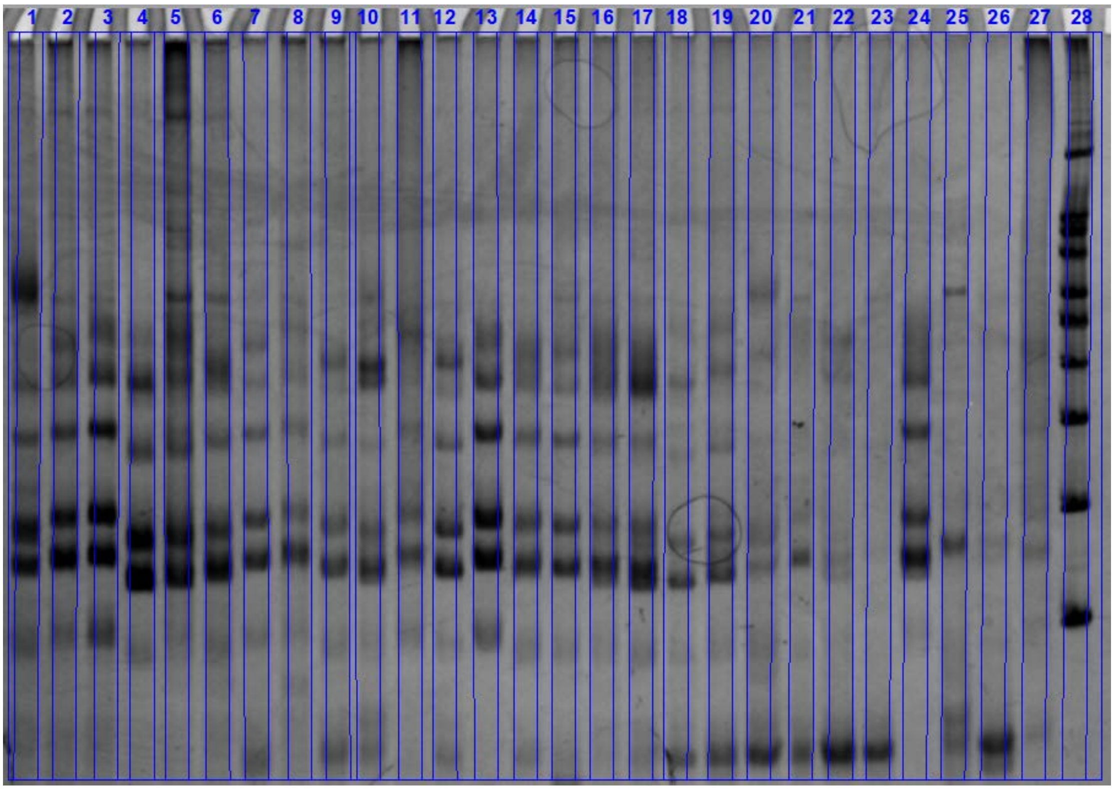

The gel images of primer X42H. The accessions from left to right in each gel image were 1, 2, 3, 4, 5, 6, 7, 8, 9, 10, 11, 13, 14, 16, 18, 19, 20, 21, 22, 23, 24, 25, 26, 27, 28, 29, 30, 31, 32, 33, 34, 35, 36, 37, 38, 39, 40, 41, 42, 43, 44, 45, 46, 47, 48, 49, 53, 55, 62, 71, 72, 81, 87, 89, 94, 99, 322, 324, 328, 329, 332, 334, 339, 341, 345, 350, 354, 358, 366,367, 368, 375, 381, 401, 404, 405, 406, 407, 408, 409, 421, 442, 443, 449, 455, 457, 459, 460, 462, 463, 464, 501, 502, 503, 504, 506, 507, 508, 509, 510, 511, 513, 516, 517, 518, 544, 558, 591, 592, 593, 594, 595, 596, 621, 622, 624, 626, 628, 754, 759, 771, 1001, 1002, 1003, 1004, 1005, 1006, BF, BX, BY01, BY02, BY03, JTY, KT, HW01, HW04, HW10, HW10, HW14, HW18, HW19, HW20, HL01, HL06, HL07, HL09, HL12, HL13, HL14, HZ03, HZ07, HZ09, HZ10, HZ11, HZ13, HZ14, XB.

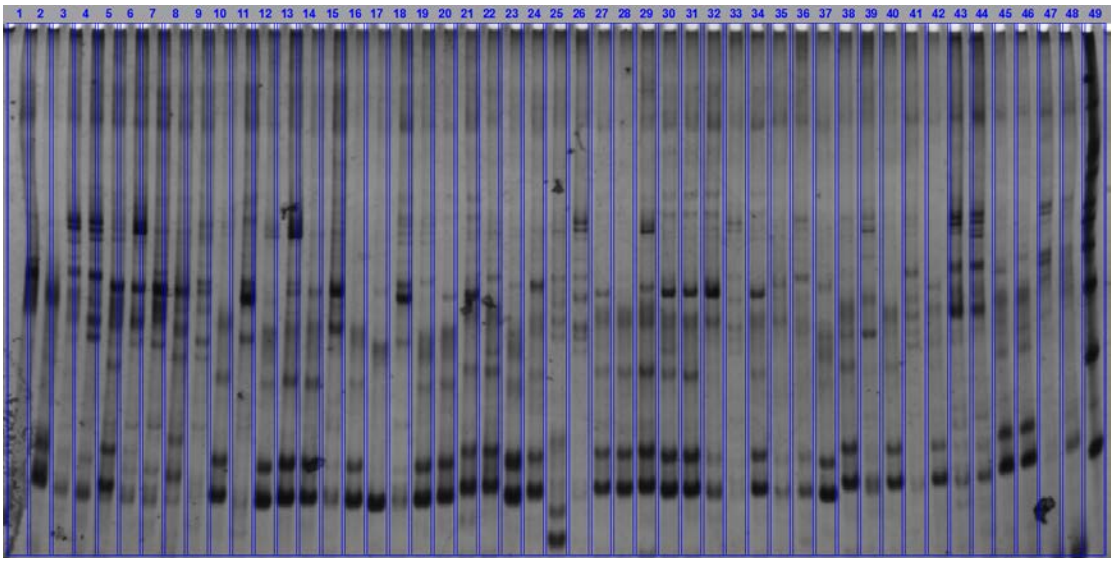
1 2 3 4 5 6 7 8 9 10 11 12 13 14 15 16 17 18 19 20 21 22 23 24 25 26 27 28 29 30 31 32 33 34 35 36 37 38 39 40 41 42 43 44 45 46 47 48 49

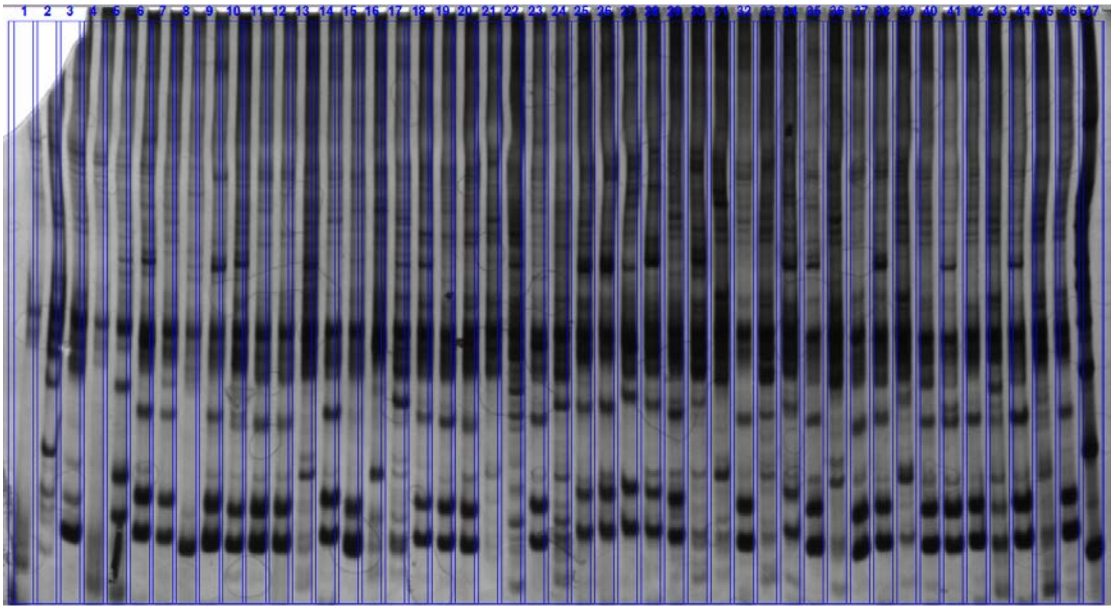

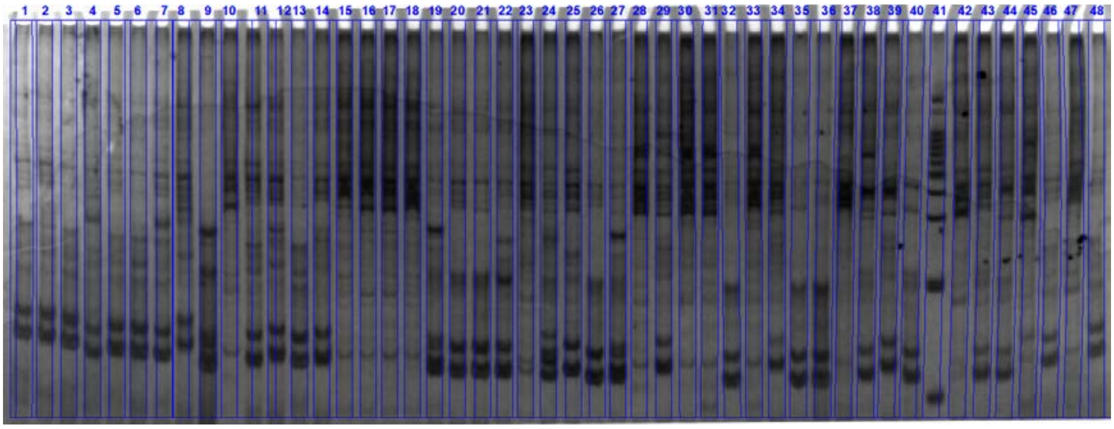
1 2 3 4 5 6 7 8 9 10 11 12 13 14 15 16 17 18 19 20 21 22 23 24 25 26 27 28 29 30 31 32 33 34 35 36 37 38 39 40 41 42 43 44 45 46 47 48

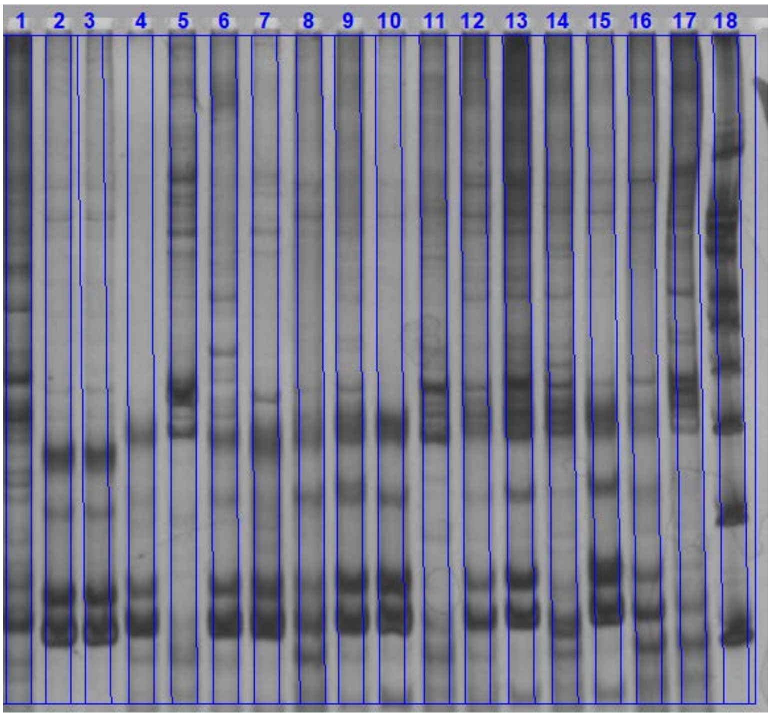

The gel images of primer X44H. The accessions from left to right in each gel image were 1, 2, 3, 4, 5, 6, 7, 8, 9, 10, 11, 13, 14, 16, 18, 19, 20, 21, 22, 23, 24, 25, 26, 27, 28, 29, 30, 31, 32, 33, 34, 35, 36, 37, 38, 39, 40, 41, 42, 43, 44, 45, 46, 47, 48, 49, 53, 55, 62, 71, 72, 81, 87, 89, 94, 99, 322, 324, 328, 329, 332, 334, 339, 341, 345, 350, 354, 358, 366,367, 368, 375, 381, 401, 404, 405, 406, 407, 408, 409, 421, 442, 443, 449, 455, 457, 459, 460, 462, 463, 464, 501, 502, 503, 504, 506, 507, 508, 509, 510, 511, 513, 516, 517, 518, 544, 558, 591, 592, 593, 594, 595, 596, 621, 622, 624, 626, 628, 754, 759, 771, 1001, 1002, 1003, 1004, 1005, 1006, BF, BX, BY01, BY02, BY03, JTY, KT, HW01, HW04, HW10, HW10, HW14, HW18, HW19, HW20, HL01, HL06, HL07, HL09, HL12, HL13, HL14, HZ03, HZ07, HZ09, HZ10, HZ11, HZ13, HZ14, XB.

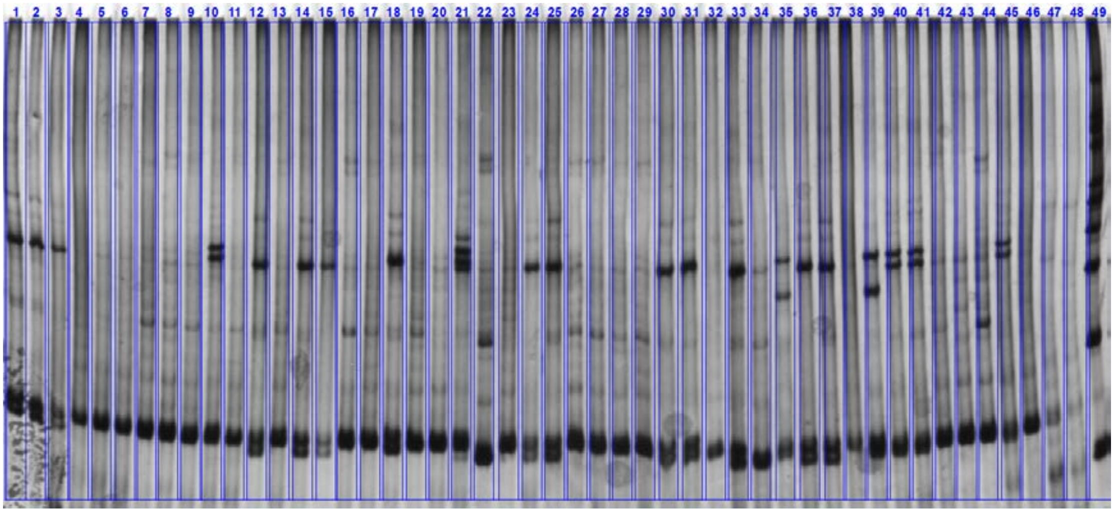
1 2 3 4 5 6 7 8 9 10 11 12 13 14 15 16 17 18 19 20 21 22 23 24 25 26 27 28 29 30 31 32 33 34 35 36 37 38 39 40 41 42 43 44 45 46 47 48 49

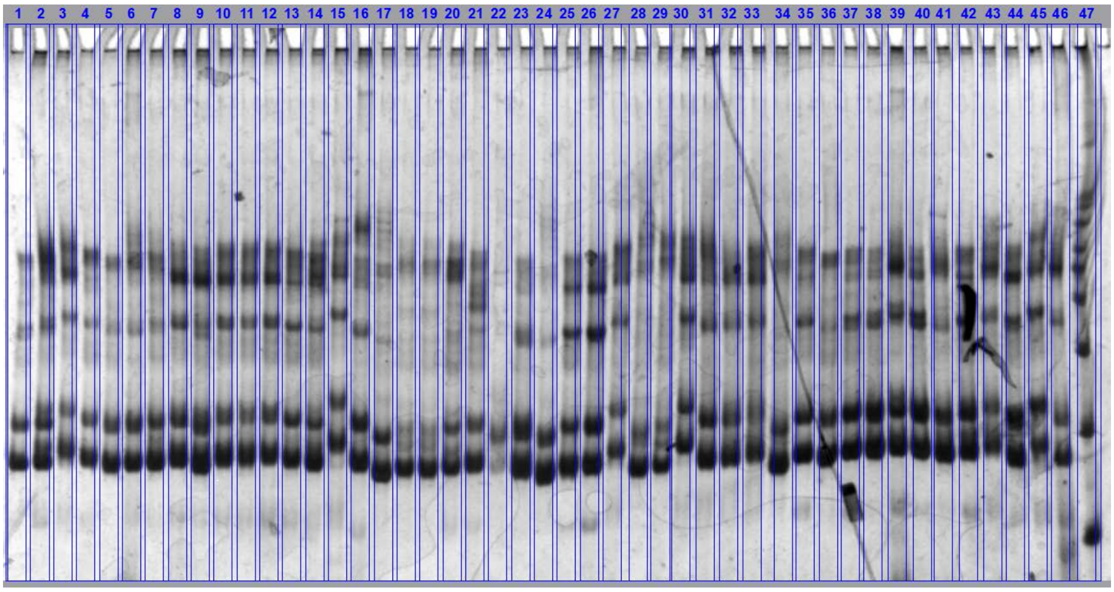
1 2 3 4 5 6 7 8 9 10 11 12 13 14 15 16 17 18 19 20 21 22 23 24 25 26 27 28 29 30 31 32 33 34 35 36 37 38 39 40 41 42 43 44 45 46 47

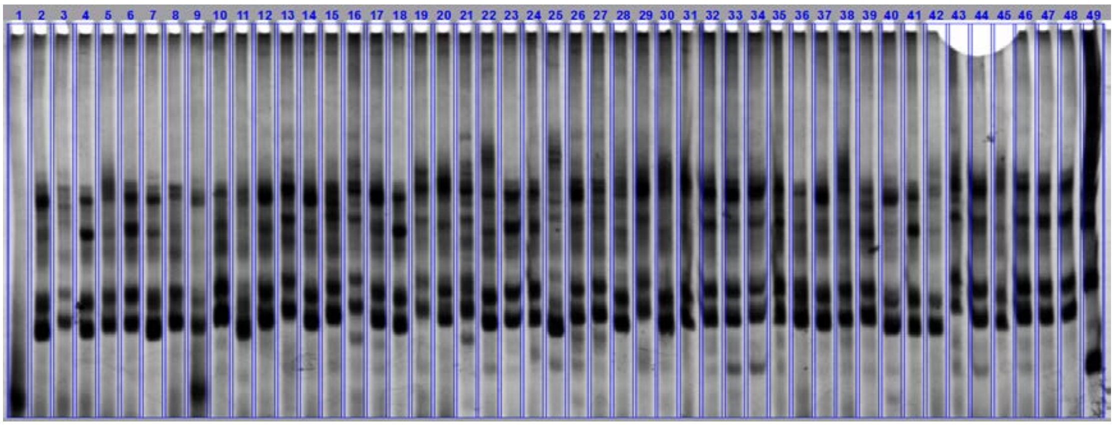
1 2 3 4 5 6 7 8 9 10 11 12 13 14 15 16 17 18 19 20 21 22 23 24 25 26 27 28 29 30 31 32 33 34 35 36 37 38 39 40 41 42 43 44 45 46 47 48 49

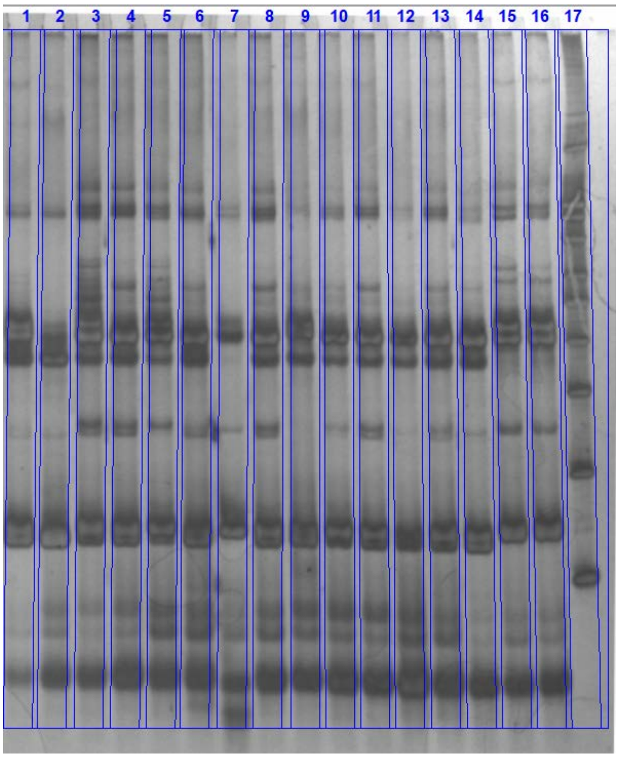

The gel images of primer X47. The accessions from left to right in each gel image were 1, 2, 3, 4, 5, 6, 7, 8, 9, 10, 11, 13, 14, 16, 18, 19, 20, 21, 22, 23, 24, 25, 26, 27, 28, 29, 30, 31, 32, 33, 34, 35, 36, 37, 38, 39, 40, 41, 42, 43, 44, 45, 46, 47, 48, 49, 53, 55, 62, 71, 72, 81, 87, 89, 94, 99, 322, 324, 328, 329, 332, 334, 339, 341, 345, 350, 354, 358, 366,367, 368, 375, 381, 401, 404, 405, 406, 407, 408, 409, 421, 442, 443, 449, 455, 457, 459, 460, 462, 463, 464, 501, 502, 503, 504, 506, 507, 508, 509, 510, 511, 513, 516, 517, 518, 544, 558, 591, 592, 593, 594, 595, 596, 621, 622, 624, 626, 628, 754, 759, 771, 1001, 1002, 1003, 1004, 1005, 1006, BF, BX, BY01, BY02, BY03, JTY, KT, HW01, HW04, HW10, HW10, HW14, HW18, HW19, HW20, HL01, HL06, HL07, HL09, HL12, HL13, HL14, HZ03, HZ07, HZ09, HZ10, HZ11, HZ13, HZ14, XB.

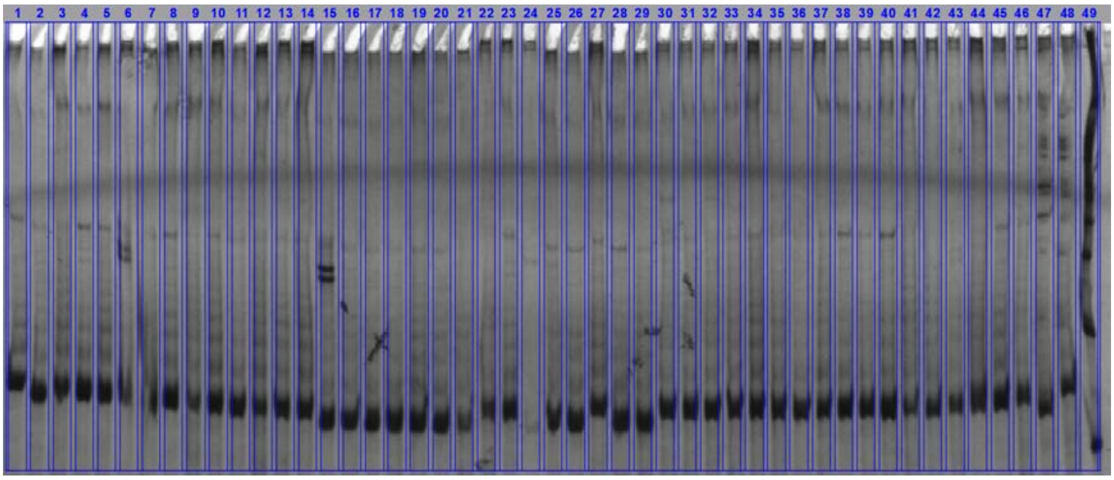
1 2 3 4 5 6 7 8 9 10 11 12 13 14 15 16 17 18 19 20 21 22 23 24 25 26 27 28 29 30 31 32 33 34 35 36 37 38 39 40 41 42 43 44 45 46 47 48 49

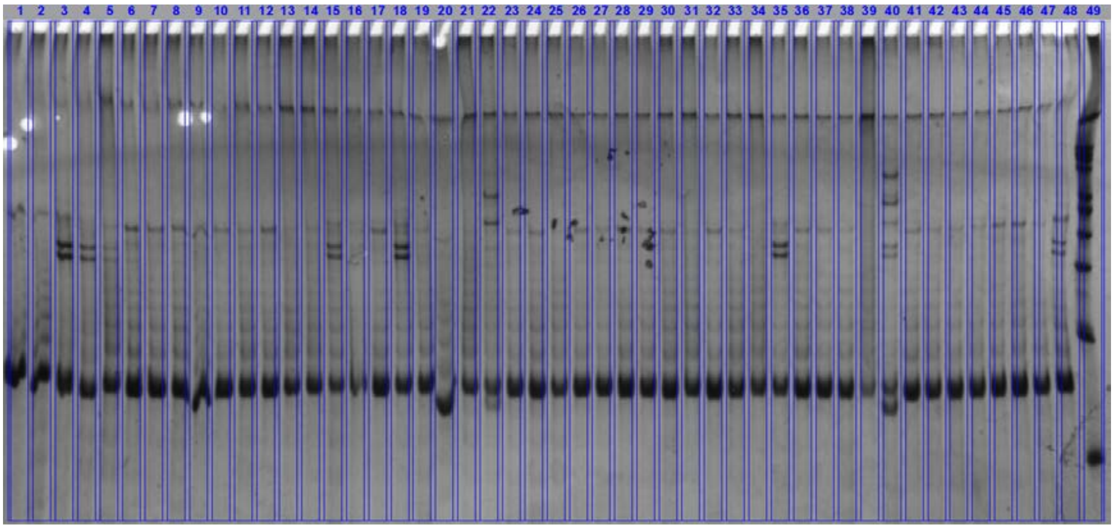
1 2 3 4 5 6 7 8 9 10 11 12 13 14 15 16 17 18 19 20 21 22 23 24 25 26 27 28 29 30 31 32 33 34 35 36 37 38 39 40 41 42 43 44 45 46 47 48 49

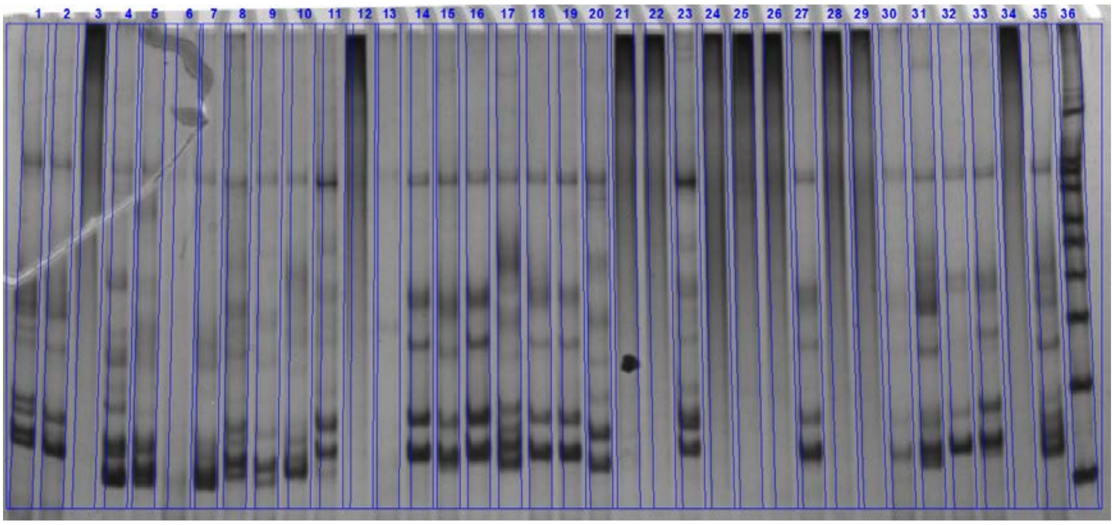
1 2 3 4 5 6 7 8 9 10 11 12 13 14 15 16 17 18 19 20 21 22 23 24 25 26 27 28 29 30 31 32 33 34 35 36

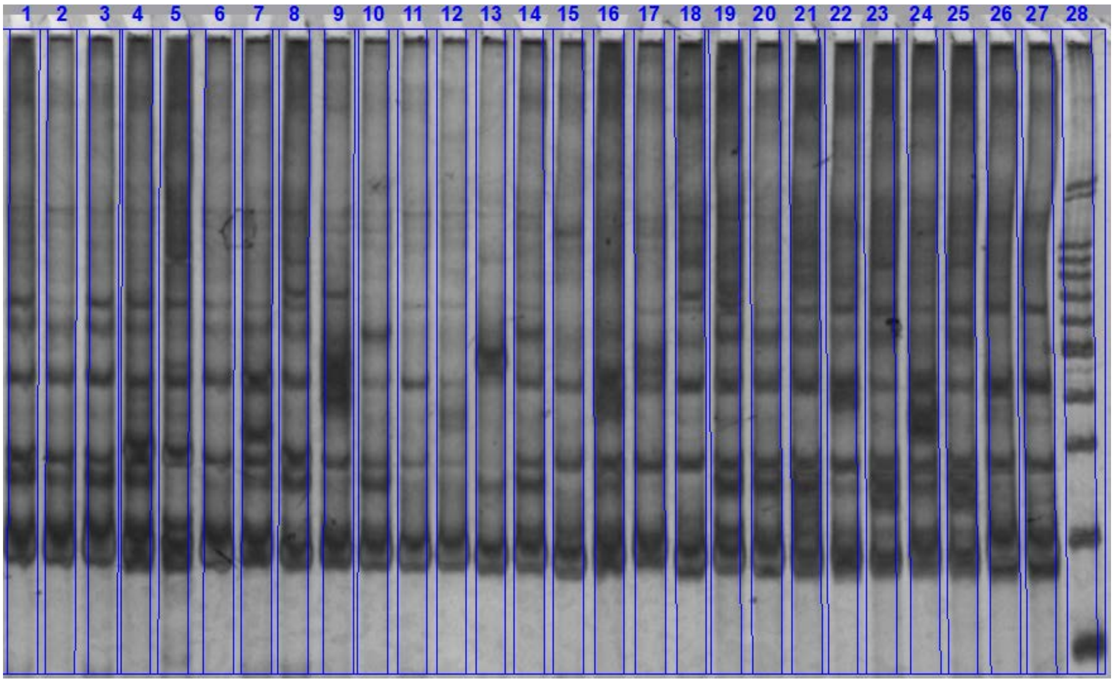

The gel images of primer X58H. The accessions from left to right in each gel image were 1, 2, 3, 4, 5, 6, 7, 8, 9, 10, 11, 13, 14, 16, 18, 19, 20, 21, 22, 23, 24, 25, 26, 27, 28, 29, 30, 31, 32, 33, 34, 35, 36, 37, 38, 39, 40, 41, 42, 43, 44, 45, 46, 47, 48, 49, 53, 55, 62, 71, 72, 81, 87, 89, 94, 99, 322, 324, 328, 329, 332, 334, 339, 341, 345, 350, 354, 358, 366,367, 368, 375, 381, 401, 404, 405, 406, 407, 408, 409, 421, 442, 443, 449, 455, 457, 459, 460, 462, 463, 464, 501, 502, 503, 504, 506, 507, 508, 509, 510, 511, 513, 516, 517, 518, 544, 558, 591, 592, 593, 594, 595, 596, 621, 622, 624, 626, 628, 754, 759, 771, 1001, 1002, 1003, 1004, 1005, 1006, BF, BX, BY01, BY02, BY03, JTY, KT, HW01, HW04, HW10, HW10, HW14, HW18, HW19, HW20, HL01, HL06, HL07, HL09, HL12, HL13, HL14, HZ03, HZ07, HZ09, HZ10, HZ11, HZ13, HZ14, XB.

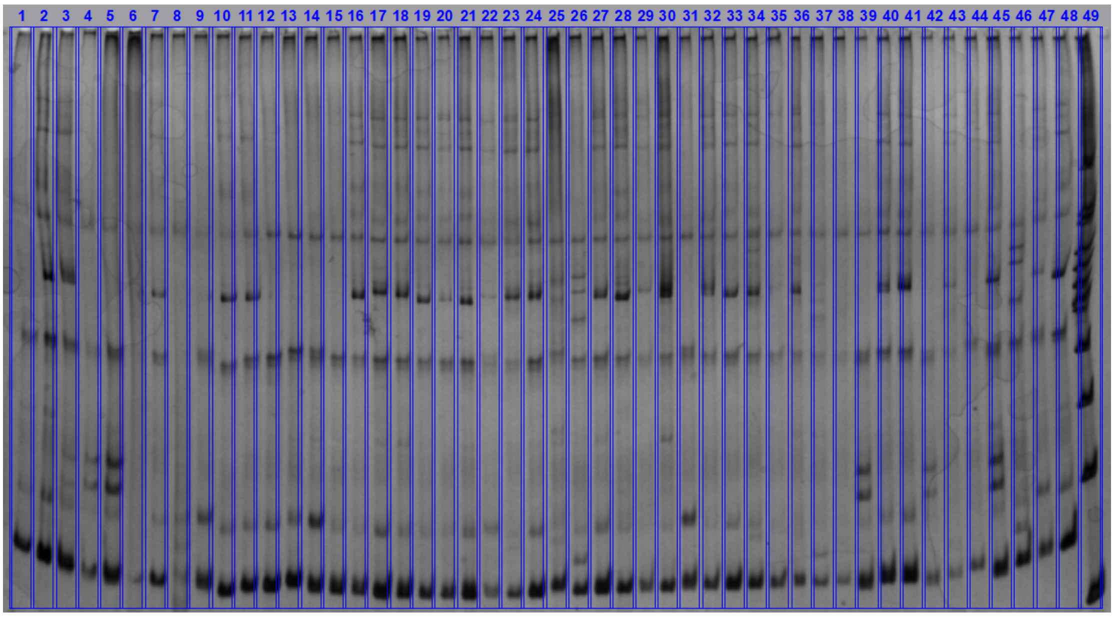
1 2 3 4 5 6 7 8 9 10 11 12 13 14 15 16 17 18 19 20 21 22 23 24 25 26 27 28 29 30 31 32 33 34 35 36 37 38 39 40 41 42 43 44 45 46 47 48 49

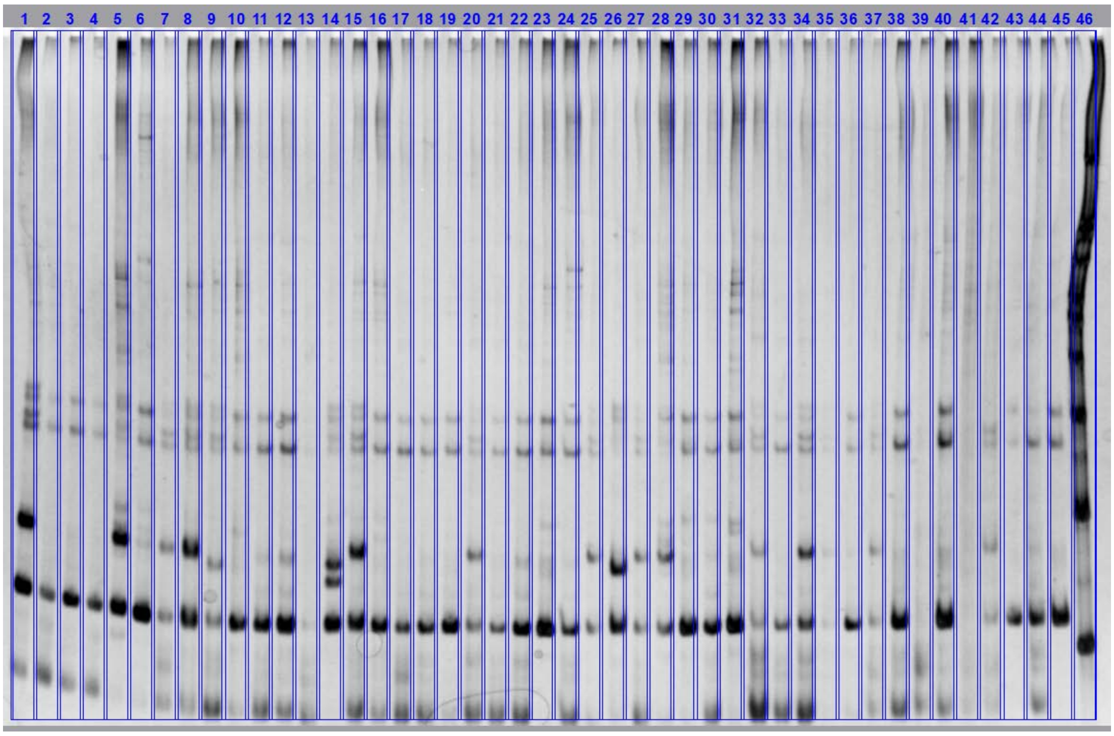
1 2 3 4 5 6 7 8 9 10 11 12 13 14 15 16 17 18 19 20 21 22 23 24 25 26 27 28 29 30 31 32 33 34 35 36 37 38 39 40 41 42 43 44 45 46

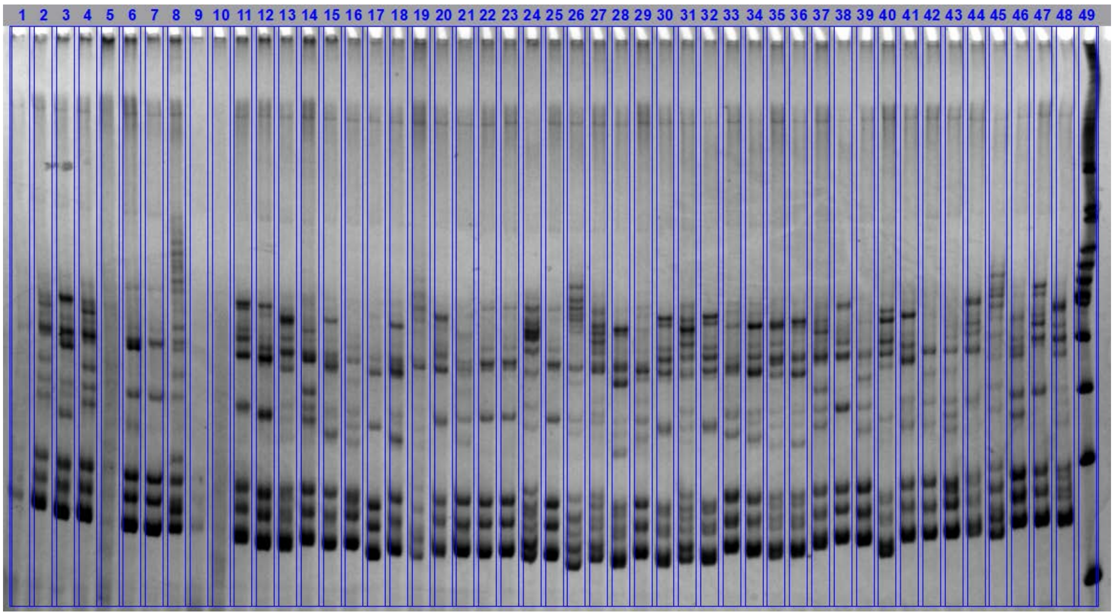

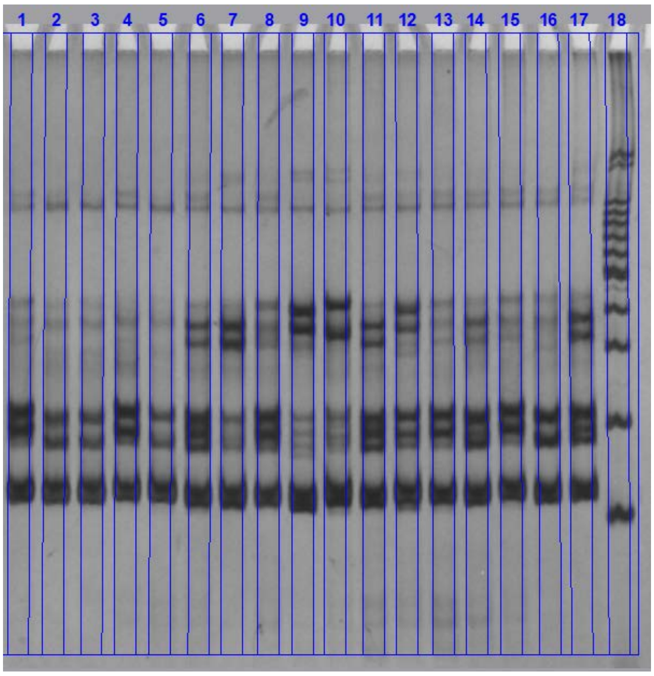

The gel images of primer X70. The accessions from left to right in each gel image were 1, 2, 3, 4, 5, 6, 7, 8, 9, 10, 11, 13, 14, 16, 18, 19, 20, 21, 22, 23, 24, 25, 26, 27, 28, 29, 30, 31, 32, 33, 34, 35, 36, 37, 38, 39, 40, 41, 42, 43, 44, 45, 46, 47, 48, 49, 53, 55, 62, 71, 72, 81, 87, 89, 94, 99, 322, 324, 328, 329, 332, 334, 339, 341, 345, 350, 354, 358, 366,367, 368, 375, 381, 401, 404, 405, 406, 407, 408, 409, 421, 442, 443, 449, 455, 457, 459, 460, 462, 463, 464, 501, 502, 503, 504, 506, 507, 508, 509, 510, 511, 513, 516, 517, 518, 544, 558, 591, 592, 593, 594, 595, 596, 621, 622, 624, 626, 628, 754, 759, 771, 1001, 1002, 1003, 1004, 1005, 1006, BF, BX, BY01, BY02, BY03, JTY, KT, HW01, HW04, HW10, HW10, HW14, HW18, HW19, HW20, HL01, HL06, HL07, HL09, HL12, HL13, HL14, HZ03, HZ07, HZ09, HZ10, HZ11, HZ13, HZ14, XB.

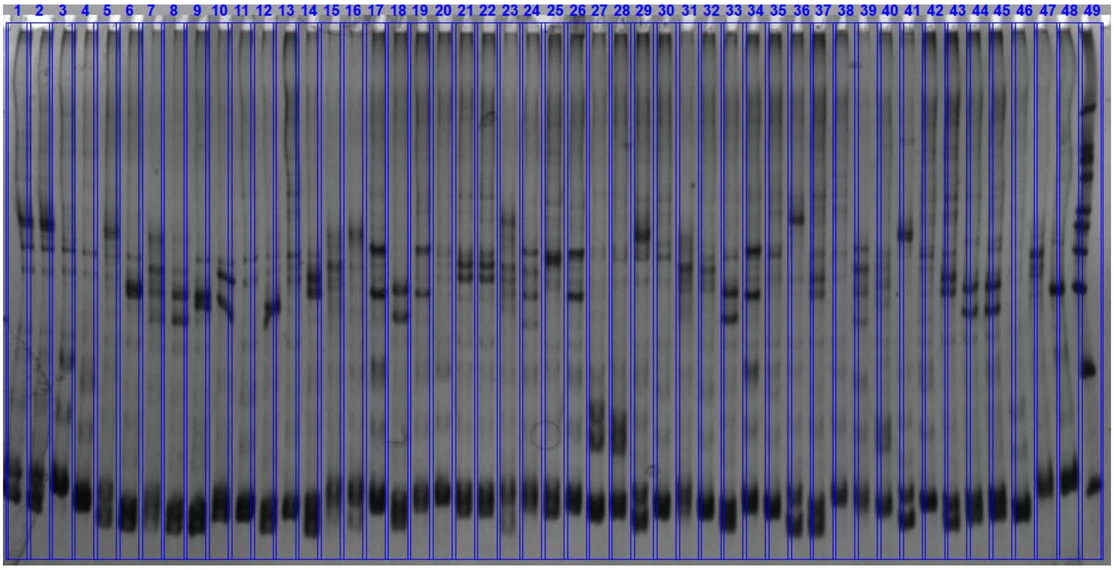
1 2 3 4 5 6 7 8 9 10 11 12 13 14 15 16 17 18 19 20 21 22 23 24 25 26 27 28 29 30 31 32 33 34 35 36 37 38 39 40 41 42 43 44 45 46 47 48 49
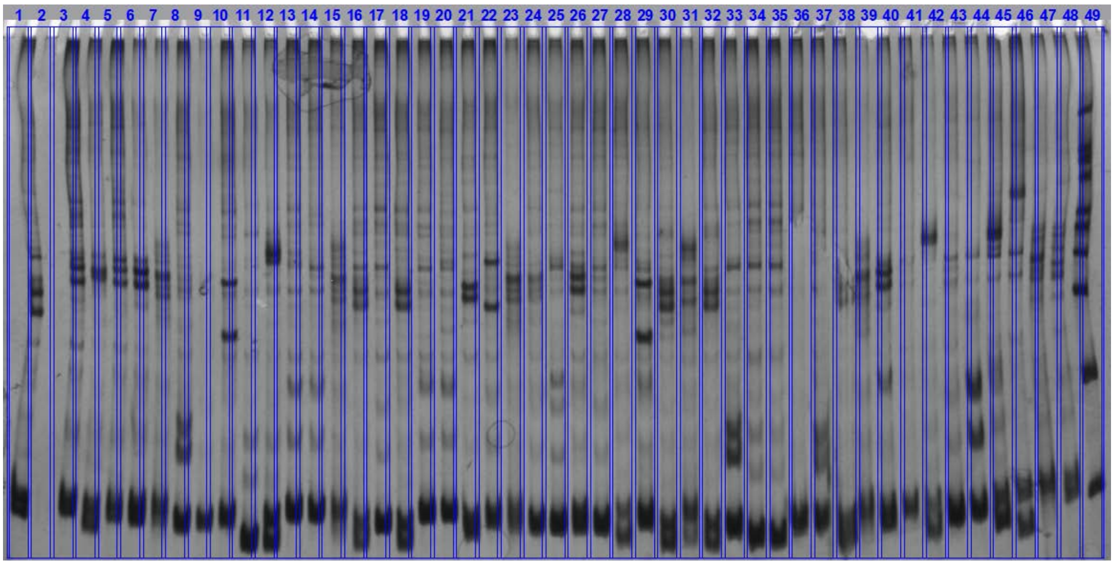
1 2 3 4 5 6 7 8 9 10 11 12 13 14 15 16 17 18 19 20 21 22 23 24 25 26 27 28 29 30 31 32 33 34 35 36 37 38 39 40 41 42 43 44 45 46 47 48 49
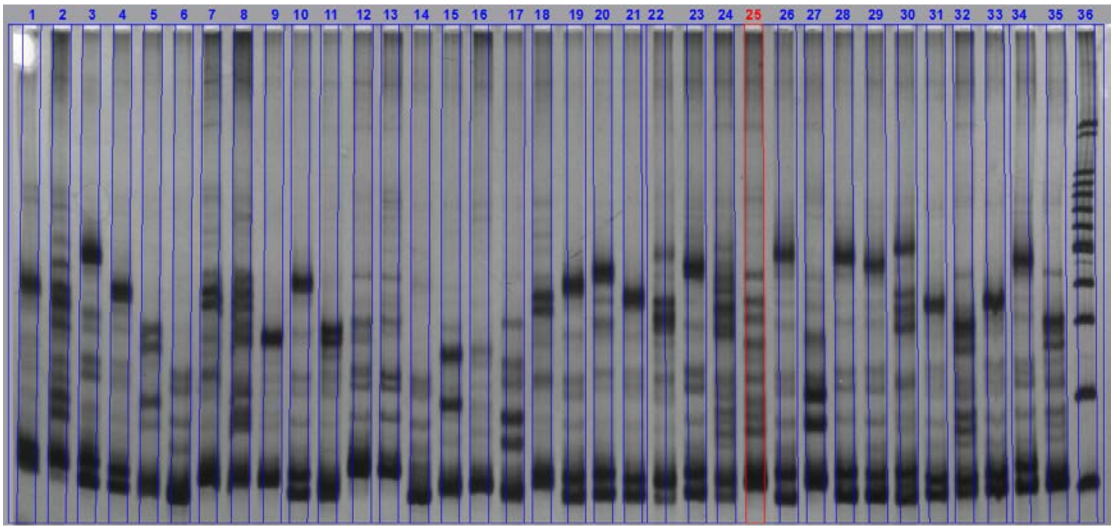
1 2 3 4 5 6 7 8 9 10 11 12 13 14 15 16 17 18 19 20 21 22 23 24 25 26 27 28 29 30 31 32 33 34 35 36

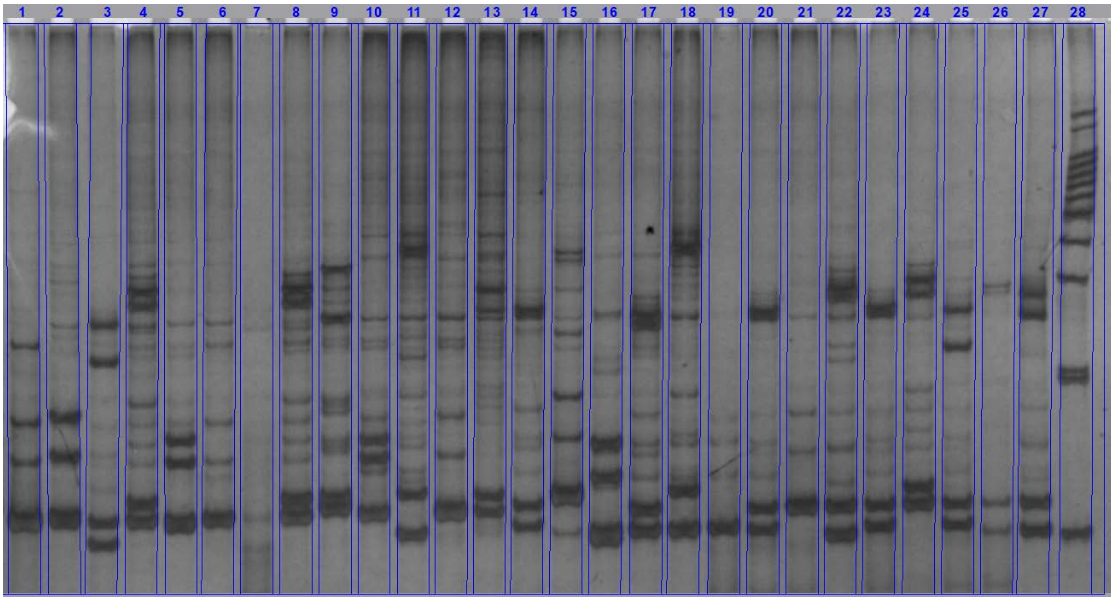

The gel images of primer X87. The accessions from left to right in each gel image were 1, 2, 3, 4, 5, 6, 7, 8, 9, 10, 11, 13, 14, 16, 18, 19, 20, 21, 22, 23, 24, 25, 26, 27, 28, 29, 30, 31, 32, 33, 34, 35, 36, 37, 38, 39, 40, 41, 42, 43, 44, 45, 46, 47, 48, 49, 53, 55, 62, 71, 72, 81, 87, 89, 94, 99, 322, 324, 328, 329, 332, 334, 339, 341, 345, 350, 354, 358, 366,367, 368, 375, 381, 401, 404, 405, 406, 407, 408, 409, 421, 442, 443, 449, 455, 457, 459, 460, 462, 463, 464, 501, 502, 503, 504, 506, 507, 508, 509, 510, 511, 513, 516, 517, 518, 544, 558, 591, 592, 593, 594, 595, 596, 621, 622, 624, 626, 628, 754, 759, 771, 1001, 1002, 1003, 1004, 1005, 1006, BF, BX, BY01, BY02, BY03, JTY, KT, HW01, HW04, HW10, HW10, HW14, HW18, HW19, HW20, HL01, HL06, HL07, HL09, HL12, HL13, HL14, HZ03, HZ07, HZ09, HZ10, HZ11, HZ13, HZ14, XB

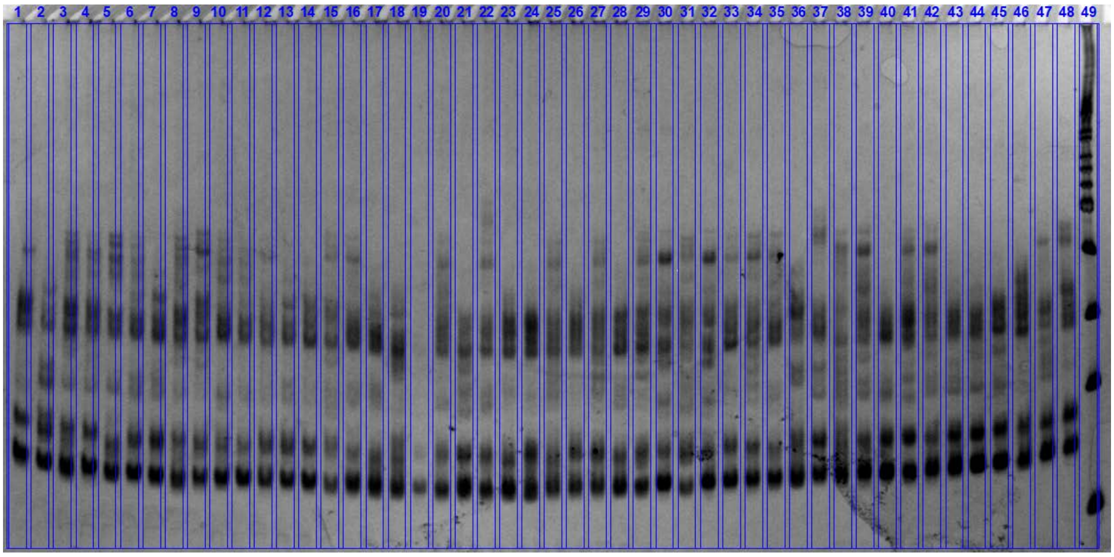
1 2 3 4 5 6 7 8 9 10 11 12 13 14 15 16 17 18 19 20 21 22 23 24 25 26 27 28 29 30 31 32 33 34 35 36 37 38 39 40 41 42 43 44 45 46 47 48 49

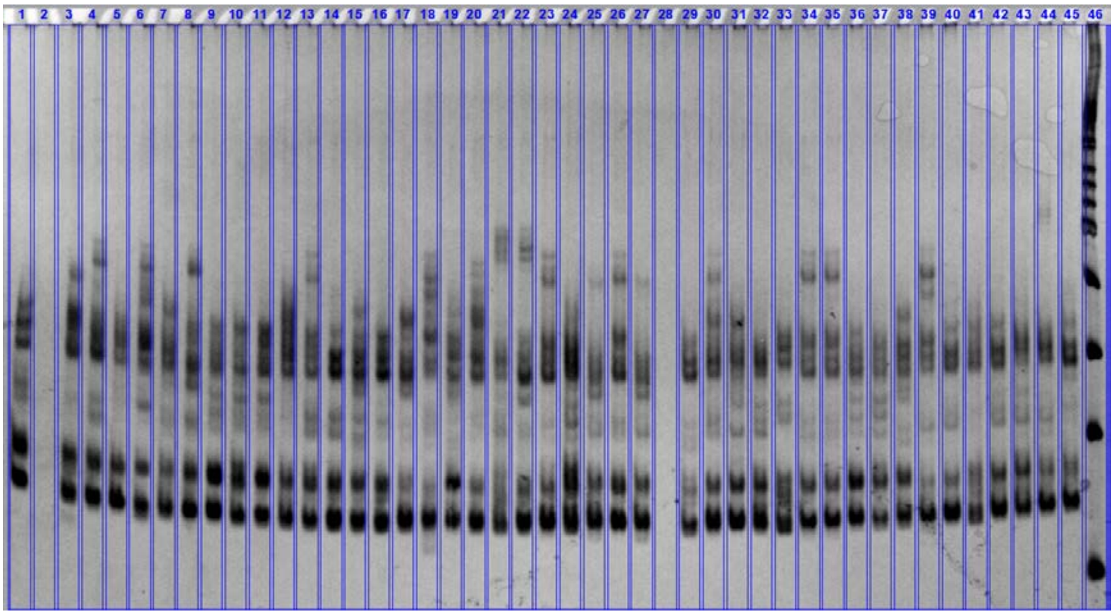
1 2 3 4 5 6 7 8 9 10 11 12 13 14 15 16 17 18 19 20 21 22 23 24 25 26 27 28 29 30 31 32 33 34 35 36 37 38 39 40 41 42 43 44 45 46

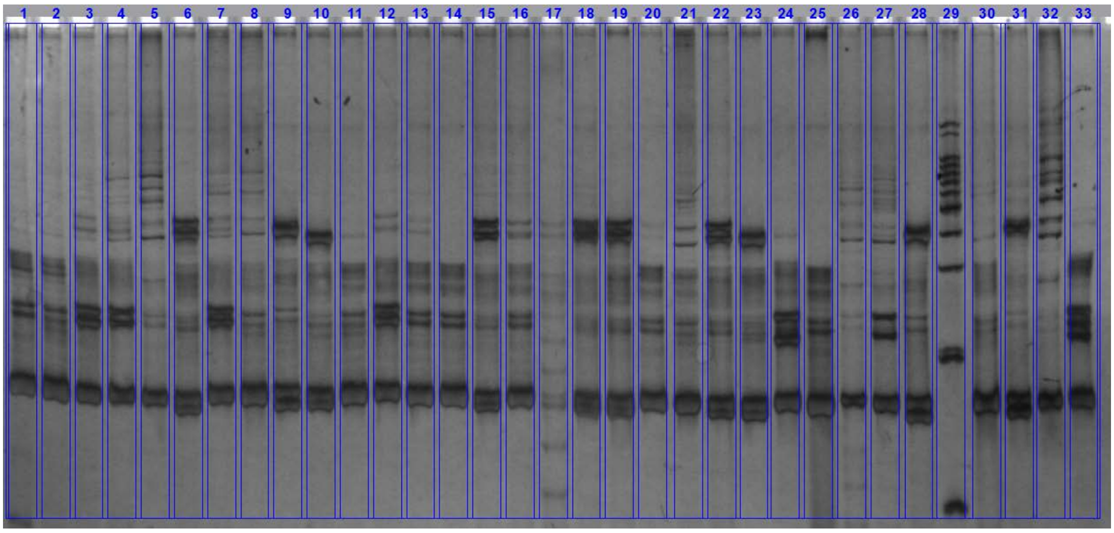
1 2 3 4 5 6 7 8 9 10 11 12 13 14 15 16 17 18 19 20 21 22 23 24 25 26 27 28 29 30 31 32 33

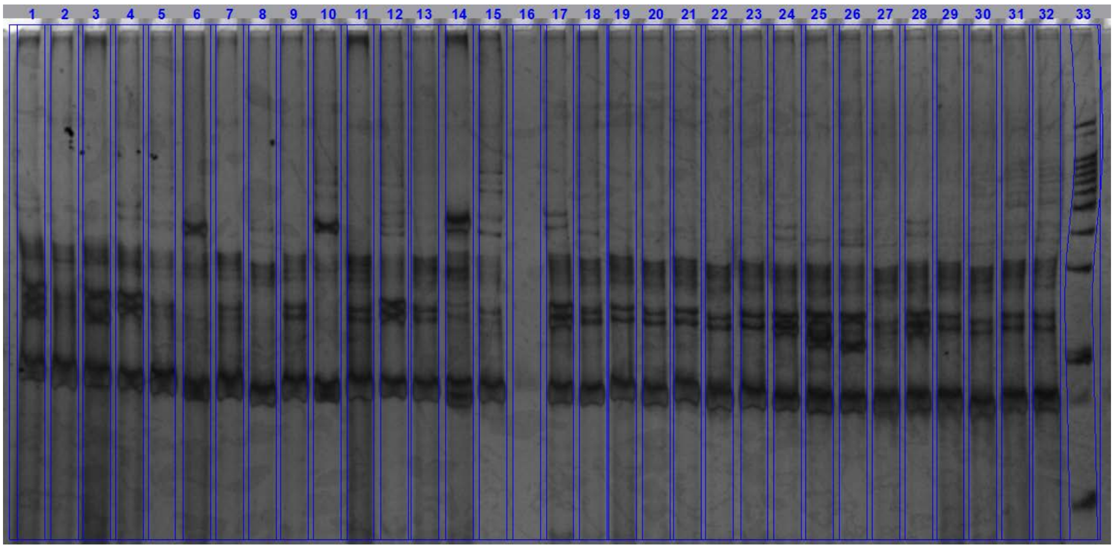

The gel images of primer Y5. The accessions from left to right in each gel image were 1, 2, 3, 4, 5, 6, 7, 8, 9, 10, 11, 13, 14, 16, 18, 19, 20, 21, 22, 23, 24, 25, 26, 27, 28, 29, 30, 31, 32, 33, 34, 35, 36, 37, 38, 39, 40, 41, 42, 43, 44, 45, 46, 47, 48, 49, 53, 55, 62, 71, 72, 81, 87, 89, 94, 99, 322, 324, 328, 329, 332, 334, 339, 341, 345, 350, 354, 358, 366,367, 368, 375, 381, 401, 404, 405, 406, 407, 408, 409, 421, 442, 443, 449, 455, 457, 459, 460, 462, 463, 464, 501, 502, 503, 504, 506, 507, 508, 509, 510, 511, 513, 516, 517, 518, 544, 558, 591, 592, 593, 594, 595, 596, 621, 622, 624, 626, 628, 754, 759, 771, 1001, 1002, 1003, 1004, 1005, 1006, BF, BX, BY01, BY02, BY03, JTY, KT, HW01, HW04, HW10, HW10, HW14, HW18, HW19, HW20, HL01, HL06, HL07, HL09, HL12, HL13, HL14, HZ03, HZ07, HZ09, HZ10, HZ11, HZ13, HZ14, XB.

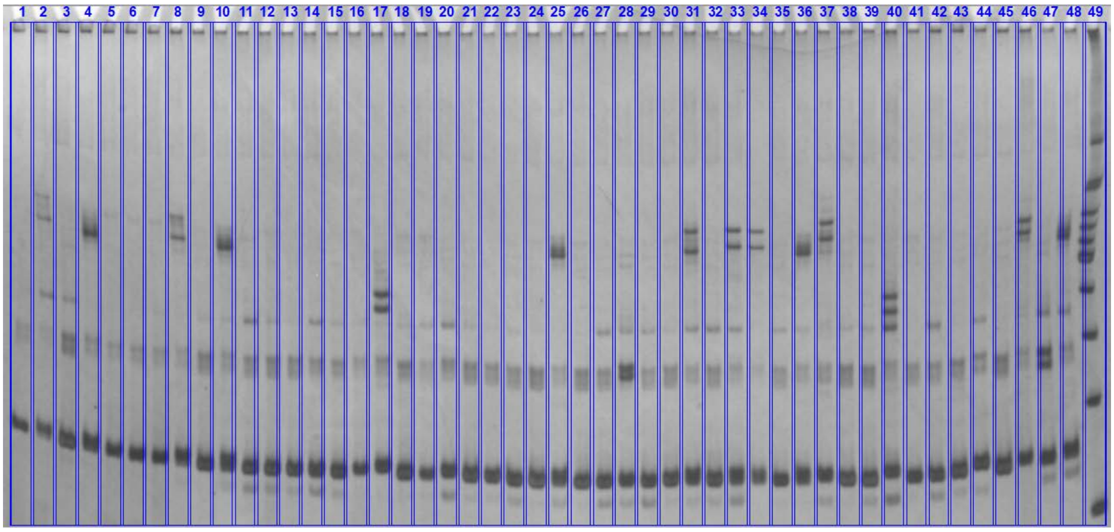
1 2 3 4 5 6 7 8 9 10 11 12 13 14 15 16 17 18 19 20 21 22 23 24 25 26 27 28 29 30 31 32 33 34 35 36 37 38 39 40 41 42 43 44 45 46 47 48 49

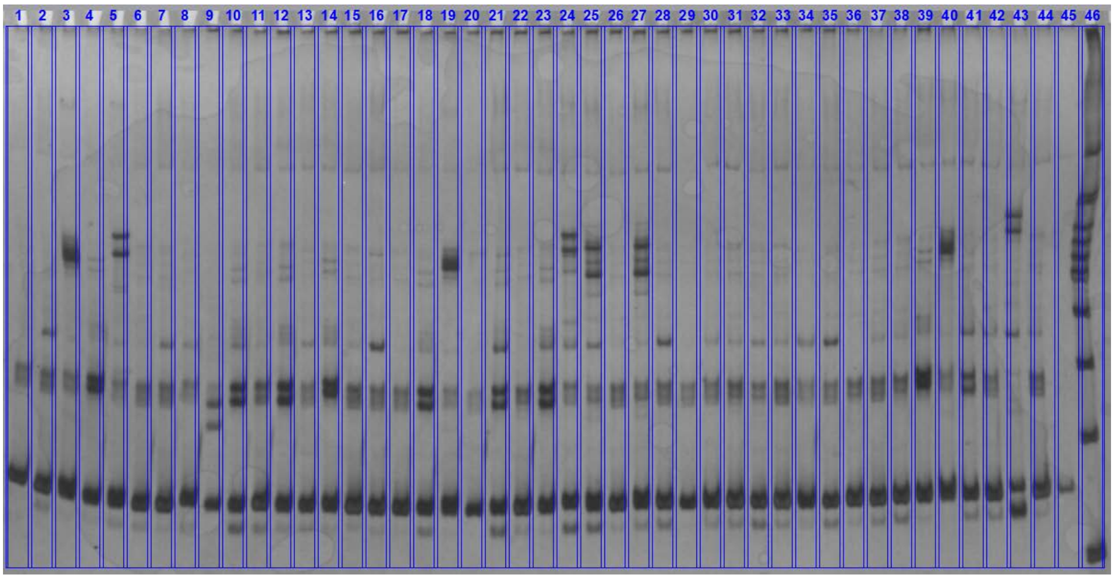
1 2 3 4 5 6 7 8 9 10 11 12 13 14 15 16 17 18 19 20 21 22 23 24 25 26 27 28 29 30 31 32 33 34 35 36 37 38 39 40 41 42 43 44 45 46

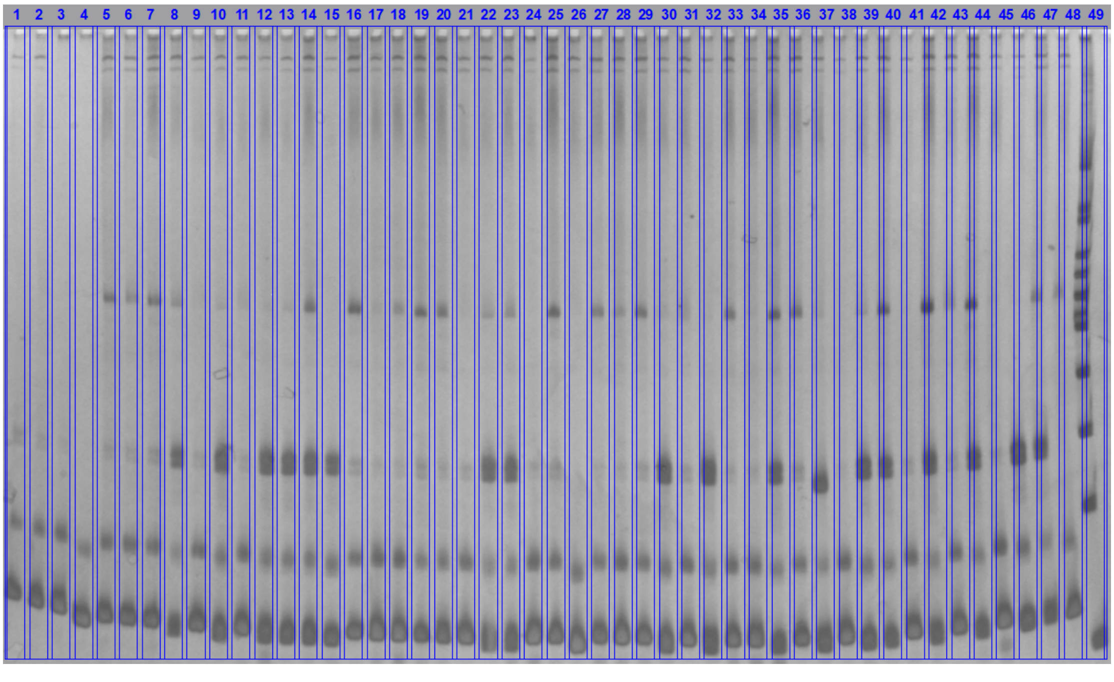
1 2 3 4 5 6 7 8 9 10 11 12 13 14 15 16 17 18 19 20 21 22 23 24 25 26 27 28 29 30 31 32 33 34 35 36 37 38 39 40 41 42 43 44 45 46 47 48 49

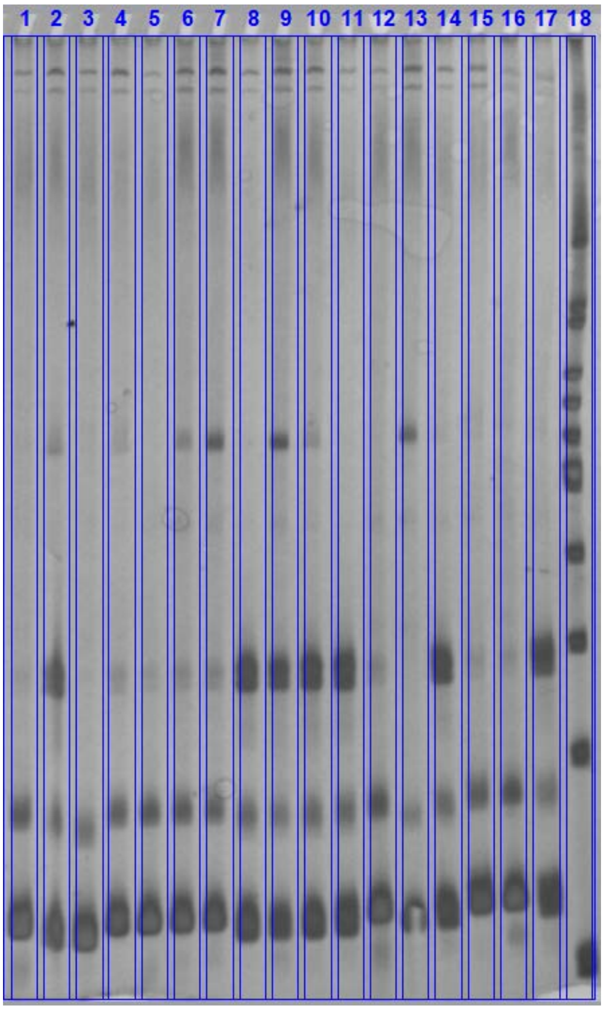

The gel images of primer Y48. The accessions from left to right in each gel image were 1, 2, 3, 4, 5, 6, 7, 8, 9, 10, 11, 13, 14, 16, 18, 19, 20, 21, 22, 23, 24, 25, 26, 27, 28, 29, 30, 31, 32, 33, 34, 35, 36, 37, 38, 39, 40, 41, 42, 43, 44, 45, 46, 47, 48, 49, 53, 55, 62, 71, 72, 81, 87, 89, 94, 99, 322, 324, 328, 329, 332, 334, 339, 341, 345, 350, 354, 358, 366,367, 368, 375, 381, 401, 404, 405, 406, 407, 408, 409, 421, 442, 443, 449, 455, 457, 459, 460, 462, 463, 464, 501, 502, 503, 504, 506, 507, 508, 509, 510, 511, 513, 516, 517, 518, 544, 558, 591, 592, 593, 594, 595, 596, 621, 622, 624, 626, 628, 754, 759, 771, 1001, 1002, 1003, 1004, 1005, 1006, BF, BX, BY01, BY02, BY03, JTY, KT, HW01, HW04, HW10, HW10, HW14, HW18, HW19, HW20, HL01, HL06, HL07, HL09, HL12, HL13, HL14, HZ03, HZ07, HZ09, HZ10, HZ11, HZ13, HZ14, XB.

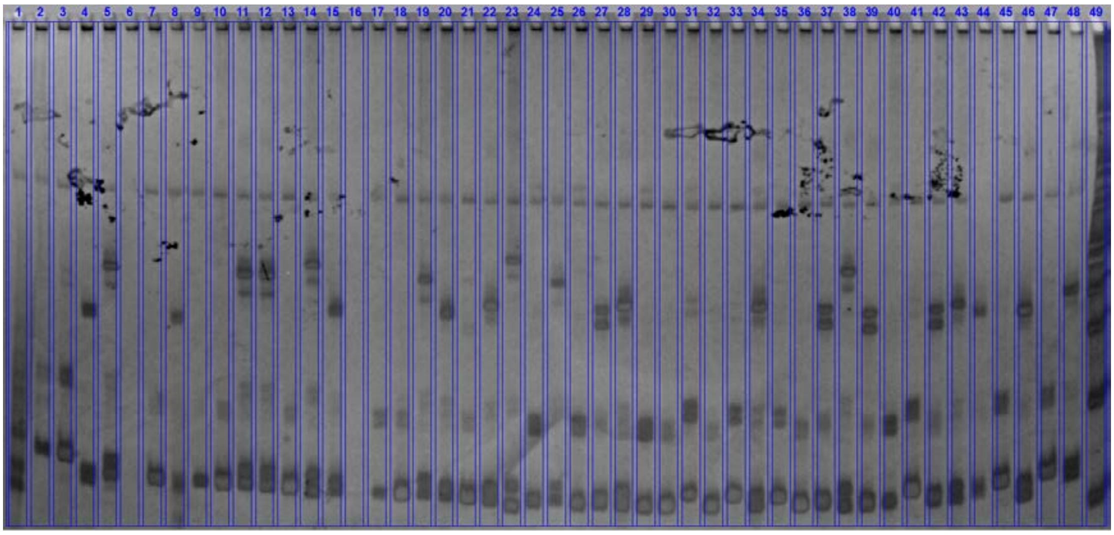
1 2 3 4 5 6 7 8 9 10 11 12 13 14 15 16 17 18 19 20 21 22 23 24 25 26 27 28 29 30 31 32 33 34 35 36 37 38 39 40 41 42 43 44 45 46 47 48 49

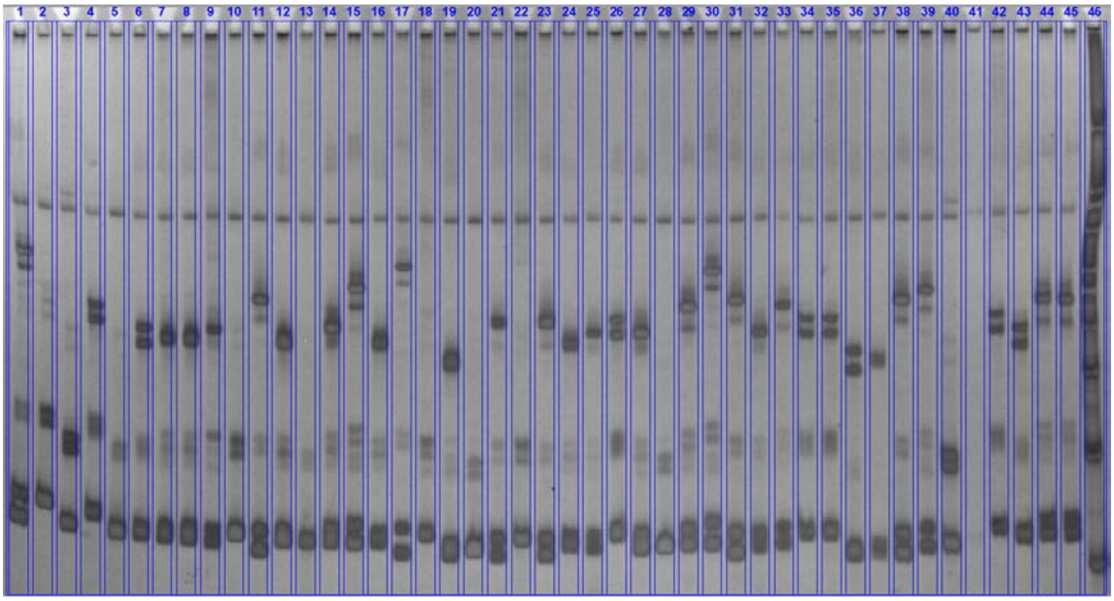
1 2 3 4 5 6 7 8 9 10 11 12 13 14 15 16 17 18 19 20 21 22 23 24 25 26 27 28 29 30 31 32 33 34 35 36 37 38 39 40 41 42 43 44 45 46

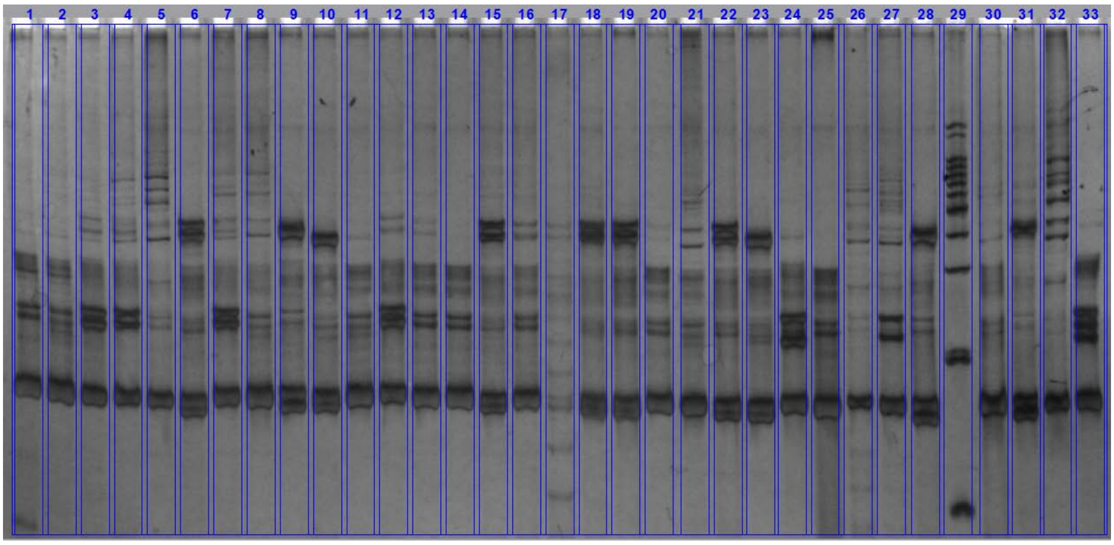
1 2 3 4 5 6 7 8 9 10 11 12 13 14 15 16 17 18 19 20 21 22 23 24 25 26 27 28 29 30 31 32 33

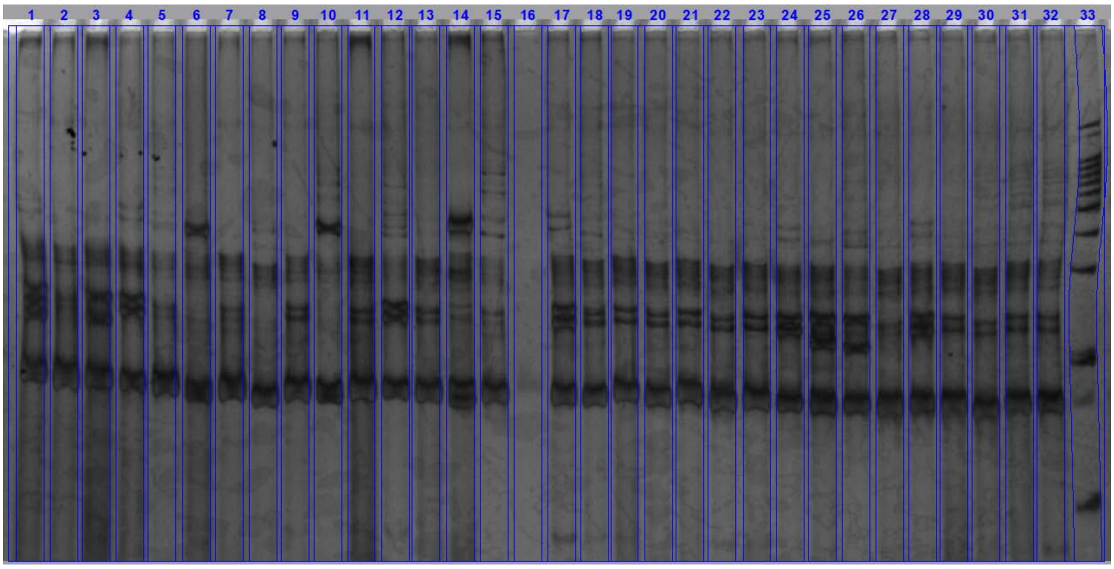

The gel images of primer Y50. The accessions from left to right in each gel image were 1, 2, 3, 4, 5, 6, 7, 8, 9, 10, 11, 13, 14, 16, 18, 19, 20, 21, 22, 23, 24, 25, 26, 27, 28, 29, 30, 31, 32, 33, 34, 35, 36, 37, 38, 39, 40, 41, 42, 43, 44, 45, 46, 47, 48, 49, 53, 55, 62, 71, 72, 81, 87, 89, 94, 99, 322, 324, 328, 329, 332, 334, 339, 341, 345, 350, 354, 358, 366,367, 368, 375, 381, 401, 404, 405, 406, 407, 408, 409, 421, 442, 443, 449, 455, 457, 459, 460, 462, 463, 464, 501, 502, 503, 504, 506, 507, 508, 509, 510, 511, 513, 516, 517, 518, 544, 558, 591, 592, 593, 594, 595, 596, 621, 622, 624, 626, 628, 754, 759, 771, 1001, 1002, 1003, 1004, 1005, 1006, BF, BX, BY01, BY02, BY03, JTY, KT, HW01, HW04, HW10, HW10, HW14, HW18, HW19, HW20, HL01, HL06, HL07, HL09, HL12, HL13, HL14, HZ03, HZ07, HZ09, HZ10, HZ11, HZ13, HZ14, XB.

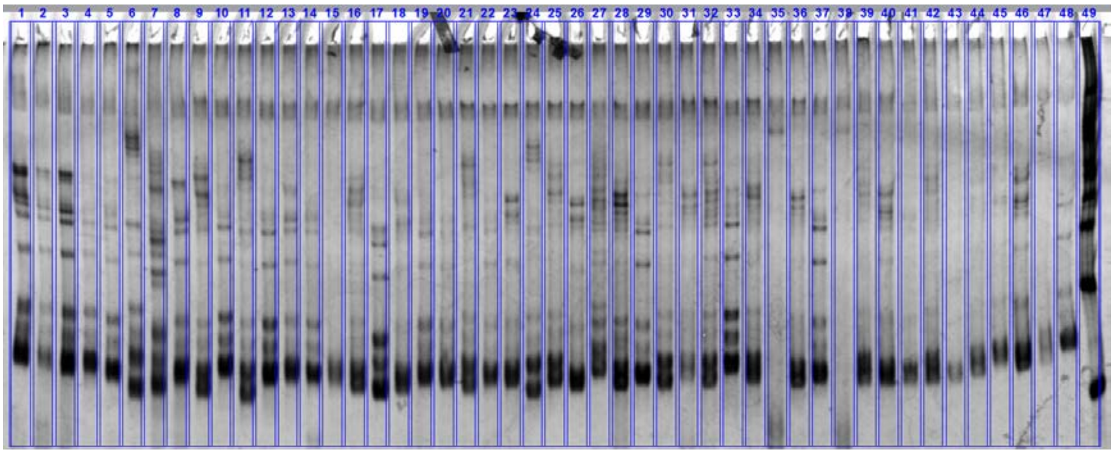
1 2 3 4 5 6 7 8 9 10 11 12 13 14 15 16 17 18 19 20 21 22 23 24 25 26 27 28 29 30 31 32 33 34 35 36 37 38 39 40 41 42 43 44 45 46 47 48 49

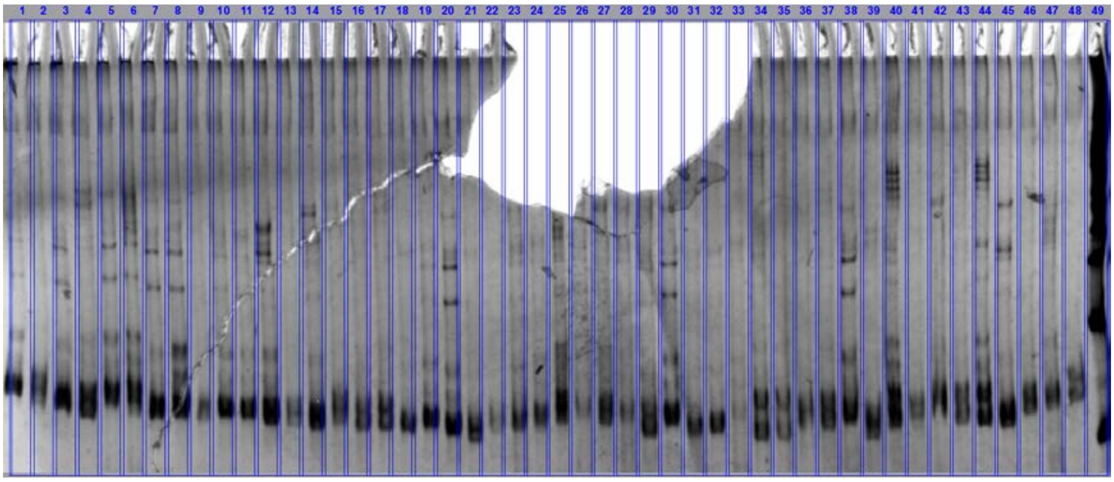
1 2 3 4 5 6 7 8 9 10 11 12 13 14 15 16 17 18 19 20 21 22 23 24 25 26 27 28 29 30 31 32 33 34 35 36 37 38 39 40 41 42 43 44 45 46 47 48 49

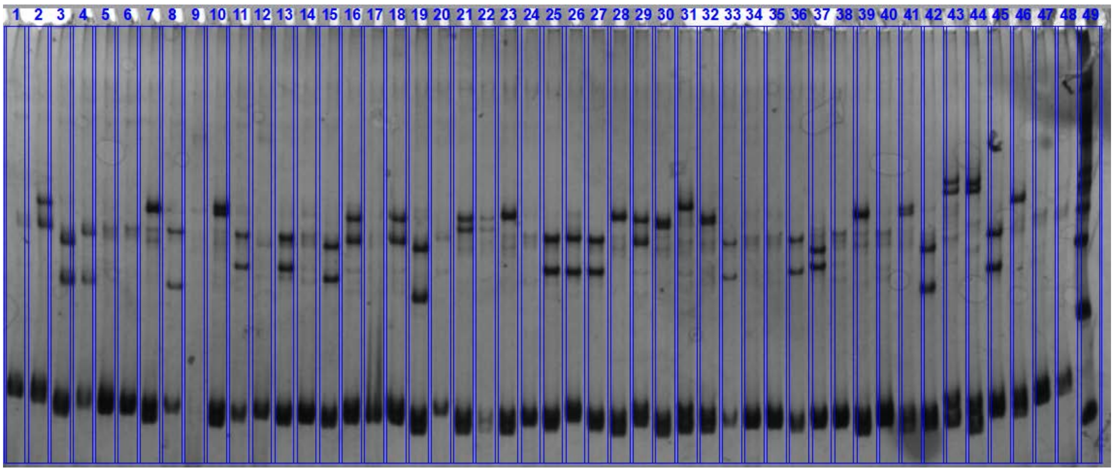
1 2 3 4 5 6 7 8 9 10 11 12 13 14 15 16 17 18 19 20 21 22 23 24 25 26 27 28 29 30 31 32 33 34 35 36 37 38 39 40 41 42 43 44 45 46 47 48 49

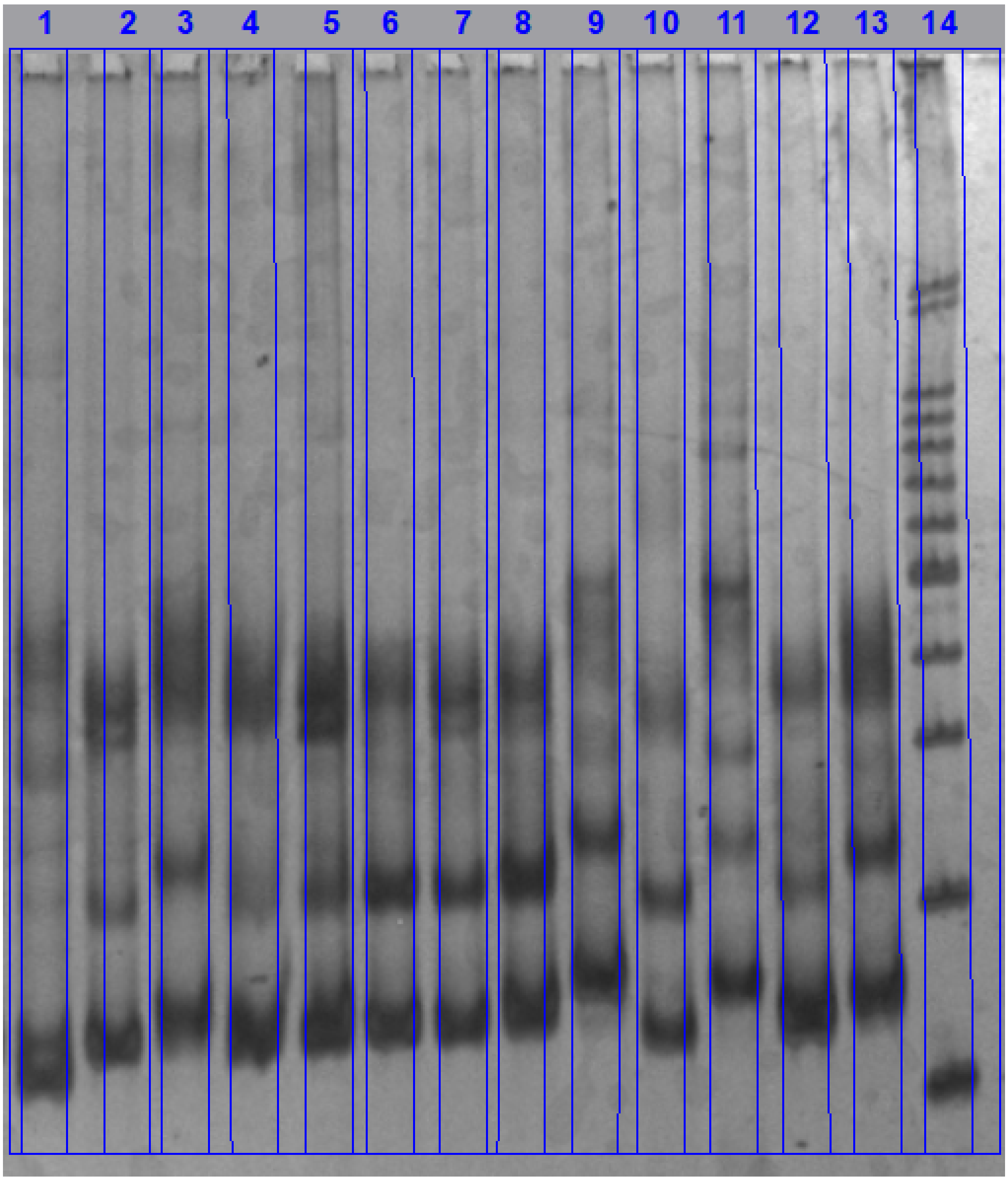

The gel images of primer Y65. The accessions from left to right in each gel image were 1, 2, 3, 4, 5, 6, 7, 8, 9, 10, 11, 13, 14, 16, 18, 19, 20, 21, 22, 23, 24, 25, 26, 27, 28, 29, 30, 31, 32, 33, 34, 35, 36, 37, 38, 39, 40, 41, 42, 43, 44, 45, 46, 47, 48, 49, 53, 55, 62, 71, 72, 81, 87, 89, 94, 99, 322, 324, 328, 329, 332, 334, 339, 341, 345, 350, 354, 358, 366,367, 368, 375, 381, 401, 404, 405, 406, 407, 408, 409, 421, 442, 443, 449, 455, 457, 459, 460, 462, 463, 464, 501, 502, 503, 504, 506, 507, 508, 509, 510, 511, 513, 516, 517, 518, 544, 558, 591, 592, 593, 594, 595, 596, 621, 622, 624, 626, 628, 754, 759, 771, 1001, 1002, 1003, 1004, 1005, 1006, BF, BX, BY01, BY02, BY03, JTY, KT, HW01, HW04, HW10, HW10, HW14, HW18, HW19, HW20, HL01, HL06, HL07, HL09, HL12, HL13, HL14, HZ03, HZ07, HZ09, HZ10, HZ11, HZ13, HZ14, XB.
