# Supplementary material for: Selection of a core collection of Prunus sibirica L. germplasm by a stepwise clustering method using simple sequence repeat markers
Source: PLoS One. 2021 Nov 19;16(11):e0260097. doi: 10.1371/journal.pone.0260097 (PMC8604298; doi:10.1371/journal.pone.0260097)
Supplement: S1 Table — (DOCX) [file pone.0260097.s002.docx]

**S1 Table. The 158 *Prunus sibirica* accessions for test material**

| **Accessions** | **Provenance** | **Germplasm** **characteristics** | **Accessions** | **Provenance** | **Germplasm characteristics** |
| --- | --- | --- | --- | --- | --- |
| 1 | Kazuo  Liaoning | High yield  Bitter almond | 408 | Zhalantun  Inner Mongolia | Late-flowering  Bitter almond |
| 2* | Kazuo  Liaoning | High yield  Bitter almond | 409 | Zhalantun  Inner Mongolia | Late-flowering  Bitter almond |
| 3 | Kazuo  Liaoning | Double kernels  Bitter almond | 421* | Zhalantun  Inner Mongolia | High yield  Frost resistance  Bitter almond |
| 4 | Kazuo  Liaoning | Double kernels  Sweet almond | 442 | Zhalantun  Inner Mongolia | Bent branch  Bitter almond |
| 5 | Kazuo  Liaoning | Double kernels  Bitter almond | 443 | Zhalantun  Inner Mongolia | Double pistil  Bitter almond |
| 6 | Kazuo  Liaoning | Frost resistance  Bitter almond | 449 | Zhalantun  Inner Mongolia | Big flower  Bitter almond |
| 7* | Kazuo  Liaoning | High yield  Bitter almond | 455 | Zhalantun  Inner Mongolia | Pink anther  Bitter almond |
| 8 | Kazuo  Liaoning | High yield  Bitter almond | 457 | Zhalantun  Inner Mongolia | Pink flower  Bitter almond |
| 9 | Kazuo  Liaoning | High yield  Bitter almond | 459 | Zhalantun  Inner Mongolia | Red fruit  Bitter almond |
| 10 | Kazuo  Liaoning | High yield  Bitter almond | 460 | Zhalantun  Inner Mongolia | Long fruit  Bitter almond |
| 11 | Kazuo  Liaoning | High yield  Bitter almond | 462 | Zhalantun  Inner Mongolia | Sweet flesh  Bitter almond |
| 13 | Kazuo  Liaoning | High yield  Bitter almond | 463* | Zhalantun  Inner Mongolia | Sweet flesh  Bitter almond |
| 14 | Kazuo  Liaoning | Late-flowering  Double kernels | 464 | Zhalantun  Inner Mongolia | Sweet flesh  Bitter almond |
| 16* | Kazuo  Liaoning | Late-flowering  Double kernels  Sweet flesh  High yield | 501 | Russia | Extreme drought resistance  Bitter almond |
| 18 | Kazuo  Liaoning | High yield  Bitter almond | 502 | Russia | Extreme drought resistance  Bitter almond |
| 19 | Kazuo  Liaoning | High yield  Bitter almond | 503* | Russia | Extreme drought resistance |
| 20* | Kazuo  Liaoning | High yield  Bitter almond | 504 | Russia | Extreme drought resistance  Bitter almond |
| 21 | Kazuo  Liaoning | High yield  Bitter almond | 506 | Russia | Extreme drought resistance  Bitter almond |
| 22 | Kazuo  Liaoning | High yield  Bitter almond | 507 | Russia | Extreme drought resistance  Bitter almond |
| 23 | Kazuo  Liaoning | High yield  Bitter almond | 508 | Russia | Extreme drought resistance  Bitter almond |
| 24 | Aohan  Inner Mongolia | High yield  Bitter almond | 509 | Russia | Extreme drought resistance  Bitter almond |
| 25* | Aohan  Inner Mongolia | High yield  Bitter almond | 510 | Russia | Extreme drought resistance  Bitter almond |
| 26 | Aohan  Inner Mongolia | High yield  Bitter almond | 511^#^ | Russia | Drought resistance Bitter almond  High yield |
| 27 | Aohan  Inner Mongolia | High yield  Bitter almond | 513 | Russia | Drought resistance Bitter almond  High yield |
| 28 | Aohan  Inner Mongolia | High yield  Bitter almond | 516 | Russia | Drought resistance  Bitter almond  High yield |
| 29* | Chaoyang Liaoning | High yield  Bitter almond | 517 | Russia | Drought resistance Bitter almond  High yield |
| 30* | Chaoyang Liaoning | High yield  Bitter almond | 518* | Russia | Dro Bitter almond ught resistance  High yield |
| 31 | Aohan  Inner Mongolia | High yield  Bitter almond | 544* | Jilin | High yield  Bitter almond |
| 32 | Aohan  Inner Mongolia | High yield  Bitter almond | 558 | Jilin | High yield  Bitter almond |
| 33 | Aohan  Inner Mongolia | High yield  Bitter almond | 591* | Jilin | High yield  Bitter almond |
| 34 | Aohan  Inner Mongolia | High yield  Bitter almond | 592 | Jilin | High yield  Bitter almond |
| 35 | Aohan  Inner Mongolia | High yield  Bitter almond | 593* | Jilin | High yield  Bitter almond |
| 36 | Aohan  Inner Mongolia | High yield  Bitter almond | 594 | Jilin | High yield  Bitter almond |
| 37 | Aohan  Inner Mongolia | High yield  Bitter almond | 595 | Jilin | High yield  Bitter almond |
| 38 | Chaoyang Liaoning | High yield  Bitter almond | 596 | Jilin | High yield  Bitter almond |
| 39 | Aohan  Inner Mongolia | High yield  Late-flowering  Bitter almond | 621 | Shanxi | High yield  Bitter almond |
| 40* | Chaoyang Liaoning | High yield  Bitter almond | 622 | Shanxi | High yield  Bitter almond |
| 41 | Chaoyang Liaoning | Frost resistance  Bitter almond | 624 | Shanxi | High yield  Bitter almond |
| 42 | Chaoyang Liaoning | Sweet almond  Bitter almond | 626* | Shanxi | High yield  Bitter almond |
| 43 | Chaoyang  Liaoning | Sweet almond  Bitter almond | 628 | Shanxi | High yield  Bitter almond |
| 44* | Chaoyang  Liaoning | Sweet almond  Bitter almond | 754* | Jilin | High yield  Bitter almond |
| 45 | Chaoyang  Liaoning | Late-flowering  Bitter almond | 759* | Jilin | High yield  Bitter almond |
| 46 | Chaoyang  Liaoning | Late-flowering  Bitter almond | 771 | Heilongjiang | High yield  Bitter almond |
| 47 | Beipiao  Liaoning | High yield  Bitter almond | 1001* | Linkou  Heilongjiang | Bitter almond |
| 48 | Chaoyang  Liaoning | Late-maturing  Bitter almond | 1002 | Linkou  Heilongjiang | Bitter almond |
| 49 | Beipiao  Liaoning | High yield  Bitter almond | 1003* | Linkou  Heilongjiang | Bitter almond |
| 53 | Beipiao  Liaoning | High yield  Bitter almond | 1004 | Linkou  Heilongjiang | Bitter almond |
| 55 | Beipiao  Liaoning | High yield  Bitter almond | 1005 | Linkou  Heilongjiang | Bitter almond |
| 62* | Beipiao  Liaoning | High yield  Bitter almond | 1006 | Linkou  Heilongjiang | Bitter almond |
| 71* | Beipiao, Liaoning | High yield  Bitter almond | BF | Beipiao, Liaoning | High yield  Bitter almond |
| 72 | Beipiao, Liaoning | High yield  Bitter almond | BX | Beipiao, Liaoning | High yield  Bitter almond |
| 81* | Beipiao, Liaoning | High yield  Bitter almond | BY01 | Yanqing  Beijing | High yield  Bitter almond |
| 87* | Beipiao, Liaoning | High yield  Bitter almond | BY02* | Yanqing  Beijing | High yield  Bitter almond |
| 89* | Beipiao, Liaoning | High yield  Bitter almond | BY03 | Yanqing  Beijing | High yield  Bitter almond |
| 94* | Russia | Late-flowering  Bitter almond | JTY* | Kazuo  Liaoning | High yield  Bitter almond |
| 95 | Beipiao, Liaoning | Pink flower  Bitter almond | KT | Kazuo  Liaoning | High yield  Bitter almond |
| 99 | Chaoyang, Liaoning | Fold flower  Bitter almond | HW01* | Weichang  Hebei | High yield  Bitter almond |
| 322 | Zhalantun  Inner Mongolia | High yield  Bitter almond | HW04 | Weichang  Hebei | High yield  Bitter almond |
| 324 | Zhalantun  Inner Mongolia | High yield  Bitter almond | HW10* | Weichang  Hebei | High yield  Bitter almond |
| 328 | Zhalantun  Inner Mongolia | High yield  Bitter almond | HW13 | Weichang  Hebei | High yield  Bitter almond |
| 329 | Zhalantun  Inner Mongolia | High yield  Bitter almond | HW14 | Weichang  Hebei | High yield  Bitter almond |
| 332 | Zhalantun  Inner Mongolia | High yield  Bitter almond | HW18 | Weichang  Hebei | High yield  Bitter almond |
| 334 | Zhalantun  Inner Mongolia | High yield  Bitter almond | HW19 | Weichang  Hebei | High yield  Bitter almond |
| 339 | Zhalantun  Inner Mongolia | High yield  Bitter almond | HW20 | Weichang  Hebei | High yield  Bitter almond |
| 341* | Zhalantun  Inner Mongolia | High yield  Bitter almond | HL01 | Luanping  Hebei | High yield  Bitter almond |
| 345 | Zhalantun  Inner Mongolia | High yield  Bitter almond | HL06 | Luanping  Hebei | High yield  Bitter almond |
| 350 | Zhalantun  Inner Mongolia | High yield  Bitter almond | HL07* | Luanping  Hebei | High yield  Bitter almond |
| 354^#^ | Zhalantun  Inner Mongolia | High yield  Bitter almond | HL09 | Luanping  Hebei | High yield  Bitter almond |
| 358 | Zhalantun  Inner Mongolia | High yield  Bitter almond | HL12* | Luanping  Hebei | High yield  Bitter almond |
| 366^#^ | Zhalantun  Inner Mongolia | High yield  Bitter almond | HL13 | Luanping  Hebei | High yield  Bitter almond |
| 367* | Zhalantun  Inner Mongolia | High yield  Bitter almond | HL14* | Luanping  Hebei | High yield  Bitter almond |
| 368 | Zhalantun  Inner Mongolia | High yield  Bitter almond | HZ03 | Zhuolu  Hebei | High yield  Bitter almond |
| 375 | Zhalantun  Inner Mongolia | High yield  Bitter almond | HZ07 | Zhuolu  Hebei | High yield  Bitter almond |
| 381* | Zhalantun  Inner Mongolia | High yield  Late-maturing  Bitter almond | HZ09 | Zhuolu  Hebei | High yield  Bitter almond |
| 401 | Zhalantun  Inner Mongolia | Late-flowering  Bitter almond | HZ10 | Zhuolu  Hebei | High yield  Bitter almond |
| 404 | Zhalantun  Inner Mongolia | Late-flowering  Bitter almond | HZ11* | Zhuolu  Hebei | High yield  Bitter almond |
| 405 | Zhalantun  Inner Mongolia | Late-flowering  Bitter almond | HZ13 | Zhuolu  Hebei | High yield  Bitter almond |
| 406* | Zhalantun  Inner Mongolia | Late-flowering  Bitter almond | HZ14 | Zhuolu  Hebei | High yield  Bitter almond |
| 407* | Zhalantun,  Inner Mongolia | Late-flowering  Bitter almond | XB | Kazuo  Liaoning | High yield  Bitter almond |

^*^ indicated that the 40 accessions were selected for the optimal core collection constructed using the preferred sampling strategy with 25% sample size and Nei & Li genetic distance.

^#^ indicated that the Accession used for screening primers.
