# Supplementary material for: Selection of a core collection of Prunus sibirica L. germplasm by a stepwise clustering method using simple sequence repeat markers
Source: PLoS One. 2021 Nov 19;16(11):e0260097. doi: 10.1371/journal.pone.0260097 (PMC8604298; doi:10.1371/journal.pone.0260097)
Supplement: S2 Table — (DOCX) [file pone.0260097.s003.docx]

**S2 Table. The information of 30 SSR markers**

| Primer number | Forward primer sequence  (5'-3') | Reserve primer sequence  (3'-5') | Repeat  unit | Product  size |
| --- | --- | --- | --- | --- |
| L23 | CAAATGTTGACATCTTGACGTGGT | TTGGTCTGTATTTGTGACGTGGTT | ATC | 160 |
| L25 | TACCAGCTAGCTATGACCCCAAAC | ACCGAAACAACCAGATTTGATCTC | ATGT | 117 |
| L46 | GCAGCCTTGAAAATCCCAAAA | GGCATGAGATGCACTATTTGACAC | GTA | 157 |
| L49 | AGATCCTAGGCTGGAAGGCTCTAA | TCAGCCACTGCAAATAGCAATCTA | GTT | 158 |
| L62 | CTGGCAATGGCATTTATGTTGTAG | TTACCCTACCATCACCATGTAACG | TCCTCG | 147 |
| L62H | TTCCTTGTCCTAAGCTTTGGTGTC | AATCAACCTCGACATGGAAAGTGT | TAT | 108 |
| L7 | TTAGGGTACATGACACCAAGACGA | GCCAGGAATATCTTAGGGTTGGTT | AAG | 119 |
| L70H | CCACTCTCGTTTTACTTCCCAAGA | GGGGTGCATATAGATTTGAAGCAG | TCT | 144 |
| L75 | GCTGTTTGCATTGGTCCATACTCT | CACTCAACTTATTCATCCAGACTCCA | TTC | 151 |
| L79H | TAGGGATGAGCTGGCTGTTAGTTT | CCATGCCCAGGCCTATATAGAAGTA | TC | 160 |
| P21 | GGGTTAGAGGTTCGTCGGAGTAGT | AAGCTCCACATTTCTTCATTGCTC | GA | 112 |
| P3 | AGGGCTTTCATTCCTTTAAGTTGG | GGGAGGAGACGAGTAGGGTAGAAA | ATG | 144 |
| P40H | TTTGGTAAAAGACAACGACCCACT | TCCAACTCACACCCAAGTGATAGA | GT | 155 |
| P57H | CGCTATGGGGTAGGTTGTACATGA | CCCAAATATTTCAGGACCACAAGA | TC | 140 |
| X11H | TATATGTCAATGCTTGCACCCAAC | ATATCAACCTTGTAGCCCCCAACT | AG | 123 |
| X15H | TCCTTGCCTAGTCCAGCTTAAAAA | TGGCAGATGGAAACTCAAAGGTAT | AG | 151 |
| X19H | CGAACCTCTCCCTCTTTCTCTCTC | AAACCTAGCCCAAATCCTCCTTC | AG | 149 |
| X32H | TACGCTTCAAACAAGTACAGCAGC | TGAGGCGAGAGAATAGATAAGAAGGA | AGC | 150 |
| X38H | ATCCTAAAGGGCATCCCTCAAATA | TAGGGTTAAACGCCAAAATGTCAC | AT | 151 |
| X42H | GAAATACAATGCGACGTACACCCT | GGGGACATCATCTTGTAGTGCTG | AT | 150 |
| X44H | GAGGGATCTCTCAATAGAAGGGGA | TGGAGTACTCTCAATGCACATGCT | AT | 122 |
| X47 | ATCCGAATCCGATCGATTAAGTCT | CAAGTCCCTTCATGTTGTTCTGTG | CAGTC | 144 |
| X58H | AGTGGGTAATGTGGCGAAGTAGAG | GTGTTTCTTCCATCTCCAGAAGCTA | CT | 156 |
| X70 | AATTTGCAGAGACACCAAGGATG | GAGACTCTGACGACGGTTGAAGAT | CT | 147 |
| X87 | GGCCAGCCTCTTACTCAATAGACA | GTCGTCTAAACACAACACCCAACA | GGA | 124 |
| X8H | GTGTTGGTGTTTGGAGGTTTTCTC | GGGACATCCTTTAGGGTCCACTAC | AAT | 123 |
| Y48 | CAGGATTCTGGAACAAGGAAGAGA | GAGAGGAGGTAGTGGGTGTCTTGA | CAC | 141 |
| Y5 | AAGGAGTGCAAGAATGAGTGAACC | GCAAGCCTTCTTCATATAGAGCCA | AT | 148 |
| Y50 | ATATCGCACACTGCAAACACTAGC | CGATTGCCATGGTCACTATTCTTA | CAT | 160 |
| Y65 | GAGAAGGAGACGAAGCTGTGAAAG | ACGAAATAGCGTCCAGATTCAATG | GA | 159 |
