# Supplementary material for: Selection of a core collection of Prunus sibirica L. germplasm by a stepwise clustering method using simple sequence repeat markers
Source: PLoS One. 2021 Nov 19;16(11):e0260097. doi: 10.1371/journal.pone.0260097 (PMC8604298; doi:10.1371/journal.pone.0260097)
Supplement: S3 Table — (DOCX) [file pone.0260097.s004.docx]

**S4 Table. Diversity index at 30 SSR loci in *Prunus sibirica***

| Loci | *Na* | *Ne* | *I* | *PIC* | *H* |
| --- | --- | --- | --- | --- | --- |
| L23 | 24 | 9.469 | 2.564 | 0.886 | 0.894 |
| L25 | 13 | 6.598 | 2.046 | 0.846 | 0.848 |
| L46 | 35 | 12.756 | 2.876 | 0.923 | 0.922 |
| L49 | 31 | 13.638 | 2.904 | 0.924 | 0.927 |
| L62 | 15 | 6.31 | 2.139 | 0.834 | 0.842 |
| L62H | 16 | 6.062 | 2.076 | 0.82 | 0.835 |
| L7 | 12 | 8.539 | 2.267 | 0.883 | 0.883 |
| L70H | 34 | 12.08 | 2.881 | 0.912 | 0.917 |
| L75 | 13 | 5.853 | 2.007 | 0.818 | 0.829 |
| L79H | 36 | 12.445 | 2.971 | 0.925 | 0.920 |
| P21 | 18 | 6.639 | 2.126 | 0.835 | 0.849 |
| P3 | 14 | 5.75 | 2.084 | 0.863 | 0.826 |
| P40H | 16 | 10.393 | 2.507 | 0.897 | 0.904 |
| P57H | 11 | 4.31 | 1.653 | 0.733 | 0.768 |
| X11H | 29 | 12.796 | 2.808 | 0.923 | 0.922 |
| X15H | 25 | 10.38 | 2.632 | 0.907 | 0.904 |
| X19H | 23 | 2.859 | 1.808 | 0.64 | 0.650 |
| X32H | 9 | 2.431 | 1.17 | 0.529 | 0.589 |
| X38H | 17 | 7.049 | 2.235 | 0.852 | 0.858 |
| X42H | 22 | 6.719 | 2.31 | 0.853 | 0.851 |
| X44H | 23 | 12.865 | 2.762 | 0.922 | 0.922 |
| X47 | 18 | 9.578 | 2.494 | 0.887 | 0.896 |
| X58H | 24 | 10.623 | 2.591 | 0.902 | 0.906 |
| X70 | 24 | 7.298 | 2.396 | 0.855 | 0.863 |
| X87 | 15 | 7.237 | 2.221 | 0.849 | 0.862 |
| X8H | 18 | 4.576 | 1.991 | 0.767 | 0.781 |
| Y48 | 21 | 6.339 | 2.299 | 0.833 | 0.842 |
| Y5 | 15 | 6.324 | 2.152 | 0.833 | 0.842 |
| Y50 | 15 | 10.545 | 2.502 | 0.907 | 0.905 |
| Y65 | 21 | 7.374 | 2.374 | 0.853 | 0.864 |
| Mean | 20 | 8.194 | 2.328 | 0.847 | 0.854 |

*N_a_*: Observed number of alleles, *N_e_*: Number of effective alleles, *I*: Shannon’s information index, *PIC*: Polymorphic information content. *H*: Nei's gene diversity,
