# Supplementary material for: Selection of a core collection of Prunus sibirica L. germplasm by a stepwise clustering method using simple sequence repeat markers
Source: PLoS One. 2021 Nov 19;16(11):e0260097. doi: 10.1371/journal.pone.0260097 (PMC8604298; doi:10.1371/journal.pone.0260097)
Supplement: S4 Table — (DOCX) [file pone.0260097.s005.docx]

**Table S5. The genetic diversity of the initial collection and 30 core subsets**

| **Sample**  **size** | **Genetic**  **distance** | **N_a_** | | **N_e_** | | **I** | | **PIC** | | **H** | |
| --- | --- | --- | --- | --- | --- | --- | --- | --- | --- | --- | --- |
|  |  | **RS** | **PS** | **RS** | **PS** | **RS** | **PS** | **RS** | **PS** | **RS** | **PS** |
| 10% | SM | 11 | 12 | 7.539 | 8.444 | 2.141 | 2.250 | 0.843 | 0.850 | 0.850 | 0.860 |
|  | JD | 11 | 13 | 6.993 | 7.773 | 2.087 | 2.203 | 0.841 | 0.851 | 0.843 | 0.850 |
|  | ND | 11 | 12 | 6.928 | 8.573 | 2.093 | 2.256 | 0.839 | 0.863 | 0.841 | 0.868 |
| 15% | SM | 13 | 14 | 7.729 | 8.562 | 2.207 | 2.284 | 0.845 | 0.852 | 0.849 | 0.854 |
|  | JD | 12 | 13 | 7.421 | 8.050 | 2.138 | 2.237 | 0.841 | 0.855 | 0.843 | 0.856 |
|  | ND | 13 | 14 | 7.768 | 8.766 | 2.196 | 2.320 | 0.850 | 0.862 | 0.844 | 0.870 |
| 20% | SM | 14 | 15 | 7.702 | 8.481 | 2.223 | 2.287 | 0.842 | 0.850 | 0.848 | 0.864 |
|  | JD | 13 | 15 | 7.340 | 8.739 | 2.140 | 2.324 | 0.836 | 0.860 | 0.838 | 0.866 |
|  | ND | 13 | 15 | 7.579 | 8.822 | 2.186 | 2.321 | 0.842 | 0.863 | 0.847 | 0.868 |
| 25% | SM | 16 | 16 | 8.272 | 8.817 | 2.298 | 2.329 | 0.847 | 0.855 | 0.858 | 0.866 |
|  | JD | 14 | 16 | 8.225 | 8.862 | 2.260 | 2.335 | 0.850 | 0.861 | 0.856 | 0.868 |
|  | ND | 15 | 16 | 8.104 | 9.282 | 2.271 | 2.371 | 0.853 | 0.868 | 0.857 | 0.870 |
| 30% | SM | 16 | 16 | 8.229 | 8.393 | 2.302 | 2.291 | 0.849 | 0.847 | 0.857 | 0.857 |
|  | JD | 15 | 16 | 7.804 | 8.350 | 2.230 | 2.295 | 0.842 | 0.851 | 0.849 | 0.857 |
|  | ND | 15 | 16 | 7.791 | 8.934 | 2.244 | 2.343 | 0.847 | 0.861 | 0.850 | 0.865 |
| Mean | | 13 | 15 | 7.695 | 8.590 | 2.201 | 2.296 | 0.844 | 0.857 | 0.849 | 0.863 |
| STDV | | 1.727 | 1.502 | 0.415 | 0.370 | 0.069 | 0.045 | 0.005 | 0.006 | 0.006 | 0.006 |
| CV | | 0.128 | 0.103 | 0.054 | 0.043 | 0.032 | 0.020 | 0.006 | 0.007 | 0.007 | 0.007 |
| Initial collection | | 20 | | 8.194 | | 2.328 | | 0.847 | | 0.854 | |

PS: Allele preferred strategy, RS: Random strategy, ND: Genetic distance using Nei & Li genetic similarity coefficient, SM: Genetic distance using simple matching coefficient, JD: Genetic distance using Jaccard genetic similarity coefficient, N_a_: Number of observed alleles, N_e_: Number of effective alleles, I: Shannon’s information index, PIC: Polymorphic information content. H: Nei's gene diversity, STDV: standard deviation, CV: coefficient of variability
